# RNA-seq analysis provides insights into cold stress responses of *Xanthomonas citri* pv. *citri*

**DOI:** 10.1186/s12864-019-6193-0

**Published:** 2019-11-06

**Authors:** Jin-Xing Liao, Kai-Huai Li, Jin-Pei Wang, Jia-Ru Deng, Qiong-Guang Liu, Chang-Qing Chang

**Affiliations:** 1 Integrative Microbiology Research Centre, South China Agricultural University, No. 483 Wushan Road, Tianhe Guangzhou, 510642 People’s Republic of China; 2 Department of Plant Pathology, Guangdong Province Key Laboratory of Microbial Signals and Disease Control, South China Agricultural University, No. 483 Wushan Road, Tianhe Guangzhou, 510642 People’s Republic of China

**Keywords:** *Xanthomonas*, Low temperature stress, Motility, Biofilm formation, Fatty acids, Metabolism

## Abstract

**Background:**

*Xanthomonas citri* pv*. citri* (*Xcc*) is a citrus canker causing Gram-negative bacteria. Currently, little is known about the biological and molecular responses of *Xcc* to low temperatures.

**Results:**

Results depicted that low temperature significantly reduced growth and increased biofilm formation and unsaturated fatty acid (UFA) ratio in *Xcc*. At low temperature *Xcc* formed branching structured motility. Global transcriptome analysis revealed that low temperature modulates multiple signaling networks and essential cellular processes such as carbon, nitrogen and fatty acid metabolism in *Xcc*. Differential expression of genes associated with type IV pilus system and pathogenesis are important cellular adaptive responses of *Xcc* to cold stress.

**Conclusions:**

Study provides clear insights into biological characteristics and genome-wide transcriptional analysis based molecular mechanism of *Xcc* in response to low temperature.

## Background

Plant diseases cause significant crop losses worldwide and development of effective disease control requires understanding the mechanisms of plant diseases [1]. Biological and non-biological factors can contribute in the development of plant diseases. Plant-pathogen interaction mediates biological factors of plant diseases. Environmental factors drive pathogens to adjust in the adverse environment to develop plant diseases [2]. Current advancements in phytopathology have provided extensive knowledge about host-pathogen relationship and environment [3, 4].

Low temperature is one of the most prevalent abiotic stresses. Different mechanisms among species facilitate to adapt during temperature changes and plant responses to cold stress have been extensively studied [5–8]. Cellular mechanisms such as RNA processing and nucleocytoplasmic transport play crucial roles in plant stress [9]. Ca^2+^ signaling pathway and salicylic acid (SA) also participate in responding to low temperature stress [10–12].

Impact of low temperature in the regulation of bacterial physiology has been reported. For example, *L. monocytogenes* was reported to evolve multiple adaptive response pathways under cold stress including change in the composition of membrane fatty acids to regulate membrane fluidity [13, 14]. *E. coli* adapts to low temperature environment by increasing the ratio of straight-chain unsaturated fatty acids (SCUFA) to straight-chain saturated fatty acids (SCFAs) [15]. In *Bacillus subtilis,* stress response to low temperatures involve proteins of translation machinery and membrane adaptation [16]. In general, bacteria adapt to low temperature environment by regulating several cellular factors such as fatty acid desaturases [17], cold shock proteins (CSPs) [18] and transcriptional regulators [14, 19–21].

Gram-negative bacteria, *Xanthomonas* is a pathogen of about 400 plant hosts including rice, citrus, banana, cabbage, tomatoes, pepper and beans [22]. *Xanthomonas citri* pv. *citri* is an important pathogen that causes citrus canker and has an optimum growth temperature of 20–30 °C with minimum range of 5–10 °C and the highest of 35 °C. At high temperature, *Xcc* rapidly reproduces in host tissues to cause immense proliferation of host cells resulting in the expansion and rupture of epidermal tissue, suberification and mass death of plant tissues [23]. China, Brazil, U.S.A., India, Mexico, and Spain are world’s leading citrus growing countries. In China, citrus plants are mainly grown in southern China [24] and autumn temperature decreases up to 15 °C [25]. Although, plant response to cold stress has been extensively studied [5–8], but limited information is available about the impact of low temperature on plant pathogen, *Xanthomonas*. To gain insight into the molecular mechanisms of *Xcc* in response to low temperature, RNA-seq technology was employed along with physiological experiments to examine the spectrum and impact of low temperature on gene expression profiles and physiological changes in *Xcc*.

## Results

### Negative effects of low temperature on *Xcc* growth

**Fig. 1:**
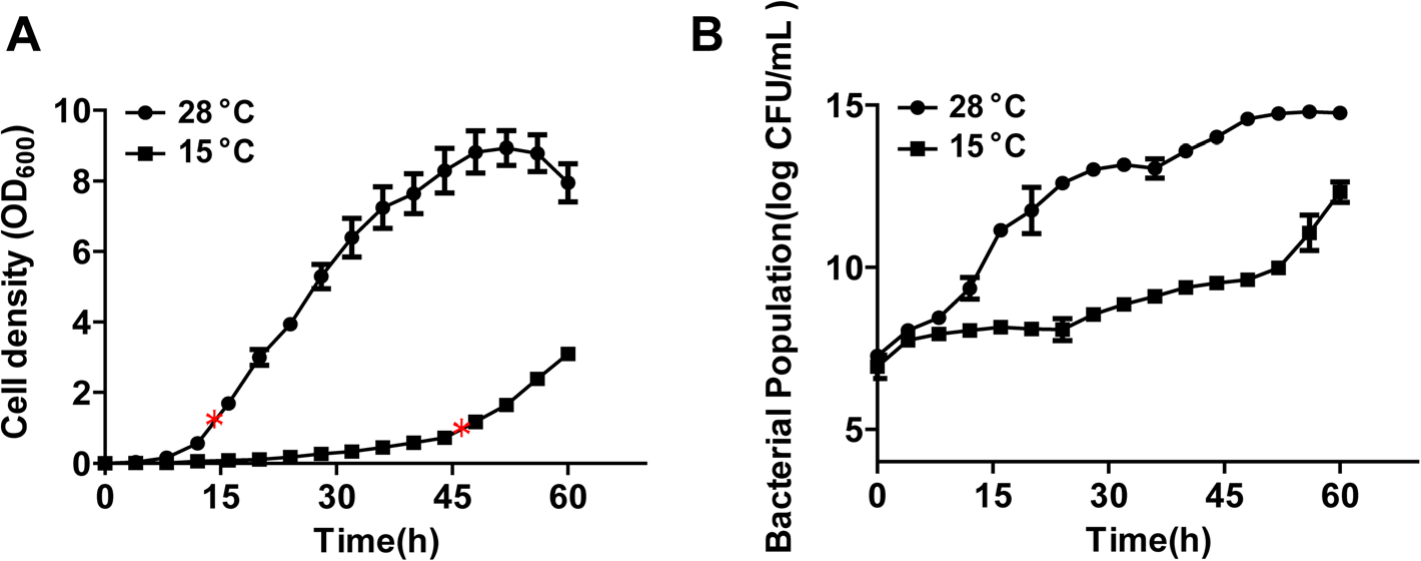
Reduced *Xcc* strain growth at low temperatures. **a** Growth curves of bacterial strains in rich YEB medium at 28 °C and 15 °C. “*” indicates the growth stage in which RNA extraction was performed. **b** Colony forming units (CFU) of *Xcc* strains during different growth phases at 15 °C and 28 °C. Error bars mean ± standard deviation (*n* = 3). All experiments were repeated three times with similar results

Temperature is a crucial environmental factor that determines the growth of pathogens [26]. To investigate the effect of temperature change on *Xcc*, the growth of wild-type strain was anlyzed (OD 600 nm) at 28 °C and 15 °C in YEB medium. *Xcc* exhibited slower growth at low temperature and different lag phases at different temperatures (Fig. 1a). Colony forming units (CFU) of *Xcc* strain in different growth phases at 15 °C and 28 °C were measured by dilution plate count method, which revealed significant effect of low temperature on *Xcc* growth (Fig. 1b)*.*

### Effects of low temperature on swarming motility and biofilm formation of *Xcc*

**Fig. 2:**
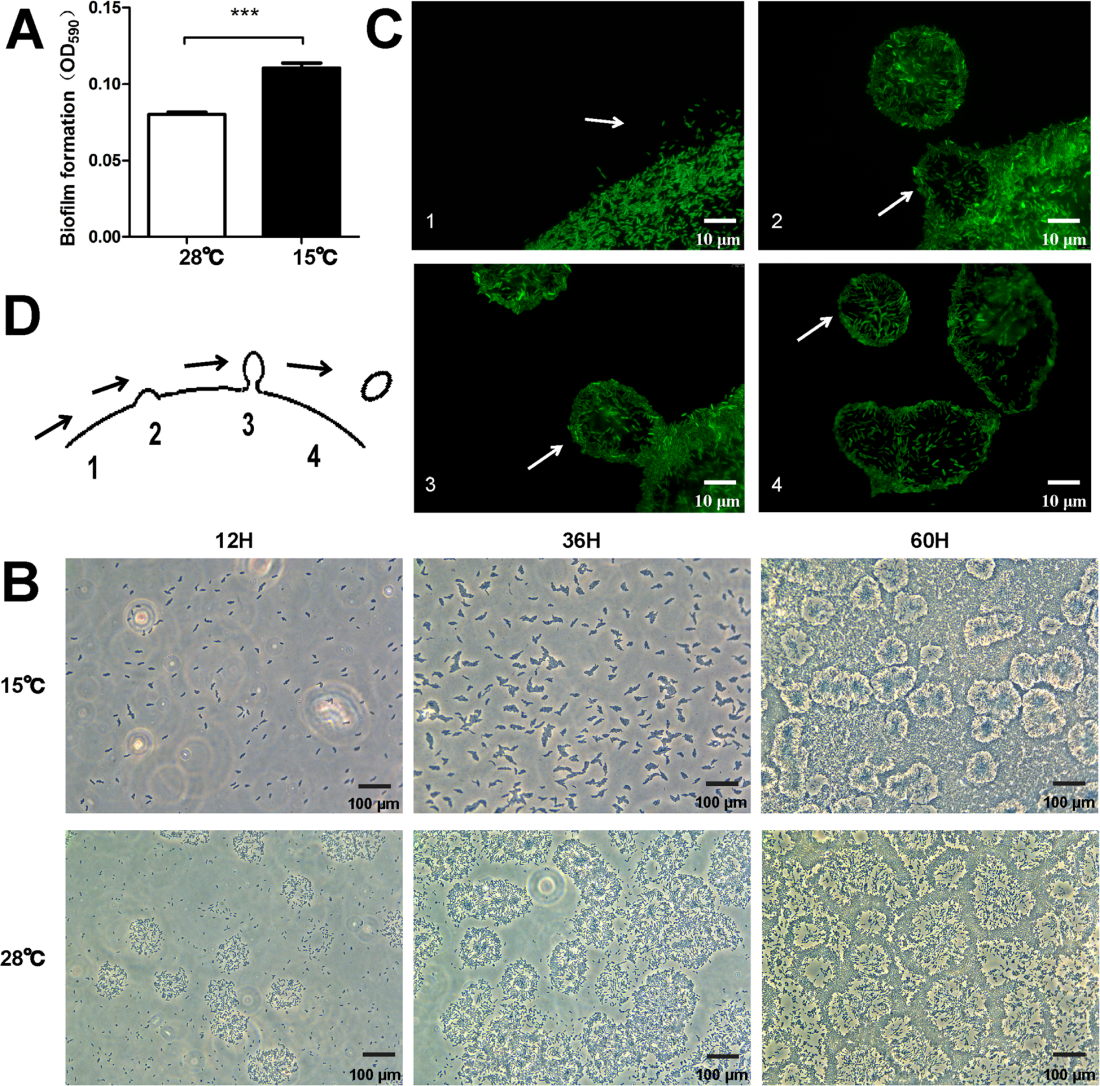
Low temperatures effected *Xcc* biofilm formation. **a** *Xcc* biofilm formation at 28 °C and 15 °C. Statistical analysis was performed in GraphPad Prism software (“***” stands for *p*-value < 0.001). Results are from one representative experiment of three independent experiments. **b** Several stages of *Xcc* biofilm formation on the interstitial surfaces between glass slides and nutrient-agar medium at different temperatures. **c** Image of *Xcc* biofilm formation stage. **d** Schematic representation of the images taken in C

**Fig. 3:**
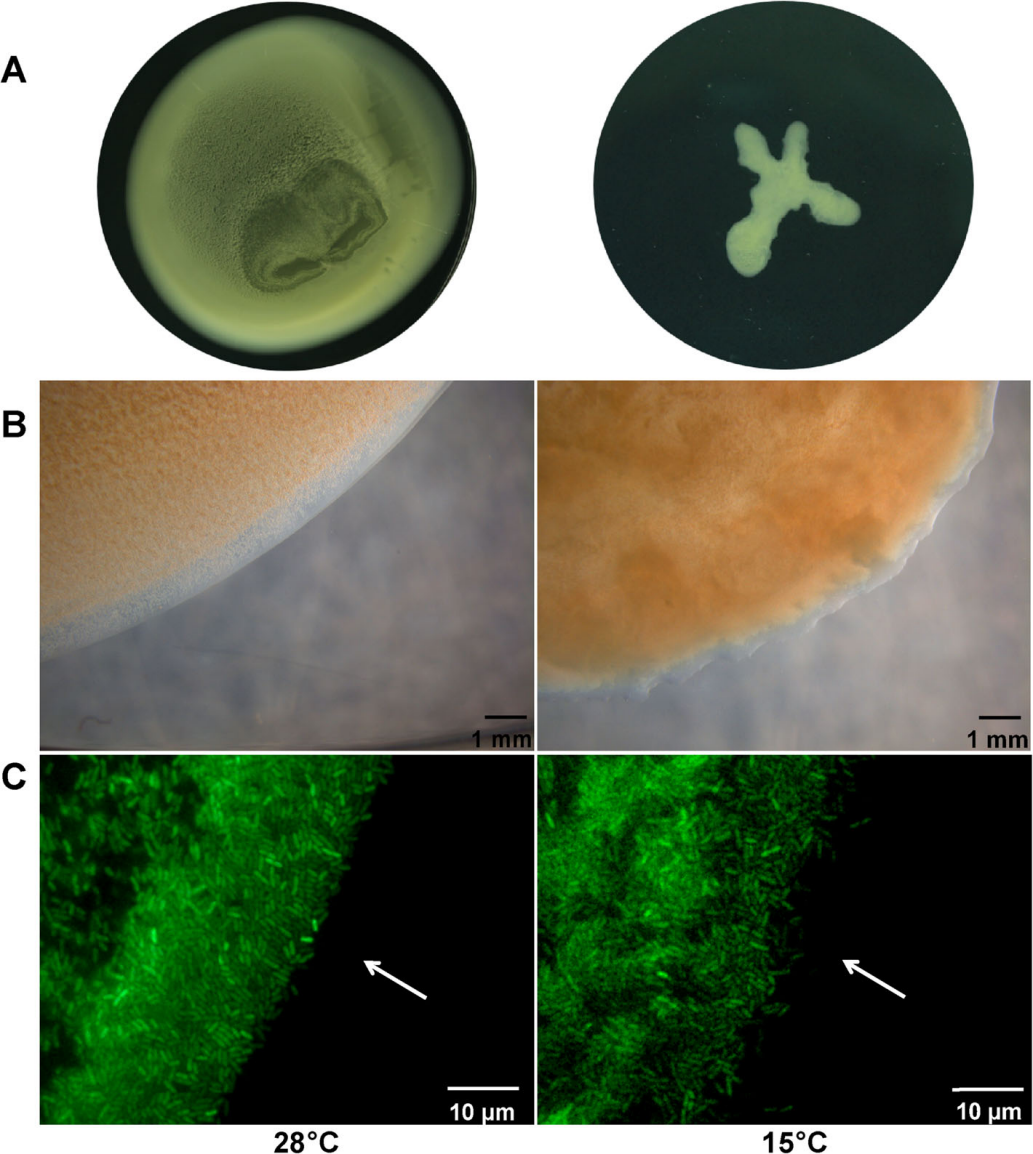
Low temperatures effected *Xcc* swarming motility. **a** Swarming motility of *Xcc* wild- type strain on rich YEB medium plates at 28 °C and 15 °C after 3 days. **b** The characteristic image of *Xcc* colonies edge morphology were captured by inverted microscope at 28 °C and 15 °C. **c** Microscopic images of *Xcc* edge expressing green fluorescent protein. Images were obtained under an inverted fluorescence microscope at 100X magnification

Motility is an important virulence trait of bacterial pathogens as it facilitates attachment to host surfaces and colonization of different environments [27, 28]. Biofilms are essential for environmental persistence especially when organisms are undergoing temperature changes. Comparative analysis of *Xcc* motility and biofilm formation at 28 °C and 15 °C revealed that biofilm formation was increased at low temperature (Fig. 2a). At different temperatures, *Xcc* biofilm formation was also observed on interstitial surfaces between glass slides and nutrient-agar medium. At low temperature bacteria densely gathered to form closely packed biofilm layer (Fig. 2b). These biofilms were observed as dynamic communities that split into small groups of “pioneer” cells to colonize a new environment [29] (Fig. 2c). Colonization phase occurred at the temperatures higher than 28 °C whereas significantly reduced swarming mobility of *Xcc* was noted at low temperature (Fig. 3a, Additional file 11: Figure S1). Colony shapes were generally round having smooth borders without bacterial extensions on 0.3% agar plates and formed branching structures at 15 °C. Edge morphology of *Xcc* colonies at different temperatures was studied under inverted microscopes (Fig. 3b). The shape of colonies and direction of motion revealed that *Xcc* presented an outward protrusion at 15 °C. Formation of an uneven and indefinite boundary at low temperature was also observed under the microscope (Fig. 3c). This implies a unique way of *Xcc* to adapt in low temperature environment.

### Low temperature modulates UFAs of *Xcc*

**Fig. 4:**
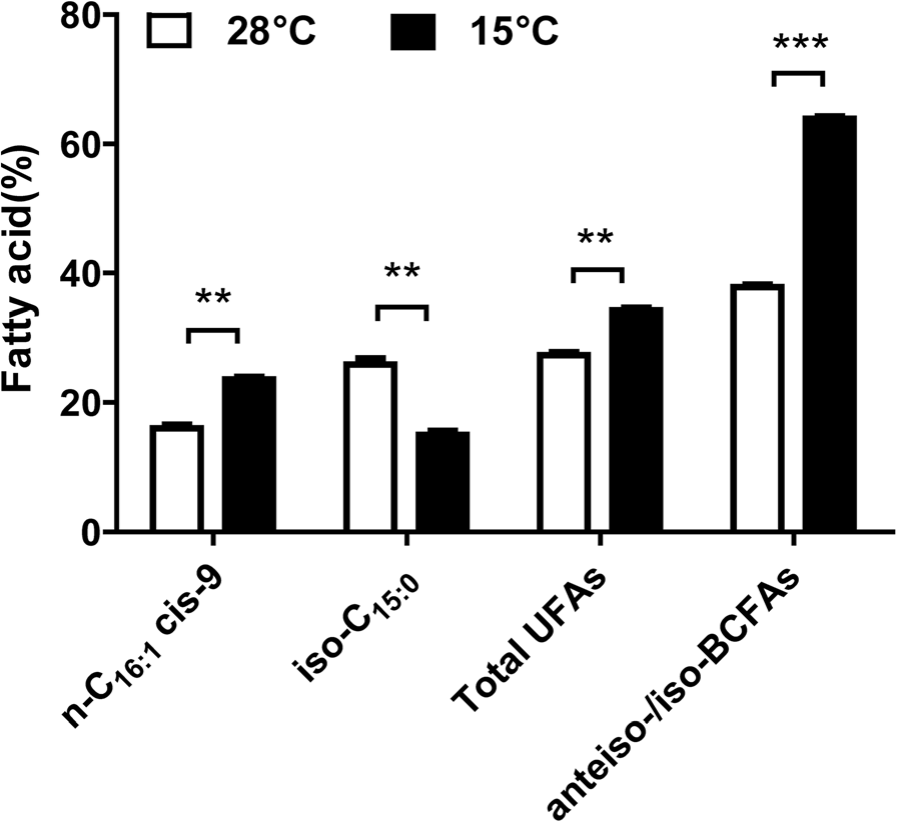
Differences in the compositions of *Xcc* fatty acid phospholipids at different temperatures. n-C_16:1_cis-9, cis-9-hexadecenoic acid; iso-C_15:0_,13-methyl-tetradecanoic acid; UFA, unsaturated fatty acid; BCFA, branched-chain fatty acid. Error bars, means ± standard deviations (n = 3). (“*” stands for p-value < 0.05, “**” stands for p-value < 0.01, “***” stands for p-value < 0.001)

In response to low temperatures, bacteria adjust membrane fatty acid composition to maintain membrane fluidity [13]. This might be dependent on whether the bacterial fatty acids are dominated by a mixture of straight-chain saturated fatty acids (SCFAs) and straight-chain unsaturated fatty acids (SCUFAs) or branch-chain saturated fatty acids (BCFAs). In many Gram-negative and some Gram-positive species, liquidity is mainly altered by changing the ratio of SCFAs to SCUFAs [30]. In order to maintain the membrane fluidity within optimal range of biological activities, lipid desaturases convert saturated fatty acids into unsaturated fatty acids or synthesize unsaturated fatty acids to increase lipid metabolism at low temperatures [31–34]. Species with a high proportion of BCFAs alter chain length and ratio of *anteiso* to *iso* fatty acids in response to low temperatures [35]. Little is known about the FA composition of *Xcc* at low temperatures. GC-MS analysis was conducted to find fatty acid composition of total lipid extracts of *Xcc,* grown in YEB medium at 28 °C and 15 °C. Pathogens were treated in the same state (OD_600_ = 0.8) at different temperatures. As shown in Table 1, major *Xcc* fatty acids at 28 °C included *iso*-C_15:0_ (26.82%), n-C_16:1_ *cis*-9 (16.56%) and *anteiso*-C_15:0_ (11.90%). Proportion of unsaturated fatty acids increased with the decrease in growth temperature, mainly due to the change in n-C_16:1_*cis*-9 percentage (Fig. 4). Growth at low temperature resulted in the decrease of *iso*-C_15:0_ percentage and increased percentage of *anteiso* to *iso* fatty acids ratio (Fig. 4). These results appeared consistent with the changes in membrane phospholipids for adapting to low temperature environment [36].

**Table 1:** Compositions of *Xcc* fatty acid phospholipids at different temperatures

Fatty acid (%)	28 °C	15 °C
n-C_12:0_	0.97 ± 0.20	0.97 ± 0.20
n-C_11:0_ 3-OH	1.94 ± 0.25	0.99 ± 0.30
n-C_14:0_ 3-OH	3.53 ± 0.30	5.36 ± 1.00
*iso*-C_14:0_	0.83 ± 0.05	0.73 ± 0.10
n-C_14:0_	2.10 ± 0.20	1.30 ± 0.10
n-C_16:0_ 3-OH	5.38 ± 0.40	3.14 ± 0.50
*iso*-C_15:0_	26.82 ± 2.50	14.30 ± 1.00
*anteiso*-C_15:0_	11.90 ± 2.00	13.71 ± 2.00
n-C_15:0_	4.23 ± 0.50	9.00 ± 0.05
*iso*-C_16:0_	2.25 ± 0.40	3.78 ± 0.20
n-C_16:1_ *cis*-9	16.56 ± 2.50	23.15 ± 2.00
n-C_16:0_	6.87 ± 0.50	7.04 ± 1.00
n-C_17:1_ *cis*-9	6.07 ± 1.00	4.29 ± 1.00
*iso*-C_17:0_	3.31 ± 0.50	3.95 ± 0.50
*anteiso*-C_17:0_	0.51 ± 0.15	0.91 ± 0.20
n-C_17:1_ *cis*-10	1.00 ± 0.15	2.55 ± 0.08
n-C_18:1_ *cis*-11	2.93 ± 0.20	2.24 ± 1.50
n-C_18:1_ *trans*-11	1.23 ± 0.20	1.59 ± 0.55
n-C_18:0_	1.57 ± 0.15	1.00 ± 0.75

^a^Cells were grown in YEB medium for 36 h at 28 °C or 15 °C. Total lipids were extracted and transesterified to fatty acid methyl esters and products were identified by GC-MS. Values represent percentages of total fatty acids and are means ± standard deviations of three independent experiments. *b* n-C14:0 3-OH, 3-hydroxyltetradecanoic; iso-C15:0, 13-methyl-tetradecanoic acid; anteiso-C15:0, 12-methyl-tetradecanoic acid; n-C15:0,pentadecanoic acid; iso-C16:0, 14-methyl-pentadecanoic acid; n-C16:1cis-9, cis-9-hexadecenoic acid; n-C16:0, hexadecanoic acid; iso-C17:1 cis-9, cis-9-15-methyl-hexadecenoic acid; iso-C17:0, 15-methyl-hexadecanoic acid; anteiso-C17:0, 14-methyl-hexadecanoic acid; n-C17:0 cyclo, 9,10-methylene hexadecanoic acid; n-C18:1, cis-11-octadecenoic acid; n-C18:0, octadecanoic acid

### Low temperature regulates the expression of genes involved in several functional categories

**Fig. 5:**
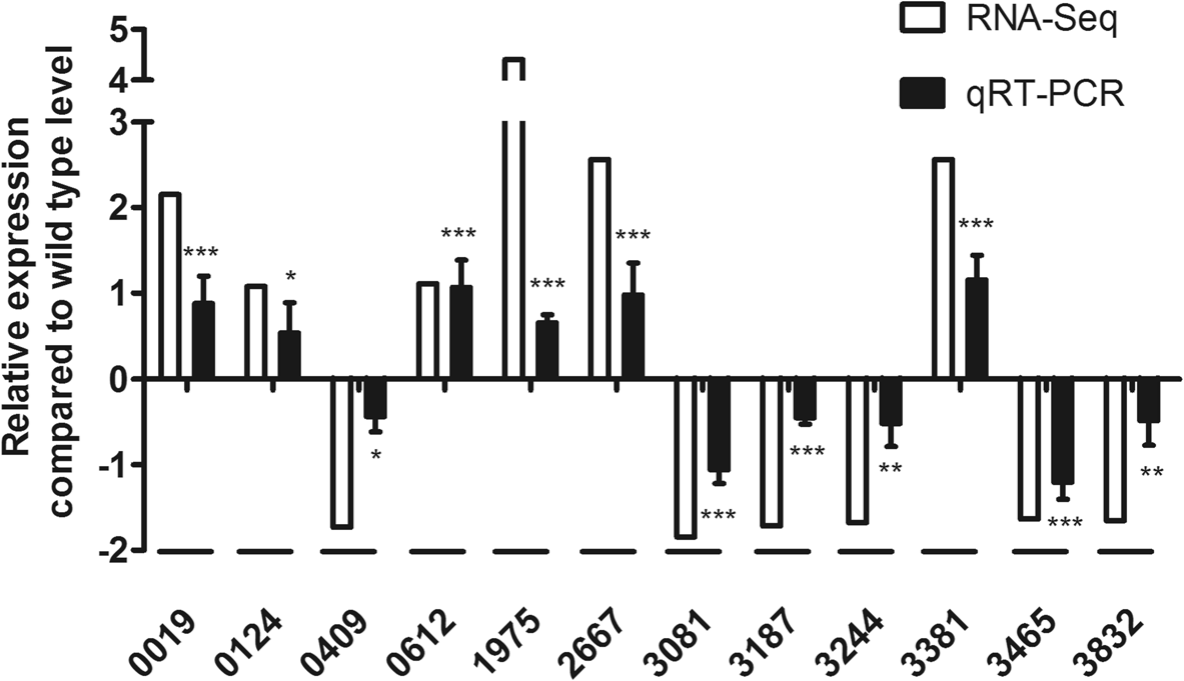
qRT-PCR analysis of 12 DEGs identified by RNA-Seq and compared between 28 °C and 15 °C. Y-axis indicates, relative expression to log2 fold change (log2FC), X-axis indicates selected candidate genes of DEGs. Error bars, means ± standard deviations (n = 3). Statistical analysis was performed between log2 fold change of qPCR experiment and 0. (“*” stands for p-value < 0.05, “**” stands for p-value < 0.01, “***” stands for p-value < 0.001)

In order to investigate the effect of low temperature on *Xcc*, RNA-Seq of *Xcc* was carried out at different temperatures. A total of 286.19 million and 288.68 million reads were generated from *Xcc* grown at 28 °C and 15 °C, respectively. The Q20 value of *Xcc* grown at 28 °C and 15 °C remained as 96.69 and 96.97%, respectively whereas the genome of *Xcc* was used as reference (NC_003919.1) [37]. Similar to the reference strain, the GC content of 28 °C and 15 °C samples was 65.06 and 63.25%. Clean reads were mapped to this genome at a ratio of 93.95 and 95.94% and approximately 83.45–84.60% of the total mapped reads were unique alignments for *Xcc* grown at 28 °C and 15 °C. Multi-aligned reads were removed and only unique reads were used for further analysis (Additional file 2: Table S2). Twelve genes identified in transcriptomic analysis were selected to further confirm differentially expressed genes (DEGs) with qRT-PCR. Expression trend of qRT-PCR analysis was consistent with RNA-Seq data (Fig. 5) and results indicated acceptable quality of *Xcc* RNA sequencing.

**Fig. 6:**
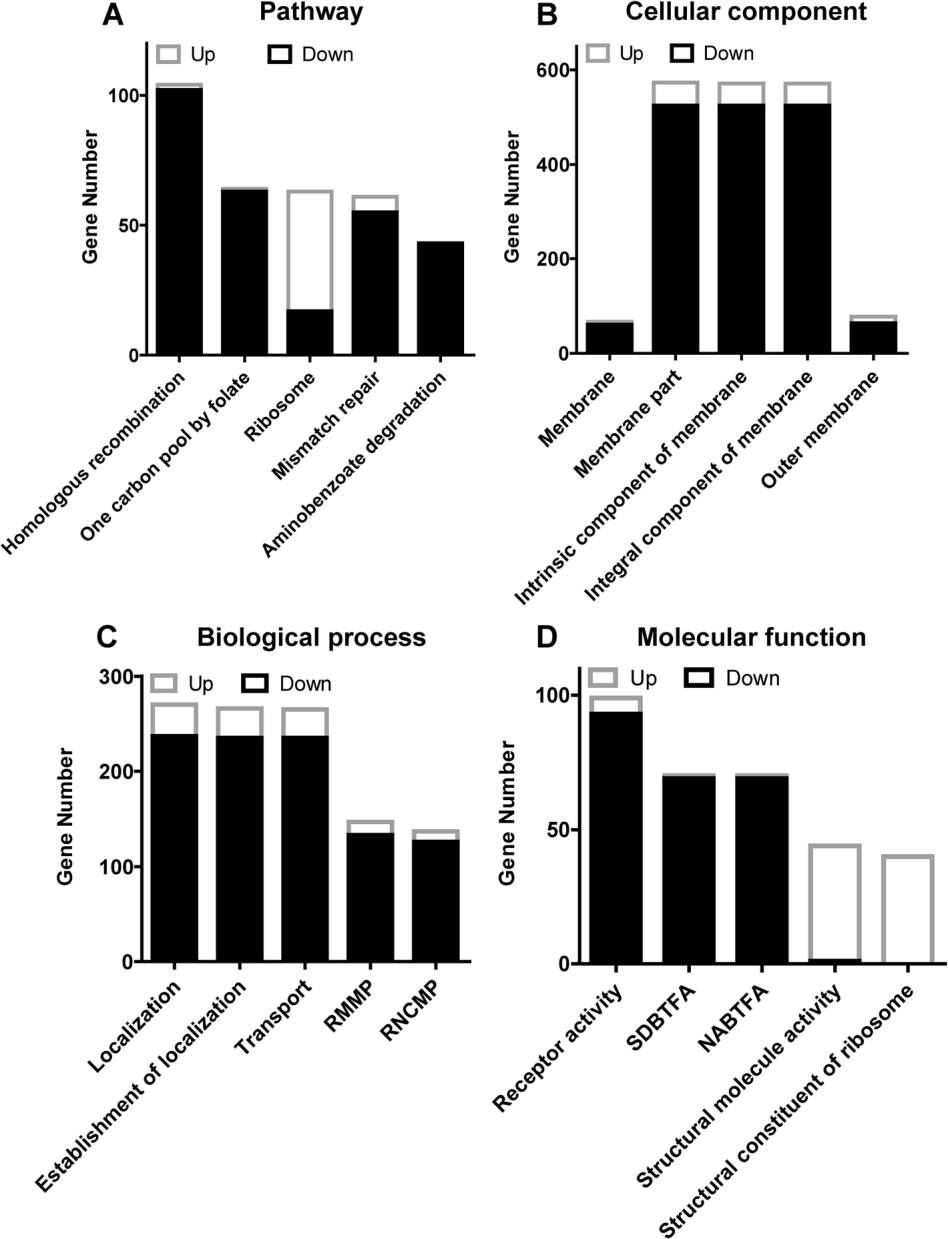
KEGG terms and classification of differentially expressed genes by gene ontology (GO) enrichment. **a** Top 5 enriched KEGG terms are shown on the graph. **b** Top 5 enriched GO terms of cellular component are shown on the graph. **c** Top 5 enriched GO terms of biological process are shown on the graph. **d** Top 5 enriched GO terms of molecular function are shown on the graph. RMMP: Regulation of macromolecule metabolic process. RNCMP: Regulation of nucleobas-containing compound metabolic process. SDBTFA: Sequence-specific DNA binding transcription factor activity. NABTFA: Nucleic acid binding transcription factor activity

To further explore the genes in response to low temperature, gene expressions were compared before and after low temperature treatments at a genome-wide level. A total of 2608 differentially expressed genes (DEG_S_) were identified at different temperatures of which 389 up-regulated and 2219 down-regulated (Additional file 3: Table S3). Based on this information, GO (Gene Ontology) annotation was carried out to classify the possible functions of DEG_S_ [38] and top 5 enriched GO terms of each category were determined (Fig. 6b, c, d). Top three GO terms of classified genes were membrane (659), membrane part (573) and integral component of membrane (571) for cell component category; receptor activity (99), sequence-specific DNA binding transcription factor activity (70) and nucleic acid binding transcription factor activity (70) for molecular function and transport (265), localization (266) and establishment of localization (270) for biological processes. In order to understand the biological function of DEGs, pathway enrichment analysis was performed at KEGG database to classify DEGs into 151 KEGG pathways and top 5 enriched pathways are presented in Fig. 6a. Three enriched pathways mostly affected by temperature include homologous recombination, one carbon pool by folate and ribosome.

### Response of *Xcc* genes involved in carbon and nitrogen metabolism at low temperature

Due to the effect of low temperature on *Xcc* growth (Fig. 1), DEGs involved in basal metabolism were further analyzed. Results of *Xcc* carbon metabolism at low temperature revealed that 90.7% genes, mainly involved in carbon and central carbon metabolism were down-regulated (Additional file 3: Table S3). Genes that encode enzyme catalyzing key chemical reactions for cell survival such as glucokinase, a-type carbonic anhydrase and bifunctional isocitrate dehydrogenase kinase/phosphatase were down-regulated. Five genes involved in the glycolysis pathway and pyruvic acid metabolism were up-regulated indicating that low temperature does not inhibit their activities (Additional file 4: Table S4). These results demonstrated that low temperature might block other pathways to limit energy for cell growth and metabolism. Analysis of DEGs involved in nitrogen metabolism revealed that 79.2% genes mainly including the components of cellular nitrogen compound biosynthetic process were down-regulated (Additional file 5: Table S5). Genes involved in nitrogen compound transport were simultaneously down-regulated resulting in the reduction of nitrogen absorption. Overall, results suggest that low temperature disrupts carbon and nitrogen metabolism in *Xcc*.

### Low temperature alters genes expression of flagellar and type IV pilus systems in *Xcc*

**Fig. 7:**
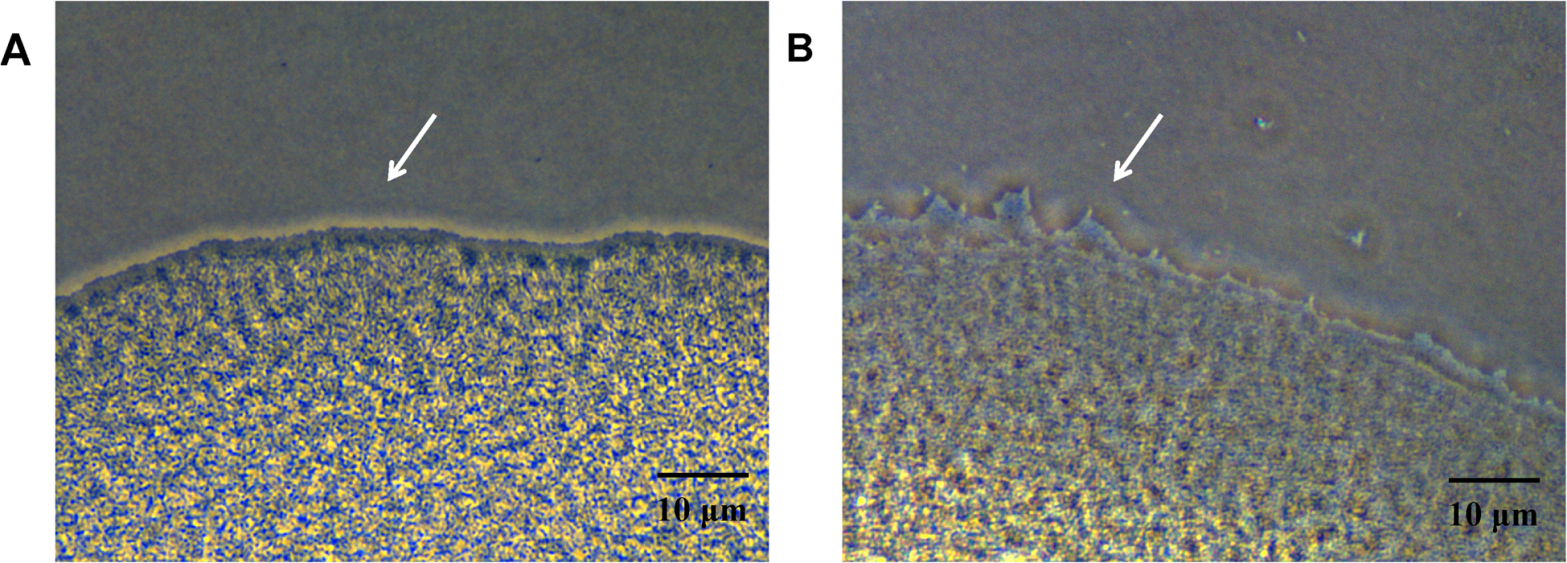
Low temperatures effected *Xcc* twitching motility. **a** Microanalysis of multi-cellular organization at the edges of twitching zones of *Xcc* cells at 28 °C. **b** Microanalysis of the multi-cellular organization at the edges of twitching zones of *Xcc* cells at 15 °C. Images were obtained under an inverted microscope at 40X magnification. All experiments were repeated three times with similar results

Significant differences in *Xcc* motility at different temperatures were observed (Fig. 2b, c). To further understand phenomenon at molecular level, DEGs associated with the flagellar system were analyzed. As expected, low temperature affected flagella assembly however varied effects of temperature on *Xcc* flagella assembly genes were observed (Additional file 6: Table S6). Low temperature treatment resulted in up-regulation of four genes and down-regulation of two genes suggesting that low temperature may disrupt flagella assembly of *Xcc.* Surprisingly type IV pilus systems, normally involved in bacterial cell adhesion to host cells and in bacterial cell motility [39], also responded to low temperature condition (Additional file 7: Table S7). The up-regulation of type IV pilus genes indicate their adaptation process to environmental pressure. To assess whether these changes in gene expression generate a temperature related motility phenotype in *Xcc*, the twitching motility pattern of this bacterium at 15 °C and 28 °C were tested. Microscopic analysis of twitching assay at low temperature depicted that multi-cellular organization at the edges of subsurface twitching zones of *Xcc* cells has blurred and irregular boundary lines (Fig. 7a, b). Taken together, results suggest that low temperature may disrupt flagella assembly and up-regulate type IV pilus genes expression leading to differential motility in *Xcc*.

### Membrane lipid metabolism-related genes are predominately down-regulated at low temperature *Xcc* treatment

**Fig. 8:**
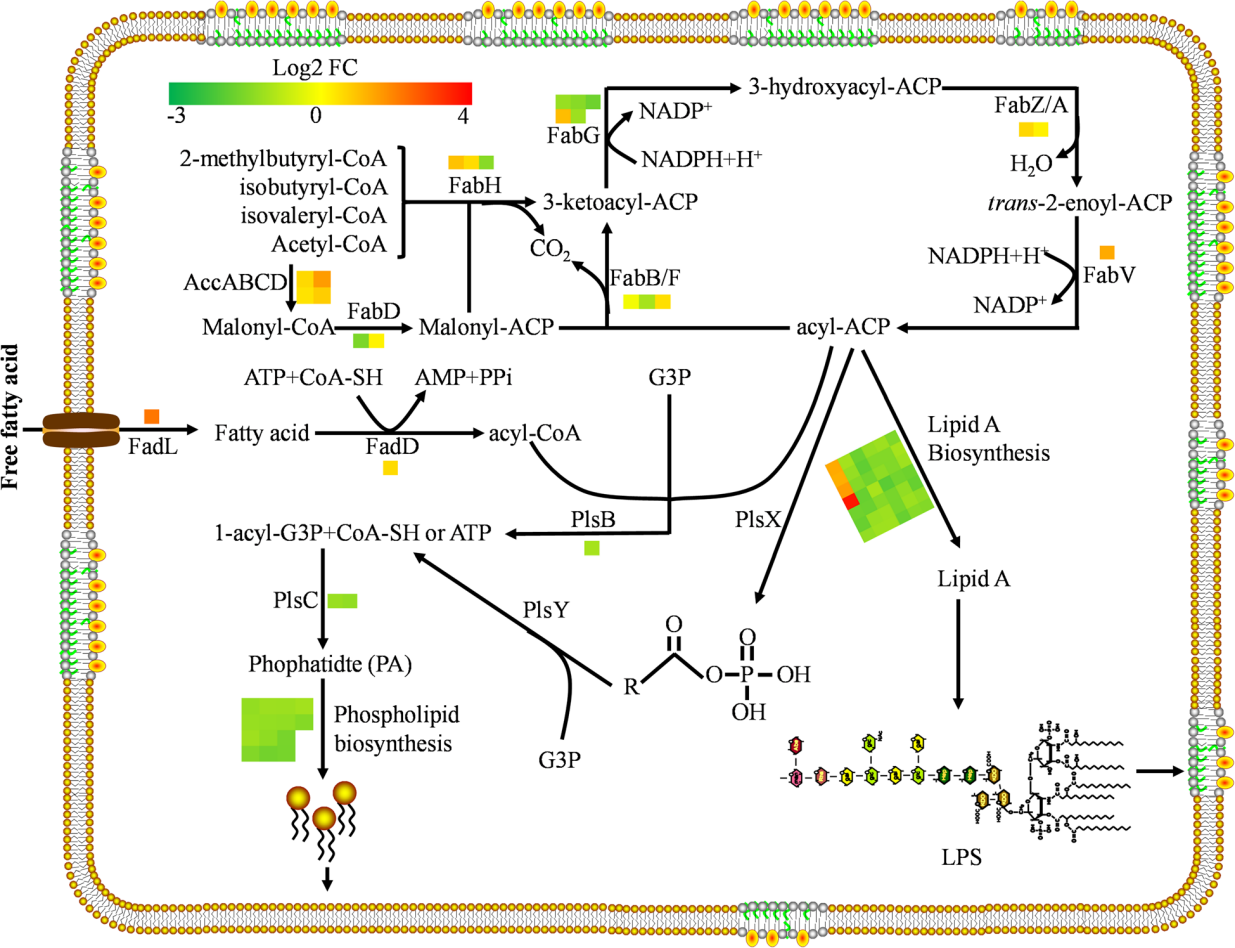
Membrane FA composition biosynthesis in response to low temperature in *Xcc*. One square denotes one gene in related reaction and its color implies the gene’s expression level. FC (Fold change of 15 °C/ 28 °C) Abbreviations: AccA, acetyl-CoA carboxylase, carboxyltransferase α-subunit; AccB, acetyl-CoA carboxylase, carboxybiotin carrier protein; AccC, acetyl-CoA carboxylase, biotin carboxylase; AccD, acetyl-CoA carboxylase, carboxyltransferase β-subunit; FabD, malonyl-CoA:ACP transacylase; FadD, acyl-CoA synthetase; FabH, 3-ketoacyl ACP synthase III; FabG, 3-ketoacyl-ACP reductase; FabZ, 3-hydroxyacyl-ACP dehydratase; FabA, 3-hydroxydecanoyl-ACP dehydratase/isomerase; FadL, long-chain fatty acid transport protein; FabV, enoyl-ACP reductase; FabF, 3-ketoacyl-ACP synthase II; FabB, 3-ketoacyl-ACP synthase I; plsB, sn-Glycerol-3-phosphate acyltransferase; plsX, sn-Glycerol-3-phosphate acyltransferase; plsY, sn-Glycerol-3-phosphate acyltransferase; plsC, lysophosphatidic acid acyltransferase; G3P, sn-glycerol-3-phosphate

Coordinated regulation of fatty acid biosynthesis is part of the normal bacterial response to environmental temperature changes (Table 1). As low temperature influenced UFAs, DEGs related to fatty acid biosynthesis, phospholipid synthesis and lipid A synthesis were analyzed (Fig. 8). At low temperature, 64 DEGs related to membrane lipid metabolism (Additional file 8: Table S8) and 88.9% genes were down-regulated. Results further demonstrated that change in the temperature affects membrane lipid metabolism related genes in *Xcc.* Thereby, changing the membrane phospholipid component of *Xcc* to adapt in low temperature environment. Specifically, 3-hydroxylacyl-ACP dehydratase/isomerase (FabA) and 3-ketoacyl-ACP synthase I (FabB) were not significantly up-regulated. The *fabB* and *fabA* genes encode key enzymes of classic anaerobic pathway of unsaturated fatty acid synthesis [40, 41]. Thus FabA and FabB may not essentially increase the synthesis of unsaturated fatty acids. However, long-chain fatty acid transport protein (FadL) was up-regulated, implying that more free fatty acid can be transferred from outside into the cells. These results strongly suggest that different temperatures affect the gene expressions related to membrane lipid metabolism that changes membrane phospholipid components in *Xcc*.

### Pathogenesis-associated genes in *Xcc* are negatively regulated by low temperature

This study explains that temperature change can affect bacterial virulence in Arabidopsis [4] and multiple cellular processes in *Xcc* (Fig. 6).Therefore, we analyzed the effect of low temperature on pathogenesis-associated genes expression in *Xcc*. Six pathogenesis related DEGs were influenced by low temperature treatment of whichone was up-regulated and 5 were down-regulated (Additional file 9: Table S9). Moreover, we further analyzed the genes related to pathogenesis secretion systems, which secrete degradation enzymes and toxins including type II (T2SS), type III (T3SS) and type IV (T4SS) secretion system. Results showed that 78.3% of genes related to these systems were down-regulated at low temperature (Additional file 10: Table S10). However, the expression of pathogenesis-associated genes was detected in rich medium, which might be different in other environments.

## Discussion

*Xanthomonas citri* pv*. citri* is a global pathogen of citrus plants, which directly reduces fruit quality and quantity. Environmental factors play important role in determining the outcome of plant-pathogen interactions and development of plant disease [42]. Low temperature is a common environmental factor and cold shock is known to restrict bacterial growth [43]. During the study we observed significantly effected *Xcc* growth rate at low temperature (Fig. 1). Transcriptomic analyses showed that low temperature down-regulated expression of genes involved in carbon and nitrogen metabolism but had little effect on genes related to glycolysis pathway and pyruvic acid metabolism (Additional file 3: Table S3, Additional file 4: Table S4). Contrarily, ribosomal pathway was up-regulated (Fig. 6a) implying that ribosomal proteins might have special functions at low temperature like other bacteria [44]. Based on global gene expression analysis we proposed metabolic pathways associated with the effects of temperature changes on *Xcc* growth.

Biofilms are strongly associated with pathogenesis. The persistence of *P. aeruginosa* in chronic lung infections of cystic fibrosis patients is related to biofilm formation, which enhances bacterial adhesion to the cell and evasion of host’s immune responses. Biofilm formation also facilitates antibiotic tolerance and limits eradication [45]. Similar to *Vibrio cholerae*, *Xcc* formed more and tighter biofilms at low temperature (Fig. 2b) that demonstrates a regulatory switch between planktonic growth and biofilm formation in response to environmental changes [46]. Moreover, typical late autumn temperature (15 °C) implies [25] that increased biofilm formation represents bacterial preparation for overwintering. At low temperature motility was significantly influenced and *Xcc* formed an uneven and indefinite colony boundary (Fig. 3b, c). Motility plays a vital role in the attachment and colonization of bacteria in appropriate locations at favorable times [27, 47]. Transcriptome analysis revealed diverse effects of low temperature on *Xcc* flagella assembly genes (Additional file 6: Table S6). Results suggested that low temperature could be disruptive to flagellar system and subsequently disorganizes flagellar assembly. Interestingly, low temperature stimulated the expression of genes related to type IV pilus systems (Additional file 6: Table S6). Microscopic analysis of twitching assays depicted that subsurface twitching zones of *Xcc* cells have blurred and irregular boundary lines at low temperatures (Fig. 7a, b). This is an uncharacterized aspect of *Xcc* and underlying mechanism is the focus of our studies. Moreover, type IV pilus participates in biofilm formation [48, 49] and that might be a potential pathway of *Xcc* to increase biofilm formation at low temperatures.

Membrane fluidity is essential for the survival of bacteria at low temperature [30] and bacteria have evolved various strategies to modulate membrane fluidity [50–53]. This study revealed that low temperature stimulates the biosynthesis of unsaturated fatty acids by increasing the percentage of n-C_16:1_*cis*-9 (Fig. 4). Further analysis indicated that increase in the proportion of unsaturated fatty acids might be due to overall change in the level of membrane phospholipid synthesis without involving FabA-FabB pathway. Lower growth temperature sharply reduced *iso*-C_15:0_ percentage and increased the ratio of *anteiso* to *iso* fatty acids (Fig. 4). It is an effective way to adjust the membrane fluidity for adapting at low temperature. However, *Xcc* mechanism of altering the ratio of *anteiso* to *iso* fatty acids at low temperature is unknown. Nevertheless, effect of temperature on the gene expression of FabH (XAC1964, XAC2571 and XAC1123) was noted (Fig. 8). FabH plays an important role in branched-chain fatty acids and carries out first condensation reaction in fatty acid biosynthesis pathway by using a variety of substrates [30, 54–57]. This might be a potential mechanism to alter the ratio of branched-chain fatty acids at low temperature through the selection of substrates. However, FabH pathway requires further investigation. Analysis of the genes related to phospholipid synthesis and lipid A synthesis in DEGs (Fig. 8) revealed that 88.9% genes were down-regulated (Additional file 8: Table S8) implying that more energy is used for basal metabolism of bacteria at low temperature.

Virulence gene expression in pathogenic bacteria is regulated by environmental parameters and temperature is the key factor [58]. Studies have shown that most plant pathogen virulence genes exhibit increased transcription below optimum temperatures [59]. However, we found that a large portion of genes related to *Xcc* pathogenesis were down-regulated at low temperature (Additional file 8: Table S8), including CRP-like protein Clp, adhesin, membrane protein, avirulence protein and EscJ/YscJ/HrcJ family type III secretion inner membrane ring protein. This might be a self-protective mechanism in bacteria as virulence factors are essential for the infection process only but not necessary under environmental stress. To ensure their survival, bacteria do not express virulence genes to save energy. Consistent with *P. syringae*, we found that low temperature influenced *Xcc* secretion system that might reduce the injection of effector proteins or toxins into host cells (Additional file 10: Table S10) [60]. Further investigation is required to elaborate this interesting observation. However, YEB medium used in this study is a rich medium that is not generally associated with pathogenicity inducing conditions. In future we will consider using different medium to mimic plant’s internal environment. Meanwhile, we speculate that low temperature may initiate additional uncharacterized mechanisms to control *Xcc* virulence.

## Conclusions

In short, physiological characteristics of *Xcc* at low temperature were examined and genome wide transcriptional analysis presented specific response of *Xcc* at low temperature. Results suggested that many biological processes participate to respond at low temperature including carbon, nitrogen and fatty acid metabolism. In addition, low temperatures influence motility, biofilm formation and expression of pathogenicity related genes. Data of this study provide insights into molecular mechanisms of *Xcc* to adapt at low temperature and present an experimental reference information for future research to control plant diseases by temperature-dependent strategies.

## Methods

### Bacterial strains, media cultures and growth conditions

*Xanthomonas citri* pv. *citri* was grown at 28 °C and 15 °C in YEB medium (10 g/L peptone, 5 g/L yeast extract, 5 g/L NaCl, 5 g/L sucrose, 0.25 g/LMgSO_4_, pH 7.0) on a rotary shaker (180 rpm). Bacterial growth in liquid medium at 28 °C and 15 °C was determined by measuring optical density at 600 nm (OD_600_) and plating colony forming units (CFU). Briefly, *Xcc* strains were cultured overnight and then inoculated into fresh YEB medium. Strains were cultured at 28 °C and 15 °C at 200 rpm. Every 4 h, 1-mL of each culture was collected to measure the optical density at 600 nm (OD_600_) and estimate bacterial CFU by dilution plate counting. Three replicates were performed for each strain and bacterial growth curves were plotted according to average values.

### Swarming motility assay

Swarming motility assays were performed in YEB medium plates supplemented with 0.3% agarose. Bacteria were inoculated in the center of plates and incubated at 28 °C and 15 °C for 3 days before assessment. To conduct microscopic analysis of motility assays, *Xcc* strains were inoculated through a thin layer of nutrient agar supplemented with 2 mM CaCl_2_ and incubated at 28 °C for 2 days.

### Twitching motility assay

Twitching motility assays were performed according to Dunger et al. (2014) [49]. For microscopic analysis of motility assays, *Xcc* strains were stabbed through YEB medium (1% agar) supplemented with 2 mM CaCl_2_, covered with glass slides and statically incubated in a humidified chamber at 28 °C for 2 days.

### Biofilm formation assay

*Xcc* strains were grown in YEB to OD_600_ = 1.0, and inoculated into the 96-well plates for 36 h in rich YEB medium. Biofilms were rinsed three times with ddH_2_O, treated with 0.3% crystal violet for 15 min, again rinsed three times with ddH_2_O and re-suspended in 95% ethanol. Biomass was read in spectrophotometer at 590 nm. For microscopic analysis of biofilm formation assays, overnight cultures of *Xcc* strains expressing GFP in YEB medium were collected by centrifugation, washed with fresh medium and adjusted to OD_600_ = 1.0. Ten microlitre of this culture was diluted with 1 ml YEB medium, transferred into a thin layer of nutrient agar, covered with glass slides and statically incubated in a humidified chamber at 28 °C. The pBBR1MCS-5-GFP plasmid were used to transform *Xcc* strains into expressing green fluorescent protein.

### Analysis of fatty acid composition

To determine FA composition, *Xcc* strains were grown in YEB (OD_600_ = 0.8) at 28 °C and 15 °C. Fatty acid methyl esters were synthesized and extracted as previously described [61]. Cellular lipids were saponified by the addition of 1 mL sodium hydroxide/methanol solution at 100 °C and 800 rpm for 40 min. Fatty acids were methylated by the addition of 2 mL hydrochloric acid/methanol solution at 80 °C for 30 min and cooled to below 20 °C. Fatty acid methylesters were obtained by three extractions with 1 mL petroleumether. Solvent was removed under a stream of nitrogen and residues were dissolved in 100 μL of hexane. Crude extract was filtered through 0.22-μm Mini-star units and 2 μL extract was analyzed by gas chromatography-mass spectrometry (GC-MS system Agilent 5975c) with chromatographic column DB 5MS. Oven temperature was held at 100 °C for 5 min, changed at 10 °C/min to 200 °C and held for 5 min, then changed at 10 °C/min to 250 °C and held for 5 min. Electron impact ionization (EI^+^, 70 eV) was used for all samples. Mass spectrometry was carried out at 1 s/scan, m/z 35–500, 1 kV and data were analyzed by NIST 08 database.

### Sample preparation for RNA sequencing and differential expression analysis

*X. citri* strains were cultured on YEB medium at 28 °C and 15 °C, and collected at OD_600_ of 1.0 (4× 10^9^ CFU/ml). Bacterial strains were centrifuged and pellet was washed with DEPC water. RNA was extracted from the collected cells with Bacterial RNA Extraction Kit (Vazyme, China) according to the manufacturer’s instructions. RNA quality was checked in RNA-Nano (Agilent 2100) followed by RNA degradation and contamination verification on 1% agarose gel. Samples with RNA integrity number (RIN) more than 9 were used in downstream analysis. Clustering and sequencing were performed by Vazyme that employed spliced reads to determine exon connectivity. Gene FPKMs were computed by summing the FPKMs of transcripts in each gene group. FPKM stands for “fragments per of exon per million fragments mapped”, and it is calculated based on the length of fragments and reads count mapped to each fragment. FPKM also considers the effect of sequencing depth and gene length on reads counts, and is currently the most commonly used method for estimating gene expression levels [62]. Cuffdiff (v1.3.0) was used to calculate FPKMs of coding genes in each sample [63]. Cuffdiff provides statistical routines for determining differential expression in digital transcript or gene expression datasets by using a model based on negative binomial distribution. Genes with corrected *p* value less than 0.05 and absolute value of log2 (fold change) greater than or equal to 1 were assigned as significantly differentially expressed.

### Gene ontology and KEGG enrichment analysis

Analysis was conducted by putting all the differentially expressed genes to the Gene Ontology (GO) database (http://www.geneontology.org/) of each term mapping and calculated the number of genes in each term. It was followed by hypergeometric inspection to find out comparison with the entire genome, significantly enriched in the differentially expressed genes GO entries. Pathway significant enrichment analysis was based on KEGG pathway that applies hypergeometric test to find the pathway of significant enrichment in differentially expressed genes compared with the whole genome. In general, Corrected *p*-value < 0.05 indicated that differentially expressed genes were significantly enriched in KEGG pathway.

### Quantitative real-time PCR

Cells were collected at cell optical density (OD_600_) of 1.0 in YEB medium at 28 °C and 15 °C. Total RNA was extracted from at least two independent biological repeats by using Bacterial RNA Extraction Kit (Vazyme, China) according to the manufacturer’s protocols. Reverse transcription PCR was performed using a HiScript® II Q RT SuperMix for qPCR (+gDNA wiper) (Vazyme, China) according to the manufacturer’s instructions. Primers used in this assay are listed in Additional file 1: Table S1. Quantification of gene expression and melting curve analysis was conducted in 7300Plus Real-Time PCR System (Thermo Scientific) by using ChamQ™ Universal SYBR® qPCR Master Mix (Vazyme, China) according to the manufacturer’s instructions. Quantitative RT-PCR analysis of *rpoB* gene expression served as control. Relative expressions of target genes were calculated by following Quantitation-Comparative CT (ΔΔCT) method.

### Data analysis method

Statistical analyses were performed with one-way ANOVA test in GraphPad. Values and error bars represent means and SD.“*” stands for p-value < 0.05, “**” stands for p-value < 0.01, “***” stands for p-value < 0.001; ns stands for not significant.

## Supplementary information

**Additional file 1: Table S1.** Primers used in qRT-PCR for validating differentially expressed genes.

**Additional file 2: Table S2.** Overview of the sequencing and assembly

**Additional file 3: Table S3.** Differential gene expressions in *Xcc* at 15 °C.

**Additional file 4: Table S4.** List of genes related to carbohydrate metabolic process in *Xcc* regulated by temperature.

**Additional file 5: Table S5.** List of genes related to cellular nitrogen compound biosynthetic process in *Xcc* regulated by temperature.

**Additional file 6: Table S6.** List of genes related to flagellum in *Xcc* regulated by temperature.

**Additional file 7: Table S7.** List of genes related to pilus organization in *Xcc* regulated by temperature.

**Additional file 8: Table S8.** List of genes related to fatty acid metabolism in *Xcc* regulated by temperature.

**Additional file 9: Table S9.** List of genes related to *Xcc* pathogenesis regulated by temperature.

**Additional file 10: Table S10.** List of genes related to *Xcc* secretion regulated by temperature.

**Additional file 11: Figure S1**. Low temperatures effected *Xcc* swarming motility.

## Data Availability

RNA sequence dataset supporting the results of this article is available at NCBI under the bioproject no. PRJNA544888 with the Sequence Read Archive (SRA) accession no. SRP199587 (https://dataview.ncbi.nlm.nih.gov/?search=SUB5672622).
