# Supplementary material for: RNA-seq analysis provides insights into cold stress responses of Xanthomonas citri pv. citri
Source: BMC Genomics. 2019 Nov 6;20:807. doi: 10.1186/s12864-019-6193-0 (PMC6833247; doi:10.1186/s12864-019-6193-0)
Supplement: Supplementary file 1 — Additional file 1: Table S1. Primers used in qRT-PCR for validating differentially expressed genes. [file 12864_2019_6193_MOESM1_ESM.docx]

**Table S1 Primers for RT-qPCR**

| Gene ID | primer sequence |
| --- | --- |
| XAC0409 | F:5'CGGCGAGATGTTCAAGAAGG3' |
|  | R:5'ACGCGGAACTTGATGAACAC3' |
| XAC1975 | F:5'GTGGAAATCGGCAACAACCT3' |
|  | R:5'CTGTTGATGCCGATGGTCTG3' |
| XAC3081 | F: 5'ACTACGTCGGGCTCAATCAT3' |
|  | R:5'CAGGTATTCGGTGGGGATCA3' |
| XAC3187 | F:5'CTGCGTGCTGGTGGATTG3' |
|  | R:5'CAAACCGACGCGTGAGTTC3' |
| XAC3381 | F:5'ACGATGGCTTGCATAACGTC3' |
|  | R:5'AATCTCACGACCCTGCTTGA3' |
| XAC3465 | F:5'TCATGACGCTGGAAATGTGC3' |
|  | R:5'CGCTTGAGATGCTTGATGGT3' |
