# Supplementary material for: RNA-seq analysis provides insights into cold stress responses of Xanthomonas citri pv. citri
Source: BMC Genomics. 2019 Nov 6;20:807. doi: 10.1186/s12864-019-6193-0 (PMC6833247; doi:10.1186/s12864-019-6193-0)
Supplement: Supplementary file 2 — Additional file 2: Table S2. Overview of the sequencing and assembly [file 12864_2019_6193_MOESM2_ESM.docx]

**Table S2 Overview of the sequencing and assembly**

| **Sample ID** | **Clean reads** | **Clean bases** | **Q20** | **Clean GC content** | **Mapped reads** | **Unique match** |
| --- | --- | --- | --- | --- | --- | --- |
| XAC_28C | 19079274 | 2861891100 | 96.69% | 65.06% | 95.94% | 84.60% |
| XAC_15C | 19245458 | 2886818700 | 96.97% | 63.25% | 93.95% | 83.45% |
