# Supplementary material for: RNA-seq analysis provides insights into cold stress responses of Xanthomonas citri pv. citri
Source: BMC Genomics. 2019 Nov 6;20:807. doi: 10.1186/s12864-019-6193-0 (PMC6833247; doi:10.1186/s12864-019-6193-0)
Supplement: Supplementary file 3 — Additional file 3: Table S3. Differential gene expressions in Xcc at 15 °C. [file 12864_2019_6193_MOESM3_ESM.docx]

**Table S3. Differentially expressed genes in *Xcc* after 15**°C **treatment**

| Gene ID | Gene name | log2 fold change (15°C/ 28°C) | Gene Description |
| --- | --- | --- | --- |
| XAC_RS01325 | XAC0254 | -1.23437 | cation:proton antiporter |
| XAC_RS19715 | XAC3908 | -1.27681 | KR domain-containing protein |
| XAC_RS21430 | XAC4248 | -1.5296 | gluconolactonase |
| XAC_RS01905 | XAC0360 | 1.15303 | glycerol-3-phosphate dehydrogenase |
| XAC_RS04170 | XAC0809 | -1.40459 | hypothetical protein |
| XAC_RS20165 | XAC4004 | -1.02483 | peptidase M20 |
| XAC_RS16845 | XAC3323 | -1.28337 | hypothetical protein |
| XAC_RS20730 | XAC4114 | -1.58286 | ShlB/FhaC/HecB family hemolysin secretion/activation protein |
| XAC_RS06095 | XAC1194 | -1.73384 | type II toxin-antitoxin system death-on-curing family toxin |
| XAC_RS22500 | XACb0056 | -1.47994 | plasmid replication initiator protein |
| XAC_RS21185 | XAC4201 | -1.61171 | 3-dehydroquinate dehydratase |
| XAC_RS19740 | XAC3913 | -1.04834 | deoxyuridine 5'-triphosphate nucleotidohydrolase |
| XAC_RS23040 | XAC0868 | 1.76848 | hypothetical protein |
| XAC_RS17820 | XAC3524 | -1.36941 | glycosyl transferase |
| XAC_RS04990 | XAC0975 | 3.33772 | 50S ribosomal protein L2 |
| XAC_RS02095 | XAC0399 | -1.70843 | hypothetical protein |
| XAC_RS20540 | XAC4077 | -1.53361 | GNAT family N-acetyltransferase |
| XAC_RS10335 | XAC2032 | -1.24068 | molybdopterin molybdenumtransferase MoeA |
| XAC_RS06615 | XAC1295 | 2.83285 | 50S ribosomal protein L19 |
| XAC_RS14990 | XAC2953 | -2.61988 | GlsB/YeaQ/YmgE family stress response membrane protein |
| XAC_RS12890 | XAC2531 | -1.4528 | TonB-dependent receptor |
| XAC_RS05995 | XAC1175 | -1.28278 | NAD(P)H-hydrate dehydratase |
| XAC_RS17340 | XAC3425 | -1.62798 | cytochrome c biogenesis protein CcdA |
| XAC_RS10935 | XAC2152 | -1.42486 | phosphodiesterase |
| XAC_RS01710 | XAC0327 | -1.74004 | efflux RND transporter periplasmic adaptor subunit |
| XAC_RS20465 | - | -1.72842 | DUF3088 domain-containing protein |
| mdcG | XAC0564 | -1.17582 | phosphoribosyl-dephospho-CoA transferase |
| XAC_RS09950 | XAC1955 | -1.81915 | flagellar hook-basal body complex protein FliE |
| XAC_RS18995 | XAC3759 | -1.52589 | ABC transporter ATP-binding protein |
| XAC_RS19255 | XAC3814 | -1.61856 | multidrug resistance protein NorM |
| XAC_RS20935 | XAC4156 | -1.69737 | 4-oxalomesaconate tautomerase |
| XAC_RS23940 | - | -4.11381 | hypothetical protein |
| asnB | XAC1475 | -1.46092 | asparagine synthase (glutamine-hydrolyzing) |
| XAC_RS17585 | XAC3474 | -1.49631 | MFS transporter |
| XAC_RS04275 | XAC0831 | -1.37856 | oxidoreductase |
| XAC_RS00275 | XAC0054 | -1.58225 | NAD(P)-dependent oxidoreductase |
| XAC_RS07130 | XAC1394 | -1.27183 | hypothetical protein |
| XAC_RS23275 | XAC1506 | 2.81845 | hypothetical protein |
| XAC_RS10085 | XAC1983 | 1.27924 | flagellar hook protein FlgE |
| XAC_RS01865 | XAC0352 | -1.81768 | hypothetical protein |
| XAC_RS18080 | - | -1.04059 | hypothetical protein |
| XAC_RS03665 | - | -1.74589 | beta-galactosidase |
| XAC_RS22470 | - | -1.80832 | IS3 family transposase |
| XAC_RS04515 | XAC0881 | -1.71384 | acyl-CoA synthetase |
| XAC_RS04670 | XAC0910 | -1.13294 | N-formylglutamate amidohydrolase |
| XAC_RS10665 | XAC2098 | -1.66703 | non-ribosomal peptide synthase |
| XAC_RS00555 | XAC0108 | -1.9573 | host attachment protein |
| XAC_RS06700 | XAC1311 | -1.59081 | XRE family transcriptional regulator |
| XAC_RS19320 | XAC3827 | -1.30308 | ABC transporter permease |
| XAC_RS12740 | XAC2506 | -1.9709 | hypothetical protein |
| XAC_RS12925 | - | -1.42174 | hypothetical protein |
| XAC_RS17700 | XAC3498 | -1.85816 | TonB-dependent siderophore receptor |
| XAC_RS00790 | XAC0150 | -1.73889 | hypothetical protein |
| XAC_RS14425 | XAC2841 | -1.76377 | TetR/AcrR family transcriptional regulator |
| XAC_RS21360 | - | -1.65415 | hypothetical protein |
| XAC_RS07590 | XAC1483 | -1.29961 | multidrug efflux RND transporter permease subunit |
| XAC_RS20810 | XAC4130 | -1.58854 | DUF4880 domain-containing protein |
| XAC_RS09700 | XAC1904 | 3.20731 | response regulator |
| XAC_RS06205 | XAC1216 | -1.09126 | hypothetical protein |
| XAC_RS03395 | XAC0654 | -1.45709 | sigma-54-dependent Fis family transcriptional regulator |
| XAC_RS04360 | XAC0848 | -1.90964 | ABC transporter permease |
| XAC_RS09165 | XAC1801 | -1.27165 | MFS transporter |
| XAC_RS18020 | XAC3564 | -1.06696 | hypothetical protein |
| XAC_RS13375 | - | -1.79769e+308 | hypothetical protein |
| XAC_RS08075 | XAC1588 | -1.26527 | DUF1684 domain-containing protein |
| XAC_RS04870 | XAC0951 | 2.86875 | 50S ribosomal protein L25 |
| XAC_RS15180 | - | -1.72835 | hypothetical protein |
| XAC_RS23095 | - | -1.77123 | IS3 family transposase |
| XAC_RS08760 | XAC1719 | 1.81046 | enolase |
| XAC_RS14060 | XAC2769 | -1.52257 | TIGR02099 family protein |
| XAC_RS04930 | XAC0963 | 2.86001 | 50S ribosomal protein L10 |
| XAC_RS08615 | XAC1690 | -1.79939 | GNAT family N-acetyltransferase |
| XAC_RS14035 | XAC2764 | 2.50229 | DUF4870 domain-containing protein |
| XAC_RS21405 | XAC4244 | -1.60103 | xylulokinase |
| XAC_RS01685 | XAC0322 | -1.0454 | NAD(P)-dependent oxidoreductase |
| XAC_RS14595 | XAC2875 | -1.13137 | endonuclease V |
| XAC_RS02240 | XAC0428 | -1.24056 | 4-alpha-glucanotransferase |
| XAC_RS05095 | XAC0996 | 2.53305 | DNA-directed RNA polymerase subunit alpha |
| XAC_RS05005 | XAC0978 | 3.20231 | 30S ribosomal protein S3 |
| XAC_RS02750 | XAC0528 | -1.58697 | hypothetical protein |
| XAC_RS03270 | XAC0630 | -1.07855 | pyridoxal phosphate-dependent aminotransferase |
| XAC_RS23620 | - | -1.58676 | hypothetical protein |
| XAC_RS01660 | XAC0317 | -1.3346 | MFS transporter |
| XAC_RS04190 | XAC0813 | 2.68784 | S-adenosylmethionine synthase |
| XAC_RS00855 | - | -1.6008 | C4-dicarboxylate ABC transporter |
| XAC_RS13145 | XAC2581 | -1.57168 | acyltransferase |
| XAC_RS00085 | XAC0016 | -1.60752 | hypothetical protein |
| XAC_RS04970 | XAC0971 | 2.4483 | 30S ribosomal protein S10 |
| XAC_RS19445 | XAC3852 | -1.2458 | hypothetical protein |
| XAC_RS22315 | XACb0016 | -1.83079 | replication protein A |
| XAC_RS15910 | XAC3139 | -1.29781 | 7-carboxy-7-deazaguanine synthase QueE |
| XAC_RS23235 | - | -1.79769e+308 | hypothetical protein |
| XAC_RS16790 | XAC3314 | 4.36109 | DUF239 domain-containing protein |
| uca | XAC4326 | -1.79188 | urea carboxylase |
| XAC_RS18100 | XAC3580 | 1.16546 | mannose-1-phosphate guanylyltransferase/mannose-6-phosphate isomerase |
| XAC_RS08115 | XAC1596 | -1.0052 | 5'-nucleotidase |
| XAC_RS13895 | XAC2736 | 1.55919 | dienelactone hydrolase family protein |
| XAC_RS18615 | XAC3679 | -1.41284 | methyltransferase domain-containing protein |
| XAC_RS05030 | XAC0983 | 2.34348 | 50S ribosomal protein L24 |
| XAC_RS05070 | XAC0991 | 3.21081 | 50S ribosomal protein L15 |
| XAC_RS20250 | XAC4019 | -1.60715 | hypothetical protein |
| XAC_RS11655 | XAC2288 | 1.45156 | IMP dehydrogenase |
| XAC_RS03135 | XAC0602 | -1.05275 | magnesium and cobalt transporter |
| XAC_RS04840 | - | -1.2772 | glutamyl-tRNA reductase |
| XAC_RS17675 | XAC3492 | -1.54899 | flavohemoglobin expression-modulating QEGLA motif protein |
| XAC_RS00635 | - | -1.44861 | hypothetical protein |
| XAC_RS24545 | - | -2.84592 | hypothetical protein |
| XAC_RS18720 | XAC3700 | -1.06574 | ABC transporter ATP-binding protein |
| XAC_RS13835 | XAC2724 | -1.26641 | D-glycerate dehydrogenase |
| cobA | - | -1.62256 | uroporphyrinogen-III C-methyltransferase |
| XAC_RS15670 | XAC3089 | -1.44703 | hypothetical protein |
| XAC_RS03585 | XAC0691 | -1.10145 | 2-oxoglutarate-dependent dioxygenase |
| gspJ | XAC0701 | -1.88181 | type II secretion system protein GspJ |
| XAC_RS03340 | XAC0644 | -1.134 | sensor domain-containing diguanylate cyclase |
| pdhA | XAC0446 | 1.42844 | pyruvate dehydrogenase (acetyl-transferring) E1 component subunit alpha |
| XAC_RS20030 | XAC3974 | -1.43792 | DNA-binding response regulator |
| XAC_RS00345 | XAC0067 | 1.70574 | phage tail protein |
| XAC_RS21370 | XAC4236 | -1.74698 | ring canal kelch |
| XAC_RS17345 | XAC3426 | -1.72976 | flavin monoamine oxidase family protein |
| XAC_RS00630 | XAC0122 | -1.31951 | TldD/PmbA family protein |
| XAC_RS22550 | XACb0069 | -1.08474 | PIN domain nuclease |
| XAC_RS01250 | XAC0240 | -2.46827 | D-alanine--D-alanine ligase |
| XAC_RS21985 | XAC4360 | -1.56827 | glycerate kinase |
| XAC_RS17420 | XAC3441 | -1.8356 | hypothetical protein |
| XAC_RS17250 | XAC3405 | -1.34908 | Xaa-Pro aminopeptidase |
| XAC_RS12355 | - | -1.81687 | Tn3 family transposase |
| XAC_RS18530 | XAC3665 | -1.45045 | hypothetical protein |
| XAC_RS20090 | XAC3988 | -1.61329 | membrane protein |
| XAC_RS20125 | XAC3995 | -1.63579 | efflux RND transporter periplasmic adaptor subunit |
| XAC_RS09495 | - | -1.73772 | hypothetical protein |
| XAC_RS16870 | XAC3328 | -1.47316 | adenylyl-sulfate kinase |
| XAC_RS19725 | XAC3910 | -1.59088 | membrane protein |
| XAC_RS21400 | XAC4243 | -1.43619 | class II aldolase family protein |
| XAC_RS20455 | XAC4061 | -1.77493 | hypothetical protein |
| XAC_RS21885 | XAC4340 | -1.11459 | ABC transporter permease |
| XAC_RS13425 | XAC2639 | -1.47926 | site-specific DNA-methyltransferase |
| XAC_RS01785 | - | -2.11588 | hypothetical protein |
| XAC_RS08525 | XAC1673 | -1.93049 | hypothetical protein |
| XAC_RS00390 | XAC0075 | -1.67713 | ROK family protein |
| XAC_RS04480 | XAC0874 | -1.66374 | hydrolase |
| mdcH | XAC0566 | -1.53399 | malonate decarboxylase subunit epsilon |
| XAC_RS10880 | XAC2141 | -1.39441 | DNA-binding response regulator |
| XAC_RS13875 | - | -1.55424 | hypothetical protein |
| XAC_RS12255 | XAC2410 | -1.16867 | exodeoxyribonuclease VII large subunit |
| XAC_RS22785 | XAC0167 | -1.90391 | hypothetical protein |
| XAC_RS00430 | XAC0083 | -1.20173 | KR domain-containing protein |
| XAC_RS18715 | XAC3699 | -1.16983 | ABC transporter permease |
| XAC_RS10700 | XAC2106 | -1.16577 | aminoacetone oxidase family FAD-binding enzyme |
| tolB | XAC3142 | 1.73081 | protein TolB |
| XAC_RS05015 | XAC0980 | 1.79769e+308 | 50S ribosomal protein L29 |
| XAC_RS14490 | XAC2854 | -1.92254 | two-component system sensor histidine kinase CreC |
| XAC_RS04270 | XAC0830 | -1.68036 | TauD/TfdA family dioxygenase |
| pcaD | XAC0370 | -1.56887 | 3-oxoadipate enol-lactonase |
| XAC_RS14110 | XAC2779 | -1.4517 | DNA polymerase III subunit delta |
| XAC_RS02415 | XAC0463 | -1.41632 | nucleoside-diphosphate sugar epimerase |
| XAC_RS08120 | XAC1597 | -1.43237 | DUF2939 domain-containing protein |
| XAC_RS12725 | XAC2503 | -1.34867 | PTS fructose transporter subunit IIBC |
| XAC_RS12310 | XAC2420 | -1.27242 | hypothetical protein |
| XAC_RS10095 | XAC1985 | -1.17699 | flagellar basal body rod protein FlgC |
| XAC_RS02645 | XAC0508 | -1.194 | LysR family transcriptional regulator |
| XAC_RS07100 | XAC1388 | -1.3782 | membrane protein |
| XAC_RS21085 | XAC4183 | -1.64831 | xylosidase |
| XAC_RS03840 | XAC0742 | 1.38095 | hypothetical protein |
| XAC_RS18525 | XAC3664 | 1.91651 | membrane protein |
| pcaH | XAC0367 | -1.56126 | protocatechuate 3,4-dioxygenase subunit beta |
| XAC_RS04240 | XAC0824 | -1.97225 | hypothetical protein |
| XAC_RS09620 | XAC1891 | 2.72535 | methyl-accepting chemotaxis protein |
| XAC_RS04980 | XAC0973 | 1.99599 | 50S ribosomal protein L4 |
| mutY | XAC2553 | -1.52604 | A/G-specific adenine glycosylase |
| XAC_RS00910 | XAC0175 | -1.37249 | Lrp/AsnC family transcriptional regulator |
| XAC_RS07495 | XAC1466 | 1.28696 | glycine zipper 2TM domain-containing protein |
| XAC_RS16500 | - | -1.09279 | 30S ribosomal protein S6--L-glutamate ligase |
| XAC_RS00185 | XAC0035 | -1.28495 | DNA topoisomerase |
| XAC_RS13085 | XAC2569 | -1.26867 | NAD(P)-dependent oxidoreductase |
| XAC_RS19590 | XAC3882 | -1.75786 | SURF1 family protein |
| XAC_RS16365 | XAC3227 | -1.7641 | Tn3 family resolvase |
| XAC_RS05375 | XAC1051 | -1.09492 | hypothetical protein |
| pgaD | XAC1810 | -1.43279 | poly-beta-1,6-N-acetyl-D-glucosamine biosynthesis protein PgaD |
| XAC_RS22345 | - | -1.2562 | plasmid stabilization protein |
| XAC_RS11280 | XAC2220 | -1.87237 | hypothetical protein |
| XAC_RS22880 | XAC0468 | -1.48978 | hypothetical protein |
| XAC_RS02340 | XAC0447 | -1.75779 | nuclease |
| XAC_RS10900 | XAC2144 | 2.23538 | autotransporter domain-containing protein |
| XAC_RS15985 | XAC3153 | -1.24064 | N-acetyltransferase |
| XAC_RS13880 | XAC2733 | -1.57899 | rhomboid family intramembrane serine protease |
| tsf | XAC1421 | 2.54076 | elongation factor Ts |
| XAC_RS16075 | XAC3171 | -1.53985 | Kef family K(+) transporter |
| XAC_RS07900 | XAC1552 | 1.79769e+308 | protein SlyX |
| XAC_RS06765 | XAC1324 | 2.07928 | DUF4845 domain-containing protein |
| XAC_RS11320 | XAC2227 | -1.6527 | drug/metabolite DMT transporter permease |
| XAC_RS08205 | XAC1612 | -1.7919 | DNA-binding response regulator |
| XAC_RS15380 | XAC3029 | -1.57492 | histidine kinase |
| XAC_RS02190 | XAC0418 | -1.75839 | hypothetical protein |
| XAC_RS09605 | XAC1888 | 1.10758 | chemotaxis response regulator protein-glutamate methylesterase |
| XAC_RS00205 | XAC0039 | -1.78635 | LysR family transcriptional regulator |
| XAC_RS10320 | XAC2029 | -1.84089 | DUF1264 domain-containing protein |
| XAC_RS19015 | - | 2.7358 | hypothetical protein |
| XAC_RS12545 | XAC2467 | -1.36404 | DUF4105 domain-containing protein |
| XAC_RS00245 | XAC0048 | -1.59894 | hypothetical protein |
| XAC_RS05035 | XAC0984 | 3.37898 | 50S ribosomal protein L5 |
| XAC_RS22230 | - | -1.86855 | hypothetical protein |
| gap | XAC3352 | 2.00726 | type I glyceraldehyde-3-phosphate dehydrogenase |
| XAC_RS09025 | XAC1771 | -1.49575 | 9-O-acetylesterase |
| XAC_RS15730 | XAC3101 | 2.53632 | response regulator |
| XAC_RS20855 | XAC4141 | -1.53467 | type VI secretion system baseplate subunit TssG |
| XAC_RS17785 | XAC3518 | -1.76031 | UDP-forming cellulose synthase catalytic subunit |
| XAC_RS06295 | - | -1.11766 | peptidase |
| XAC_RS02395 | XAC0459 | -2.0804 | Na+/H+ antiporter subunit E |
| XAC_RS14470 | XAC2850 | -1.43114 | N-acetyltransferase |
| XAC_RS08770 | XAC1721 | -1.06765 | 2-C-methyl-D-erythritol 4-phosphate cytidylyltransferase |
| XAC_RS24185 | - | -1.79769e+308 | lactoylglutathione lyase |
| XAC_RS00230 | XAC0045 | -1.57199 | ligase |
| XAC_RS04405 | XAC0857 | -1.75086 | ABC transporter permease |
| XAC_RS21935 | XAC4350 | -1.66905 | LysR family transcriptional regulator |
| XAC_RS15585 | XAC3071 | 2.30378 | TonB-dependent receptor |
| XAC_RS15200 | XAC2995 | -1.57575 | tryptophan 7-halogenase |
| XAC_RS21450 | - | -1.96452 | 1,4-beta-xylanase |
| XAC_RS14325 | XAC2821 | -1.56038 | hypothetical protein |
| XAC_RS01480 | XAC0283 | -1.35338 | alpha/beta hydrolase |
| XAC_RS17375 | XAC3432 | -1.08038 | serine/threonine protein kinase |
| XAC_RS01925 | XAC0364 | -1.66267 | CoA transferase subunit A |
| XAC_RS07525 | XAC1472 | -1.04957 | acyl-CoA dehydrogenase |
| XAC_RS01190 | XAC0228 | -1.34898 | hypothetical protein |
| XAC_RS02525 | XAC0485 | -1.10237 | QacE family quaternary ammonium compound efflux SMR transporter |
| XAC_RS16385 | - | -1.03102 | hypothetical protein |
| XAC_RS05130 | XAC1002 | -1.34391 | amidase |
| XAC_RS11265 | - | -1.79769e+308 | hypothetical protein |
| gspI | XAC0700 | -1.9537 | type II secretion system protein GspI |
| XAC_RS13090 | XAC2570 | -1.7069 | MBL fold metallo-hydrolase |
| XAC_RS10495 | XAC2064 | -1.18874 | efflux RND transporter periplasmic adaptor subunit |
| XAC_RS06285 | XAC1231 | -1.5097 | DUF423 domain-containing protein |
| XAC_RS06610 | XAC1294 | -1.21328 | tRNA (guanosine(37)-N1)-methyltransferase TrmD |
| XAC_RS14095 | XAC2776 | -1.79793 | DUF839 domain-containing protein |
| XAC_RS13240 | - | -4.52689 | hypothetical protein |
| XAC_RS20950 | XAC4159 | 1.78442 | 50S ribosomal protein L28 |
| XAC_RS04855 | XAC0948 | -1.25513 | 4-diphosphocytidyl-2C-methyl-D-erythritol kinase |
| XAC_RS20120 | XAC3994 | -1.41435 | sensor histidine kinase |
| XAC_RS06030 | XAC1181 | -1.42618 | alpha-ketoglutarate-dependent dioxygenase AlkB |
| XAC_RS01075 | XAC0205 | -2.24007 | P-II family nitrogen regulator |
| glyA | XAC0743 | 1.08406 | serine hydroxymethyltransferase |
| XAC_RS20330 | XAC4036 | -1.6726 | iron-uptake factor |
| XAC_RS06635 | XAC1299 | -1.7745 | glutathione S-transferase family protein |
| XAC_RS13050 | XAC2562 | -1.27992 | hypothetical protein |
| XAC_RS16220 | XAC3200 | -1.57462 | LLM class flavin-dependent oxidoreductase |
| XAC_RS16155 | XAC3187 | -1.71529 | bifunctional adenosylcobinamide kinase/adenosylcobinamide-phosphate guanylyltransferase |
| XAC_RS13550 | XAC2669 | 2.19278 | prepilin-type N-terminal cleavage/methylation domain-containing protein |
| XAC_RS17880 | XAC3537 | -1.38531 | general secretion pathway protein GspL |
| XAC_RS24170 | - | -1.72365 | Oar protein |
| XAC_RS00915 | XAC0176 | -1.6761 | TonB-dependent siderophore receptor |
| XAC_RS17140 | XAC3383 | 2.00382 | fimbrial protein |
| XAC_RS05900 | XAC1157 | -1.43442 | hypothetical protein |
| gpmA | XAC2874 | 1.96851 | 2,3-bisphosphoglycerate-dependent phosphoglycerate mutase |
| XAC_RS08780 | XAC1723 | -1.14597 | tRNA pseudouridine(13) synthase TruD |
| XAC_RS11865 | XAC2330 | -1.82093 | cytochrome c-type biogenesis protein CcmH |
| XAC_RS02400 | XAC0460 | -1.34552 | monovalent cation/H+ antiporter subunit D |
| XAC_RS04395 | XAC0855 | -1.63316 | FMN-dependent monooxygenase |
| XAC_RS02135 | XAC0407 | -1.84169 | HrpB1 family type III secretion system apparatus protein |
| XAC_RS10845 | XAC2134 | -1.51853 | DedA family protein |
| XAC_RS06845 | XAC1340 | -1.40151 | DNA ligase-associated DEXH box helicase |
| epmB | XAC2381 | -1.14646 | EF-P beta-lysylation protein EpmB |
| XAC_RS20040 | XAC3976 | -1.37872 | hypothetical protein |
| XAC_RS13870 | XAC2731 | -1.28159 | GDP-mannose pyrophosphatase NudK |
| XAC_RS00905 | XAC0174 | 1.76641 | phenylalanine 4-monooxygenase |
| XAC_RS15645 | XAC3083 | -1.24878 | hypothetical protein |
| XAC_RS11380 | XAC2239 | -1.37066 | hypothetical protein |
| XAC_RS11165 | XAC2196 | -1.79377 | relaxase |
| XAC_RS20410 | XAC4052 | 2.19341 | energy transducer TonB |
| XAC_RS03675 | XAC0710 | -1.60079 | DUF1624 domain-containing protein |
| XAC_RS16445 | XAC3241 | 5.90643 | prepilin-type cleavage/methylation domain-containing protein |
| XAC_RS09695 | XAC1903 | 2.59683 | chemotaxis protein CheA |
| rsmI | XAC0766 | -1.73237 | 16S rRNA (cytidine(1402)-2'-O)-methyltransferase |
| mutS | XAC1303 | -1.29324 | DNA mismatch repair protein MutS |
| XAC_RS07075 | - | -1.6582 | hypothetical protein |
| XAC_RS22250 | XACb0002 | -1.01072 | hypothetical protein |
| XAC_RS03080 | - | -2.3835 | hypothetical protein |
| XAC_RS14005 | XAC2758 | -1.10266 | dicarboxylate/amino acid:cation symporter |
| XAC_RS24690 | - | -1.36519 | hypothetical protein |
| XAC_RS03820 | XAC0738 | -1.40133 | PhzF family phenazine biosynthesis protein |
| XAC_RS05810 | XAC1137 | 1.26353 | methylisocitrate lyase |
| XAC_RS01435 | XAC0276 | -1.75351 | GTP-binding protein |
| XAC_RS01565 | XAC0299 | -1.97237 | allantoinase PuuE |
| XAC_RS20490 | - | -1.80245 | 3-deoxy-D-manno-octulosonic acid transferase |
| XAC_RS21445 | XAC4251 | -1.29778 | uronate isomerase |
| XAC_RS08000 | XAC1574 | -1.62111 | phosphate ABC transporter ATP-binding protein PstB |
| XAC_RS04985 | XAC0974 | 1.77584 | 50S ribosomal protein L23 |
| XAC_RS09175 | XAC1803 | -1.30945 | EamA/RhaT family transporter |
| pqqD | XAC3116 | -2.07603 | pyrroloquinoline quinone biosynthesis protein PqqD |
| XAC_RS19300 | XAC3823 | -1.71699 | trimeric intracellular cation channel family protein |
| XAC_RS19185 | XAC3800 | -1.04225 | methionyl-tRNA formyltransferase |
| XAC_RS16170 | XAC3190 | -1.39032 | cobalamin biosynthesis protein |
| XAC_RS00975 | XAC0187 | -1.11242 | HipA protein |
| XAC_RS20655 | XAC4099 | -1.2821 | 1-acyl-sn-glycerol-3-phosphate acyltransferase |
| XAC_RS12400 | XAC2439 | -1.23815 | plasmid pRiA4b ORF-3 family protein |
| XAC_RS17635 | XAC3484 | -1.7432 | porin |
| XAC_RS19835 | XAC3930 | 1.69078 | hypothetical protein |
| XAC_RS00695 | - | -2.98548 | hypothetical protein |
| XAC_RS12825 | XAC2520 | -1.85009 | TonB-dependent receptor |
| XAC_RS01500 | XAC0287 | -1.27897 | NADP-dependent oxidoreductase |
| XAC_RS16055 | XAC3167 | -1.31023 | VOC family protein |
| XAC_RS07520 | XAC1471 | 2.47874 | glycine zipper 2TM domain-containing protein |
| XAC_RS21030 | XAC4174 | -1.59055 | peptidyl-prolyl cis-trans isomerase |
| XAC_RS06085 | XAC1192 | -1.21651 | cupin domain-containing protein |
| XAC_RS21145 | XAC4193 | -1.58173 | hybrid sensor histidine kinase/response regulator |
| XAC_RS18895 | - | -1.72067 | KR domain-containing protein |
| XAC_RS17920 | XAC3545 | -1.75324 | protease |
| XAC_RS04125 | XAC0800 | -1.21213 | DUF3228 domain-containing protein |
| XAC_RS23965 | XAC3058 | 4.2485 | hypothetical protein |
| XAC_RS22410 | XACb0038 | -1.01528 | type VI secretion protein |
| XAC_RS04810 | - | -2.60892 | acylphosphatase |
| XAC_RS17625 | XAC3482 | -1.36017 | sensor histidine kinase |
| XAC_RS05935 | XAC1163 | -1.00728 | hypothetical protein |
| XAC_RS23500 | XAC1923 | -1.4188 | hypothetical protein |
| XAC_RS10990 | - | -1.81765 | amidohydrolase |
| uraD | XAC0297 | -1.52373 | OHCU decarboxylase |
| XAC_RS07565 | XAC1479 | 1.91718 | OmpA family lipoprotein |
| XAC_RS04615 | XAC0898 | -1.77362 | DNA-binding response regulator |
| XAC_RS00505 | XAC0099 | -1.58943 | hypothetical protein |
| XAC_RS13925 | XAC2742 | -1.68233 | TonB-dependent receptor |
| XAC_RS02835 | XAC0544 | -1.41026 | membrane protein |
| XAC_RS17435 | XAC3444 | 3.57298 | TonB-dependent receptor |
| XAC_RS12795 | XAC2515 | -1.32041 | Lrp/AsnC family transcriptional regulator |
| XAC_RS08060 | XAC1585 | 3.37751 | FKBP-type peptidyl-prolyl cis-trans isomerase |
| XAC_RS10040 | XAC1974 | 2.21335 | flagellar protein |
| XAC_RS22325 | XACb0018 | -1.54215 | hypothetical protein |
| XAC_RS17275 | XAC3410 | -1.01883 | EVE domain-containing protein |
| XAC_RS21375 | XAC4237 | -1.53623 | TetR/AcrR family transcriptional regulator |
| cydD | XAC2335 | -1.08432 | thiol reductant ABC exporter subunit CydD |
| XAC_RS21605 | - | -1.28044 | DUF4380 domain-containing protein |
| XAC_RS18780 | XAC3714 | -1.0785 | DUF2236 domain-containing protein |
| XAC_RS01405 | XAC0270 | -1.5118 | PepSY domain-containing protein |
| XAC_RS18650 | XAC3686 | 1.25658 | DUF3016 domain-containing protein |
| aroA | XAC1650 | -1.19156 | 3-phosphoshikimate 1-carboxyvinyltransferase |
| XAC_RS11385 | XAC2240 | -1.79769e+308 | hypothetical protein |
| XAC_RS01285 | XAC0247 | -1.04147 | acyltransferase |
| XAC_RS18050 | XAC3570 | -1.22381 | ABC transporter permease |
| hutC | XAC1640 | -1.13066 | histidine utilization repressor |
| potG | XAC2472 | -1.3472 | polyamine ABC transporter ATP-binding protein |
| XAC_RS18770 | XAC3711 | -1.5231 | glycosyl transferase family 2 |
| XAC_RS01765 | XAC0338 | -1.5062 | porin |
| XAC_RS16405 | XAC3235 | 2.47822 | succinate--CoA ligase subunit alpha |
| XAC_RS15335 | XAC3020 | -2.38357 | hypothetical protein |
| XAC_RS09935 | XAC1952 | -1.10952 | flagellar assembly protein FliH |
| XAC_RS04925 | XAC0962 | 2.10408 | 50S ribosomal protein L1 |
| XAC_RS12050 | XAC2368 | -1.44537 | MBL fold metallo-hydrolase |
| XAC_RS17020 | XAC3359 | -1.38912 | molybdate ABC transporter permease subunit |
| XAC_RS21230 | - | -1.7852 | colicin V production protein |
| XAC_RS06685 | XAC1308 | -1.44898 | DUF4982 domain-containing protein |
| XAC_RS05750 | XAC1124 | -1.71237 | MEKHLA domain-containing protein |
| XAC_RS23445 | XAC1817 | -1.02145 | hypothetical protein |
| XAC_RS23165 | - | -2.37994 | hypothetical protein |
| XAC_RS17885 | XAC3538 | -1.74152 | general secretion pathway protein GspK |
| XAC_RS03020 | XAC0581 | -1.41526 | AraC family transcriptional regulator |
| XAC_RS20800 | XAC4128 | -1.70013 | DNA-directed RNA polymerase sigma-70 factor |
| XAC_RS03390 | XAC0653 | -1.74362 | TonB-dependent receptor |
| XAC_RS22850 | - | -1.34134 | hypothetical protein |
| XAC_RS03210 | XAC0616 | -1.9136 | hypothetical protein |
| XAC_RS08980 | XAC1763 | -1.14403 | MFS transporter |
| XAC_RS08095 | XAC1592 | -1.69495 | N-acetyltransferase |
| XAC_RS21785 | XAC4320 | -1.69361 | pseudouridine synthase |
| XAC_RS05090 | XAC0995 | 1.74842 | 30S ribosomal protein S4 |
| XAC_RS07935 | XAC1559 | -1.23481 | methionine synthase |
| XAC_RS13125 | XAC2577 | -1.48744 | lipopolysaccharide biosynthesis protein |
| XAC_RS14000 | XAC2757 | -1.68015 | class I SAM-dependent methyltransferase |
| XAC_RS11220 | XAC2208 | -1.69103 | hypothetical protein |
| XAC_RS01880 | XAC0355 | -1.39498 | AraC family transcriptional regulator |
| XAC_RS13940 | XAC2745 | 3.00743 | peptidase |
| XAC_RS08935 | XAC1754 | -1.39776 | DUF1275 domain-containing protein |
| XAC_RS02830 | XAC0543 | -1.86581 | hypothetical protein |
| cyoB | XAC1259 | 2.58796 | cytochrome o ubiquinol oxidase subunit I |
| XAC_RS05245 | XAC1026 | -1.79769e+308 | hypothetical protein |
| XAC_RS01570 | XAC0300 | -1.57469 | alanine--glyoxylate aminotransferase family protein |
| XAC_RS16375 | XAC3229 | -1.59362 | transposase |
| XAC_RS10360 | XAC2037 | -1.34654 | transcriptional regulator MntR |
| XAC_RS23585 | - | -1.27123 | uroporphyrin-III C-methyltransferase |
| XAC_RS17120 | XAC3378 | -1.5559 | DUF58 domain-containing protein |
| XAC_RS18575 | - | -1.03907 | hypothetical protein |
| XAC_RS04580 | XAC0892 | -1.62335 | EamA/RhaT family transporter |
| XAC_RS14860 | XAC2927 | 4.51529 | DNA-binding protein |
| XAC_RS01875 | XAC0354 | -1.33674 | benzaldehyde dehydrogenase |
| XAC_RS04355 | XAC0847 | -1.41299 | aliphatic sulfonate ABC transporter ATP-binding protein 1 |
| XAC_RS05440 | - | 1.42007 | hypothetical protein |
| XAC_RS03265 | XAC0629 | -1.63586 | ATPase AAA |
| XAC_RS17330 | XAC3423 | -1.59805 | pyridoxal phosphate-dependent aminotransferase |
| XAC_RS05840 | XAC1144 | -1.53196 | inosine-uridine preferring nucleoside hydrolase |
| XAC_RS12040 | XAC2366 | -1.34497 | ethanolamine ammonia-lyase light chain |
| XAC_RS15005 | XAC2956 | -1.48024 | DnaA regulatory inactivator Hda |
| XAC_RS06115 | XAC1198 | -1.46803 | DNA polymerase Y family protein |
| ntrC | XAC0208 | -1.56005 | nitrogen regulation protein NR(I) |
| XAC_RS17045 | XAC3364 | -1.13668 | acetyl-CoA hydrolase |
| XAC_RS21385 | XAC4239 | -1.45281 | MFS transporter |
| XAC_RS18830 | XAC3726 | -1.58016 | Mn-containing catalase |
| XAC_RS10575 | XAC2080 | -1.3073 | hypothetical protein |
| XAC_RS13515 | XAC2659 | -1.79864 | hypothetical protein |
| XAC_RS19060 | XAC3773 | -1.57419 | FMN-dependent NADH-azoreductase 2 |
| pgaA | XAC1813 | -1.74907 | poly-beta-1,6 N-acetyl-D-glucosamine export porin PgaA |
| XAC_RS18545 | - | -1.49983 | hypothetical protein |
| XAC_RS16970 | - | -1.39478 | hypothetical protein |
| XAC_RS05270 | XAC1031 | -1.52518 | colicin V biosynthesis protein |
| XAC_RS11630 | XAC2285 | -1.89114 | phosphoadenosine phosphosulfate reductase |
| XAC_RS20980 | XAC4165 | -1.07773 | ribosomal RNA small subunit methyltransferase G |
| XAC_RS14965 | XAC2948 | -1.84955 | sulfite reductase |
| XAC_RS16780 | XAC3312 | -1.2626 | beta-galactosidase |
| XAC_RS00440 | XAC0085 | -1.81005 | hypothetical protein |
| XAC_RS24125 | - | -2.05591 | hypothetical protein |
| XAC_RS01265 | XAC0243 | -1.49393 | cupin domain-containing protein |
| XAC_RS18665 | XAC3689 | -1.29498 | AsnC family transcriptional regulator |
| XAC_RS21700 | XAC4303 | -1.56743 | MarR family transcriptional regulator |
| XAC_RS14960 | XAC2947 | -1.40478 | FAD:protein FMN transferase |
| thiL | XAC0752 | -1.15348 | thiamine-phosphate kinase |
| XAC_RS19215 | XAC3806 | -1.00214 | DUF4339 domain-containing protein |
| XAC_RS17890 | XAC3539 | -1.58706 | general secretion pathway protein GspJ |
| XAC_RS20760 | XAC4120 | -1.79114 | hypothetical protein |
| XAC_RS05975 | - | -1.83589 | serine/threonine protein kinase |
| XAC_RS16470 | XAC3246 | -1.31849 | type I addiction module toxin, SymE family |
| XAC_RS16715 | XAC3300 | 1.67142 | autotransporter domain-containing esterase |
| XAC_RS09835 | XAC1932 | 3.5348 | chemotaxis protein CheY |
| XAC_RS03085 | XAC0593 | -1.11177 | GGDEF domain-containing protein |
| XAC_RS04145 | XAC0804 | 1.76915 | adenosylhomocysteinase |
| XAC_RS06620 | - | -1.09472 | MATE family efflux transporter |
| XAC_RS14435 | XAC2843 | -1.61878 | multidrug efflux RND transporter permease subunit |
| XAC_RS11815 | XAC2320 | -1.02581 | glutamine cyclotransferase |
| XAC_RS09805 | XAC1926 | -1.48509 | hypothetical protein |
| XAC_RS17645 | XAC3486 | -1.43372 | KR domain-containing protein |
| XAC_RS21350 | XAC4232 | -1.77168 | mannitol dehydrogenase family protein |
| XAC_RS04140 | XAC0803 | -1.72353 | class I SAM-dependent methyltransferase |
| XAC_RS09065 | XAC1780 | -1.728 | N-acetylmuramoyl-L-alanine amidase |
| rfbB | XAC3585 | 1.30926 | dTDP-glucose 4,6-dehydratase |
| XAC_RS07155 | - | -1.01576 | hypothetical protein |
| XAC_RS06465 | XAC1267 | -1.2993 | molecular chaperone HtpG |
| XAC_RS20845 | XAC4137 | -1.75274 | IS4 family transposase ISXac1 |
| XAC_RS10405 | XAC2046 | -1.12446 | CDP-diacylglycerol--serine O-phosphatidyltransferase |
| XAC_RS00435 | XAC0084 | -1.19738 | LysR family transcriptional regulator |
| XAC_RS20230 | XAC4016 | -1.57375 | SPOR domain-containing protein |
| XAC_RS01580 | XAC0302 | -1.5646 | LysR family transcriptional regulator |
| XAC_RS12170 | - | -1.09827 | alpha/beta hydrolase |
| XAC_RS21100 | XAC4185 | -1.59727 | amidohydrolase |
| XAC_RS17095 | XAC3373 | -1.38553 | dicarboxylate/amino acid:cation symporter |
| XAC_RS09500 | XAC1868 | -1.92541 | hypothetical protein |
| XAC_RS06555 | XAC1285 | -1.06752 | glycoside hydrolase family 16 protein |
| XAC_RS12810 | XAC2517 | -1.4557 | hypothetical protein |
| XAC_RS10490 | XAC2063 | 1.09176 | cytochrome c |
| XAC_RS21650 | XAC4294 | -1.35514 | heme oxygenase |
| XAC_RS00030 | XAC0005 | -1.63911 | CPBP family intramembrane metalloprotease |
| gcvH | XAC3060 | 4.08788 | glycine cleavage system protein H |
| XAC_RS23760 | XAC2607 | -1.49104 | hypothetical protein |
| XAC_RS02410 | XAC0462 | -1.50733 | monovalent cation/H+ antiporter subunit A |
| XAC_RS22285 | - | -1.2648 | Tn3 family resolvase |
| XAC_RS12955 | XAC2544 | 1.11501 | DUF885 domain-containing protein |
| XAC_RS06405 | XAC1255 | -1.00279 | lipoprotein signal peptidase |
| XAC_RS10620 | XAC2089 | -1.28025 | 3-deoxy-manno-octulosonate cytidylyltransferase |
| XAC_RS04730 | XAC0922 | -1.58349 | RNA polymerase sigma factor |
| XAC_RS04495 | XAC0877 | -1.57855 | GntR family transcriptional regulator |
| XAC_RS10565 | XAC2078 | 2.06212 | succinate dehydrogenase iron-sulfur subunit |
| XAC_RS08100 | XAC1593 | -1.51148 | ABC transporter ATP-binding protein |
| XAC_RS01600 | XAC0306 | -1.37658 | AtzE family amidohydrolase |
| XAC_RS06930 | XAC1356 | -1.49331 | mechanosensitive ion channel family protein |
| XAC_RS14295 | XAC2815 | -1.74528 | hypothetical protein |
| XAC_RS23490 | - | -1.62886 | IS3 family transposase |
| XAC_RS12710 | XAC2500 | -1.25083 | LacI family DNA-binding transcriptional regulator |
| XAC_RS16850 | XAC3324 | -1.23833 | hypothetical protein |
| XAC_RS01515 | XAC0290 | -1.26913 | ribosomal large subunit pseudouridine synthase E |
| XAC_RS19450 | XAC3853 | -1.292 | hypothetical protein |
| XAC_RS00115 | XAC0022 | -1.40434 | 3-phosphoglycerate dehydrogenase |
| XAC_RS07980 | XAC1570 | -1.76123 | GGDEF domain-containing protein |
| XAC_RS01195 | XAC0229 | -1.76119 | MFS transporter |
| XAC_RS16785 | XAC3313 | -1.0211 | alpha-glucosidase |
| XAC_RS18825 | XAC3725 | -1.81529 | ferritin-like domain-containing protein |
| XAC_RS11185 | XAC2201 | -1.86914 | HlyD family type I secretion periplasmic adaptor subunit |
| XAC_RS16030 | XAC3163 | -1.70918 | LysR family transcriptional regulator |
| XAC_RS11580 | XAC2275 | -1.52729 | hypothetical protein |
| XAC_RS22290 | XACb0010 | -1.47254 | transposase |
| XAC_RS09040 | XAC1774 | -1.48521 | alpha/beta hydrolase |
| XAC_RS17835 | XAC3527 | -1.74773 | hypothetical protein |
| XAC_RS20115 | XAC3993 | -1.79769e+308 | DNA-binding response regulator |
| XAC_RS23020 | - | -1.79769e+308 | hypothetical protein |
| XAC_RS16380 | XAC3230 | -1.68284 | hypothetical protein |
| XAC_RS06385 | XAC1251 | 4.4252 | 30S ribosomal protein S20 |
| XAC_RS10365 | XAC2038 | -1.61674 | tRNA adenosine(34) deaminase TadA |
| XAC_RS23990 | - | -1.88811 | hypothetical protein |
| XAC_RS14970 | XAC2949 | -1.41445 | calcium-binding protein |
| XAC_RS04625 | XAC0900 | 1.6 | peptide-methionine (S)-S-oxide reductase |
| XAC_RS11925 | XAC2342 | -1.03119 | gamma-glutamyl-phosphate reductase |
| XAC_RS19560 | XAC3876 | -1.34365 | DNA primase |
| XAC_RS12895 | XAC2532 | -1.66073 | S9 family peptidase |
| XAC_RS18840 | XAC3728 | -1.61373 | hypothetical protein |
| XAC_RS23450 | - | -1.49343 | IS3 family transposase |
| XAC_RS02000 | XAC0379 | -1.28258 | serine/threonine dehydratase |
| XAC_RS18560 | XAC3669 | -1.17431 | methionine import ATP-binding protein MetN |
| XAC_RS05190 | XAC1015 | -1.91981 | hypothetical protein |
| XAC_RS01640 | XAC0313 | -1.38065 | preprotein translocase subunit TatD |
| XAC_RS13080 | XAC2568 | -1.4344 | hypothetical protein |
| XAC_RS12080 | XAC2375 | -1.09741 | phytoene synthase |
| XAC_RS14770 | XAC2910 | -1.4224 | hypothetical protein |
| vasA | XAC4142 | -1.66419 | type VI secretion system baseplate subunit TssF |
| XAC_RS17925 | XAC3546 | -1.68247 | membrane protein |
| XAC_RS14940 | XAC2943 | -1.34298 | sel1 repeat family protein |
| XAC_RS14075 | XAC2772 | 2.18857 | SIMPL domain-containing protein |
| XAC_RS07285 | XAC1424 | -1.6482 | spore coat protein U |
| XAC_RS17640 | XAC3485 | -1.90039 | citrate transporter |
| XAC_RS23215 | - | -1.64926 | hypothetical protein |
| XAC_RS18485 | XAC3656 | -1.52747 | hypothetical protein |
| XAC_RS05415 | XAC1057 | -1.55084 | hypothetical protein |
| XAC_RS09640 | XAC1894 | 1.57042 | methyl-accepting chemotaxis protein |
| XAC_RS01105 | XAC0211 | -1.81223 | glyoxalase |
| XAC_RS16225 | XAC3201 | -1.74436 | TonB-dependent receptor |
| XAC_RS06015 | XAC1179 | -1.0402 | glycoside hydrolase family 92 protein |
| XAC_RS01820 | XAC0345 | -1.46994 | dihydroxy-acid dehydratase |
| XAC_RS01970 | XAC0373 | -1.49438 | HTH domain-containing protein |
| XAC_RS04185 | XAC0812 | -1.91658 | histidine-type phosphatase |
| XAC_RS05085 | XAC0994 | 3.78472 | 30S ribosomal protein S11 |
| XAC_RS04255 | XAC0827 | -1.72071 | ABC transporter permease |
| XAC_RS07735 | XAC1520 | -1.17163 | heat-inducible transcriptional repressor HrcA |
| XAC_RS22715 | - | -2.24526 | hypothetical protein |
| XAC_RS12505 | XAC2459 | -1.19213 | transcriptional regulator |
| XAC_RS23715 | - | -1.66742 | recombinase |
| XAC_RS24340 | XAC3784 | -2.09012 | hypothetical protein |
| XAC_RS16240 | XAC3204 | -1.62296 | DUF3014 domain-containing protein |
| XAC_RS07450 | XAC1457 | -1.34259 | glutathione peroxidase |
| XAC_RS10210 | XAC2008 | 1.95884 | outer membrane lipoprotein carrier protein LolA |
| XAC_RS00755 | XAC0145 | -1.87175 | hypothetical protein |
| XAC_RS21910 | XAC4345 | -1.12199 | nuclear transport factor 2 family protein |
| XAC_RS12320 | - | -1.81084 | IS5/IS1182 family transposase |
| XAC_RS05485 | XAC1072 | -1.58932 | phage-related DNA-directed RNA polymerase |
| XAC_RS16310 | XAC3217 | -1.05906 | ribosomal large subunit pseudouridine synthase D |
| XAC_RS18000 | XAC3560 | -1.47831 | TonB-dependent receptor |
| XAC_RS02130 | XAC0406 | -1.97242 | EscU/YscU/HrcU family type III secretion system export apparatus switch protein |
| XAC_RS16960 | XAC3346 | -1.28072 | haloacid dehalogenase |
| XAC_RS00655 | - | -2.95137 | hypothetical protein |
| XAC_RS01495 | XAC0286 | -1.96584 | hypothetical protein |
| XAC_RS00405 | XAC0078 | -1.31611 | DUF1998 domain-containing protein |
| XAC_RS19335 | XAC3830 | 1.78413 | thioredoxin TrxA |
| XAC_RS04700 | XAC0916 | -1.49373 | alpha/beta hydrolase |
| XAC_RS08490 | XAC1668 | -1.60277 | transcriptional regulator |
| XAC_RS04195 | XAC0814 | -1.35924 | metal-dependent hydrolase |
| XAC_RS03710 | XAC0717 | -1.48437 | multifunctional CCA tRNA nucleotidyl transferase/2'3'-cyclic phosphodiesterase/2'nucleotidase/phosphatase |
| gspE | XAC3544 | -1.84909 | type II secretion system protein GspE |
| XAC_RS17515 | XAC3460 | -1.13951 | GGDEF domain-containing protein |
| XAC_RS07580 | XAC1481 | -1.44037 | NAD(P)-dependent oxidoreductase |
| XAC_RS19680 | XAC3901 | -1.42162 | MFS transporter |
| XAC_RS24555 | XAC4198 | -2.12549 | hypothetical protein |
| XAC_RS10110 | XAC1988 | -1.05189 | flagella basal body P-ring formation protein FlgA |
| XAC_RS21990 | XAC4361 | -1.74763 | MFS transporter |
| XAC_RS15655 | XAC3085 | -1.89635 | hypothetical protein |
| XAC_RS19575 | XAC3879 | -1.54925 | protoheme IX farnesyltransferase |
| XAC_RS16865 | XAC3327 | -1.08791 | MexH family multidrug efflux RND transporter periplasmic adaptor subunit |
| XAC_RS08600 | XAC1687 | -1.85906 | WxcM-like domain-containing protein |
| XAC_RS17105 | XAC3375 | -1.45866 | VWA domain-containing protein |
| XAC_RS05020 | XAC0981 | 3.6507 | 30S ribosomal protein S17 |
| XAC_RS11350 | XAC2233 | -1.28296 | short chain dehydrogenase |
| XAC_RS22065 | XACa0001 | -1.42371 | hypothetical protein |
| XAC_RS06185 | XAC1212 | -1.0756 | aldo/keto reductase |
| XAC_RS08835 | XAC1734 | -1.34832 | tRNA (adenosine(37)-N6)-dimethylallyltransferase MiaA |
| XAC_RS09305 | XAC1830 | -1.18061 | histidinol-phosphate aminotransferase |
| XAC_RS08645 | XAC1696 | -1.52046 | class I SAM-dependent methyltransferase |
| XAC_RS02845 | XAC0546 | -1.5606 | hypothetical protein |
| XAC_RS00700 | XAC0134 | -1.58497 | serine hydrolase |
| XAC_RS04485 | XAC0875 | -1.1679 | nuclear transport factor 2 family protein |
| XAC_RS02685 | XAC0515 | -1.51604 | hypothetical protein |
| XAC_RS23080 | - | -1.71864 | hypothetical protein |
| XAC_RS02470 | XAC0474 | -1.06355 | N-acetyltransferase |
| XAC_RS00515 | - | -1.48317 | HDOD domain-containing protein |
| XAC_RS14930 | XAC2941 | -1.30622 | TonB-dependent siderophore receptor |
| XAC_RS21390 | XAC4240 | -1.45565 | hypothetical protein |
| XAC_RS00875 | XAC0168 | -1.1495 | 5-keto-4-deoxyuronate isomerase |
| XAC_RS12675 | XAC2492 | -1.21584 | hybrid sensor histidine kinase/response regulator |
| XAC_RS20925 | XAC4154 | -1.4677 | LysR family transcriptional regulator |
| XAC_RS15795 | XAC3115 | -1.07951 | pyrroloquinoline-quinone synthase |
| XAC_RS19825 | XAC3928 | -1.23355 | beta-N-acetylglucosaminidase |
| XAC_RS13105 | XAC2573 | -1.12386 | hypothetical protein |
| pbpC | XAC1148 | -1.5574 | penicillin-binding protein 1C |
| XAC_RS21105 | XAC4186 | -1.18399 | oxidoreductase |
| XAC_RS17400 | XAC3437 | 2.10944 | adenylate kinase |
| XAC_RS09760 | XAC1915 | -1.00702 | hypothetical protein |
| XAC_RS22820 | - | -3.40683 | hypothetical protein |
| XAC_RS22040 | XAC4371 | -1.64658 | tetratricopeptide repeat protein |
| XAC_RS06800 | XAC1331 | -1.38785 | DsbA family oxidoreductase |
| XAC_RS04460 | XAC0870 | -1.49818 | ATP:cob(I)alamin adenosyltransferase |
| XAC_RS14635 | XAC2883 | -1.02706 | HAD family hydrolase |
| XAC_RS10885 | XAC2142 | -1.72958 | two-component system sensor protein |
| accB | XAC0532 | 1.56077 | acetyl-CoA carboxylase biotin carboxyl carrier protein |
| XAC_RS03650 | XAC0704 | -1.37852 | type II secretion system protein M |
| XAC_RS00740 | XAC0142 | -1.79769e+308 | hypothetical protein |
| mdcA | XAC0560 | -1.62602 | malonate decarboxylase subunit alpha |
| XAC_RS01235 | XAC0237 | -1.32823 | peptide synthase |
| XAC_RS16255 | XAC3208 | -1.29479 | hypothetical protein |
| XAC_RS22495 | XACb0054 | -1.1666 | ParA family protein |
| XAC_RS06055 | XAC1186 | -1.03688 | excinuclease ABC subunit UvrA |
| XAC_RS13195 | XAC2591 | 4.44136 | 50S ribosomal protein L20 |
| XAC_RS06440 | XAC1262 | 1.97063 | peptidase M61 |
| XAC_RS15165 | - | -1.79769e+308 | hypothetical protein |
| XAC_RS05310 | XAC1038 | -1.67571 | glycosyltransferase family 1 protein |
| XAC_RS22210 | XACa0036 | -1.06405 | PIN domain nuclease |
| XAC_RS17840 | XAC3528 | -1.49516 | hypothetical protein |
| XAC_RS00765 | XAC0146 | -1.133 | hypothetical protein |
| XAC_RS15640 | XAC3082 | -1.24909 | metal-independent alpha-mannosidase |
| XAC_RS17165 | XAC3388 | 2.56168 | citrate synthase |
| XAC_RS06840 | XAC1339 | -1.80574 | phosphoesterase |
| XAC_RS20020 | XAC3972 | 1.06002 | DUF4142 domain-containing protein |
| XAC_RS17350 | XAC3427 | -1.81393 | TonB-dependent receptor |
| XAC_RS20140 | XAC3998 | -1.52754 | ABC transporter permease |
| XAC_RS13710 | XAC2702 | 1.01554 | NADH-quinone oxidoreductase subunit C |
| XAC_RS04950 | XAC0967 | 3.85087 | 30S ribosomal protein S12 |
| XAC_RS02680 | XAC0514 | -1.44726 | CDP-alcohol phosphatidyltransferase family protein |
| XAC_RS07570 | XAC1480 | -1.15501 | LysR family transcriptional regulator |
| XAC_RS07890 | XAC1550 | 1.50911 | FKBP-type peptidyl-prolyl cis-trans isomerase |
| XAC_RS14385 | XAC2833 | -1.81198 | serine protease |
| XAC_RS11090 | - | -1.03369 | hypothetical protein |
| XAC_RS14305 | - | -1.76753 | hypothetical protein |
| XAC_RS12735 | XAC2505 | -1.91388 | hypothetical protein |
| XAC_RS03230 | XAC0621 | -1.35788 | DNA-binding response regulator |
| XAC_RS23385 | XAC1745 | -1.98052 | hypothetical protein |
| XAC_RS01130 | XAC0216 | -1.32601 | uroporphyrinogen III methyltransferase |
| XAC_RS00165 | XAC0031 | 1.00311 | NAD(P)-dependent alcohol dehydrogenase |
| pdxH | XAC3009 | -1.04201 | pyridoxine/pyridoxamine 5'-phosphate oxidase |
| XAC_RS18160 | XAC3592 | -1.52577 | hypothetical protein |
| XAC_RS03370 | XAC0649 | -1.63279 | quinoprotein dehydrogenase-associated putative ABC transporter substrate-binding protein |
| XAC_RS03930 | XAC0760 | -1.23438 | DNA-binding response regulator |
| XAC_RS03410 | XAC0657 | -1.59776 | rod shape-determining protein MreC |
| XAC_RS16100 | XAC3176 | -1.32557 | TonB-dependent siderophore receptor |
| XAC_RS05305 | XAC1037 | -1.55114 | phosphatase PAP2 family protein |
| XAC_RS10970 | XAC2159 | -1.33876 | siroheme synthase |
| XAC_RS22380 | XACb0032 | -1.67295 | type II toxin-antitoxin system PemK/MazF family toxin |
| XAC_RS13980 | XAC2754 | 1.99054 | peptidylprolyl isomerase |
| ribF | XAC1253 | -1.07161 | bifunctional riboflavin kinase/FAD synthetase |
| XAC_RS23515 | - | -2.12021 | hypothetical protein |
| XAC_RS18725 | XAC3701 | -1.51147 | Na+/H+ antiporter |
| XAC_RS18890 | XAC3739 | -1.38847 | hypothetical protein |
| XAC_RS02265 | XAC0433 | -1.7012 | NAD(P)-dependent oxidoreductase |
| XAC_RS22750 | XAC0092 | 1.15203 | hypothetical protein |
| XAC_RS19365 | XAC3836 | -1.49105 | hypothetical protein |
| XAC_RS01220 | XAC0234 | -1.1787 | Fic family protein |
| XAC_RS15205 | XAC2996 | -1.78709 | cupin-like domain-containing protein |
| typA | XAC1004 | 1.79958 | translational GTPase TypA |
| XAC_RS09015 | XAC1769 | -1.74826 | TonB-dependent receptor |
| XAC_RS16860 | XAC3326 | -1.32928 | AcrB/AcrD/AcrF family protein |
| XAC_RS22835 | - | -1.53921 | hypothetical protein |
| XAC_RS17550 | XAC3467 | -1.15002 | glycosyl transferase |
| XAC_RS03925 | XAC0759 | -1.46005 | sensor histidine kinase KdpD |
| XAC_RS18395 | XAC3638 | -1.83024 | efflux RND transporter periplasmic adaptor subunit |
| XAC_RS18030 | XAC3566 | 1.21711 | DUF4124 domain-containing protein |
| XAC_RS11635 | - | -1.59231 | hypothetical protein |
| XAC_RS02290 | XAC0437 | -1.51286 | TetR/AcrR family transcriptional regulator |
| XAC_RS10340 | XAC2033 | -1.48975 | molybdenum cofactor guanylyltransferase |
| XAC_RS24875 | XACb0073 | -1.51626 | hypothetical protein |
| XAC_RS06215 | XAC1218 | -1.41479 | phosphoethanolamine transferase |
| XAC_RS03770 | XAC0729 | -1.80352 | two-component sensor histidine kinase |
| XAC_RS07280 | - | 2.09103 | hypothetical protein |
| XAC_RS21190 | XAC4202 | -1.22469 | hypothetical protein |
| hrpB | XAC0293 | -1.55513 | ATP-dependent helicase HrpB |
| XAC_RS11525 | XAC2268 | -2.12352 | hypothetical protein |
| XAC_RS20985 | XAC4166 | -1.89615 | alkaline phosphatase |
| XAC_RS03520 | XAC0678 | 1.55016 | glycine zipper 2TM domain-containing protein |
| XAC_RS20880 | XAC4146 | -1.94904 | type VI secretion system contractile sheath large subunit |
| XAC_RS23595 | XAC2184 | -1.72954 | hypothetical protein |
| fliP | XAC1944 | -1.51094 | flagellar biosynthetic protein FliP |
| XAC_RS03945 | XAC0762 | -1.61238 | FAD-binding protein |
| XAC_RS03425 | XAC0660 | -1.57382 | rod shape-determining protein RodA |
| XAC_RS11605 | XAC2280 | -1.85116 | hypothetical protein |
| XAC_RS01605 | XAC0307 | -1.49656 | nucleoside hydrolase |
| XAC_RS04720 | XAC0920 | -1.08986 | DUF3106 domain-containing protein |
| XAC_RS11620 | XAC2283 | -1.71946 | TIGR03756 family integrating conjugative element protein |
| XAC_RS03575 | XAC0689 | -1.07424 | VanZ family protein |
| rnpB | - | 3.15475 | - |
| XAC_RS01705 | XAC0326 | -1.62695 | sensor histidine kinase efflux regulator BaeS |
| ptsP | XAC2979 | -1.09797 | phosphoenolpyruvate--protein phosphotransferase |
| XAC_RS06895 | XAC1349 | -1.20803 | autotransporter domain-containing protein |
| XAC_RS05185 | XAC1014 | -1.34424 | rRNA pseudouridine synthase |
| XAC_RS18320 | XAC3622 | -1.62054 | DNA polymerase IV |
| cas4 | - | -1.85866 | CRISPR-associated protein Cas4 |
| XAC_RS14020 | XAC2761 | 1.77186 | exodeoxyribonuclease 7 small subunit |
| XAC_RS18800 | XAC3718 | -1.57849 | hypothetical protein |
| XAC_RS03485 | XAC0671 | -1.16075 | LysR family transcriptional regulator |
| XAC_RS17440 | XAC3445 | -1.31856 | AraC family transcriptional regulator |
| XAC_RS08755 | XAC1718 | -3.82978 | hypothetical protein |
| XAC_RS03125 | XAC0600 | -1.13016 | D-serine/D-alanine/glycine transporter |
| pcaF | XAC0366 | -1.61107 | 3-oxoadipyl-CoA thiolase |
| XAC_RS14340 | XAC2824 | -1.23195 | alkaline phosphatase family protein |
| XAC_RS04165 | XAC0808 | -1.65399 | DUF4105 domain-containing protein |
| XAC_RS09525 | XAC1873 | -1.44692 | hypothetical protein |
| XAC_RS00055 | XAC0010 | 3.20228 | biopolymer transporter ExbD |
| XAC_RS21975 | XAC4358 | -1.2402 | sensor domain-containing diguanylate cyclase |
| XAC_RS18990 | XAC3758 | -1.63619 | DUF3526 domain-containing protein |
| XAC_RS20305 | XAC4031 | -1.20803 | ATP-dependent DNA helicase DinG |
| XAC_RS23775 | XAC2626 | -1.94258 | prepilin-type N-terminal cleavage/methylation domain-containing protein |
| XAC_RS09865 | XAC1938 | -1.31879 | bifunctional diguanylate cyclase/phosphodiesterase |
| XAC_RS18745 | XAC3706 | -1.51111 | dioxygenase |
| XAC_RS08605 | XAC1688 | -1.402 | sugar O-acyltransferase |
| XAC_RS00010 | XAC0001 | -1.35517 | chromosomal replication initiator protein DnaA |
| XAC_RS14440 | XAC2844 | -1.86833 | MexE family multidrug efflux RND transporter periplasmic adaptor subunit |
| XAC_RS05650 | XAC1104 | -1.89621 | plasmid mobilization protein |
| XAC_RS01930 | XAC0365 | -1.59407 | CoA-transferase subunit beta |
| ahpC | XAC0907 | 1.97511 | peroxiredoxin |
| XAC_RS14105 | XAC2778 | -1.67582 | nicotinate-nucleotide adenylyltransferase |
| XAC_RS00195 | - | -1.80845 | hypothetical protein |
| kdgT | XAC0337 | -1.62358 | 2-keto-3-deoxygluconate permease |
| ligD | XAC2414 | -1.33706 | DNA ligase D |
| XAC_RS05920 | XAC1160 | -1.50662 | NAD(P)-dependent oxidoreductase |
| XAC_RS05455 | - | -3.52675 | hypothetical protein |
| XAC_RS00065 | XAC0012 | -1.12926 | pyridoxine 5'-phosphate synthase |
| XAC_RS18260 | XAC3610 | -1.26261 | RNA helicase |
| XAC_RS09010 | XAC1768 | -1.45057 | TonB-dependent receptor |
| XAC_RS24315 | XAC3720 | -1.6035 | histidine kinase |
| XAC_RS16145 | XAC3185 | -1.10755 | fructose 2,6-bisphosphatase |
| XAC_RS04415 | XAC0860 | -1.67545 | ABC transporter ATP-binding protein |
| XAC_RS02055 | XAC0390 | -1.46044 | 4-hydroxybenzoate octaprenyltransferase |
| XAC_RS12525 | XAC2463 | -1.42375 | hypothetical protein |
| XAC_RS21810 | XAC4324 | -1.99849 | DUF1348 domain-containing protein |
| XAC_RS20960 | XAC4161 | -1.55575 | HlyD family secretion protein |
| XAC_RS11135 | XAC2190 | -1.88536 | hypothetical protein |
| XAC_RS02205 | XAC0421 | -1.67491 | phosphoglycerol transferase I |
| XAC_RS18130 | XAC3586 | 1.42717 | electron transfer flavoprotein subunit beta/FixA family protein |
| XAC_RS06240 | XAC1223 | -1.02855 | hypothetical protein |
| XAC_RS14980 | XAC2951 | 4.42015 | DNA transport competence protein |
| petA | XAC2457 | 1.51998 | ubiquinol-cytochrome c reductase iron-sulfur subunit |
| XAC_RS16880 | XAC3330 | -1.61313 | assimilatory sulfite reductase (NADPH) flavoprotein subunit |
| pstC | XAC1576 | -1.45387 | phosphate ABC transporter permease subunit PstC |
| XAC_RS01180 | XAC0226 | -1.23896 | sigma-54-dependent Fis family transcriptional regulator |
| XAC_RS11995 | XAC2356 | -1.46319 | MFS transporter |
| rimI | XAC3563 | -1.52618 | ribosomal-protein-alanine N-acetyltransferase |
| XAC_RS01315 | XAC0252 | -1.49685 | glyoxalase/bleomycin resistance/extradiol dioxygenase family protein |
| XAC_RS10875 | XAC2140 | -1.73822 | D-Ala-D-Ala carboxypeptidase |
| XAC_RS07820 | XAC1536 | -1.35303 | N-acetyltransferase |
| XAC_RS02050 | XAC0389 | -1.59923 | amidophosphoribosyltransferase |
| XAC_RS15885 | XAC3134 | -1.75562 | LysR family transcriptional regulator ArgP |
| XAC_RS02855 | XAC0548 | -1.70008 | SMP-30/gluconolactonase/LRE family protein |
| XAC_RS10670 | XAC2099 | -1.10393 | IS3 family transposase |
| XAC_RS09200 | XAC1808 | 1.33719 | aldehyde dehydrogenase family protein |
| hppD | XAC0452 | 1.96296 | 4-hydroxyphenylpyruvate dioxygenase |
| XAC_RS09160 | XAC1800 | -1.67005 | phosphatase PAP2 family protein |
| mgtE | XAC4078 | -1.0648 | magnesium transporter |
| XAC_RS14465 | XAC2849 | -1.59452 | nitrilase |
| XAC_RS08705 | XAC1708 | -1.26094 | exod protein |
| XAC_RS15610 | XAC3076 | -1.06213 | glycoside hydrolase family 3 |
| XAC_RS12910 | XAC2535 | -1.70663 | TonB-dependent receptor |
| XAC_RS05445 | XAC1063 | -2.01163 | lysozyme |
| XAC_RS09650 | XAC1896 | 2.16072 | methyl-accepting chemotaxis protein |
| XAC_RS07955 | XAC1564 | -1.22867 | acetylhydrolase |
| XAC_RS22535 | XACb0065 | -1.90554 | avirulence protein |
| XAC_RS18305 | XAC3619 | -1.78908 | alpha/beta hydrolase |
| XAC_RS11870 | XAC2331 | -1.54126 | tetratricopeptide repeat protein |
| XAC_RS15470 | XAC3047 | -1.00893 | monofunctional biosynthetic peptidoglycan transglycosylase |
| XAC_RS20795 | XAC4127 | -1.62577 | serine/threonine protein kinase |
| XAC_RS19935 | - | -1.23479 | competence protein |
| XAC_RS01300 | XAC0250 | -1.39973 | hydrolase |
| XAC_RS20280 | XAC4025 | -1.20409 | tRNA dihydrouridine(20/20a) synthase DusA |
| XAC_RS24330 | - | -1.97379 | hypothetical protein |
| XAC_RS11160 | XAC2195 | -1.3521 | DUF2628 domain-containing protein |
| XAC_RS02105 | XAC0401 | -2.23579 | EscS/YscS/HrcS family type III secretion system export apparatus protein |
| XAC_RS11285 | XAC2221 | -1.9936 | DUF3085 domain-containing protein |
| XAC_RS21140 | XAC4192 | -1.93068 | membrane protein |
| XAC_RS15860 | XAC3129 | -1.14137 | RNA-binding protein S4 |
| XAC_RS01585 | XAC0303 | -1.40384 | MFS transporter |
| XAC_RS08445 | XAC1658 | -1.93655 | hypothetical protein |
| XAC_RS02270 | XAC0434 | -1.84323 | FAD-binding protein |
| XAC_RS18570 | XAC3671 | 1.30667 | YajQ family cyclic di-GMP-binding protein |
| XAC_RS21320 | XAC4226 | -1.38175 | LacI family DNA-binding transcriptional regulator |
| XAC_RS12380 | XAC2435 | 1.79769e+308 | hypothetical protein |
| XAC_RS05780 | XAC1131 | -1.05561 | endolytic transglycosylase MltG |
| XAC_RS16290 | XAC3213 | -1.39291 | methyl-accepting chemotaxis protein |
| XAC_RS00145 | XAC0028 | -1.53947 | cellulase |
| XAC_RS13230 | XAC2598 | -1.23781 | hypothetical protein |
| XAC_RS24760 | - | -2.02803 | transposase |
| XAC_RS04070 | XAC0789 | -1.35642 | 8-oxo-dGTP diphosphatase MutT |
| XAC_RS19995 | XAC3967 | -1.73424 | alpha/beta hydrolase |
| XAC_RS24805 | - | -1.75509 | YecA family protein |
| XAC_RS12265 | XAC2412 | -1.53209 | formylglycine-generating enzyme family protein |
| XAC_RS10925 | XAC2150 | -1.68093 | DNA-binding response regulator |
| XAC_RS16745 | XAC3306 | -2.0193 | hypothetical protein |
| XAC_RS04830 | XAC0943 | -1.43158 | hypothetical protein |
| XAC_RS15650 | XAC3084 | -1.27269 | beta-galactosidase |
| XAC_RS23150 | XAC1162 | -1.71821 | hypothetical protein |
| XAC_RS19275 | XAC3818 | -1.64442 | primosomal protein N' |
| XAC_RS13495 | XAC2655 | -1.52279 | baseplate assembly protein |
| XAC_RS05255 | XAC1028 | -1.02335 | histidine phosphatase family protein |
| XAC_RS16095 | XAC3175 | -1.0304 | 4-hydroxy-2-oxovalerate aldolase |
| XAC_RS11735 | XAC2304 | -1.04665 | N-acetyltransferase |
| XAC_RS19280 | XAC3819 | -1.57619 | glutathione S-transferase |
| XAC_RS17195 | XAC3394 | 2.77922 | DNA-directed RNA polymerase subunit omega |
| XAC_RS04365 | XAC0849 | -1.71268 | aliphatic sulfonate ABC transporter substrate-binding protein |
| XAC_RS16300 | XAC3215 | -1.08512 | DUF4166 domain-containing protein |
| flhB | XAC1937 | -1.65373 | flagellar biosynthesis protein FlhB |
| XAC_RS04600 | - | -1.79769e+308 | DUF2007 domain-containing protein |
| XAC_RS21480 | XAC4257 | -1.41615 | MFS transporter |
| XAC_RS09930 | XAC1951 | -1.27966 | FliI/YscN family ATPase |
| XAC_RS11960 | XAC2349 | -1.45587 | acetylornithine deacetylase |
| XAC_RS22525 | XACb0061 | -1.49563 | recombinase family protein |
| XAC_RS03600 | XAC0694 | -1.56847 | PDZ domain-containing protein |
| XAC_RS06550 | XAC1284 | 2.61343 | response regulator |
| XAC_RS05705 | XAC1115 | -1.34202 | DUF58 domain-containing protein |
| XAC_RS10205 | XAC2007 | -1.13568 | DUF3857 domain-containing protein |
| XAC_RS13730 | XAC2706 | 2.29523 | preprotein translocase subunit SecG |
| XAC_RS21300 | XAC4222 | -1.35962 | M48 family peptidase |
| XAC_RS17100 | XAC3374 | -1.48087 | membrane protein |
| XAC_RS04380 | XAC0852 | -1.93235 | TonB-dependent receptor |
| XAC_RS08275 | XAC1625 | -1.89759 | hypothetical protein |
| XAC_RS11110 | XAC2185 | -1.81652 | TonB-dependent siderophore receptor |
| XAC_RS21155 | XAC4194 | -1.89524 | hypothetical protein |
| XAC_RS14950 | XAC2945 | -1.43114 | DUF2271 domain-containing protein |
| XAC_RS22895 | - | -1.53063 | DUF3426 domain-containing protein |
| XAC_RS11400 | XAC2243 | -1.90495 | hypothetical protein |
| XAC_RS08225 | XAC1616 | -1.97499 | hypothetical protein |
| XAC_RS14150 | XAC2786 | -1.75502 | hypothetical protein |
| XAC_RS17355 | XAC3428 | -1.12349 | hydrolase |
| XAC_RS21950 | XAC4352 | -1.29635 | glutathione S-transferase |
| XAC_RS01020 | XAC0195 | -1.40579 | cardiolipin synthase |
| XAC_RS07485 | XAC1464 | -1.336 | DUF456 domain-containing protein |
| XAC_RS08175 | XAC1607 | -1.68861 | DUF2589 domain-containing protein |
| XAC_RS18850 | XAC3730 | -1.21197 | methyltransferase |
| XAC_RS00720 | XAC0138 | -1.53541 | hypothetical protein |
| XAC_RS11615 | XAC2282 | -1.15108 | TIGR03757 family integrating conjugative element protein |
| XAC_RS09615 | XAC1890 | 2.24594 | chemotaxis protein CheR |
| recJ | XAC1865 | -1.35095 | single-stranded-DNA-specific exonuclease RecJ |
| XAC_RS19035 | XAC3767 | -1.83977 | hypothetical protein |
| XAC_RS18145 | XAC3589 | -1.16668 | membrane protein |
| XAC_RS17320 | XAC3421 | -1.55065 | LuxR family transcriptional regulator |
| XAC_RS22905 | - | -1.52284 | hypothetical protein |
| XAC_RS11260 | XAC2216 | -1.49413 | hypothetical protein |
| XAC_RS00590 | XAC0114 | -1.57118 | carboxypeptidase regulatory-like domain-containing protein |
| XAC_RS10225 | XAC2011 | -1.76526 | camphor resistance protein CrcB |
| XAC_RS17365 | XAC3430 | -1.41741 | ion transporter |
| XAC_RS09245 | XAC1818 | -1.27147 | S-layer family protein |
| XAC_RS06320 | XAC1238 | -1.13063 | endonuclease/exonuclease/phosphatase family protein |
| XAC_RS02805 | XAC0538 | -1.55114 | hypothetical protein |
| XAC_RS18585 | XAC3674 | -1.45358 | hypothetical protein |
| XAC_RS19565 | XAC3877 | -1.36877 | bile acid:sodium symporter |
| ribD | XAC0746 | -1.46118 | bifunctional diaminohydroxyphosphoribosylaminopyrimidine deaminase/5-amino-6-(5-phosphoribosylamino)uracil reductase RibD |
| XAC_RS00545 | XAC0107 | -1.67532 | Zn-dependent protease with chaperone function |
| XAC_RS00320 | XAC0062 | -1.39492 | hypothetical protein |
| XAC_RS05060 | XAC0989 | 2.60402 | 30S ribosomal protein S5 |
| XAC_RS16185 | XAC3193 | -1.28283 | hypothetical protein |
| XAC_RS05765 | XAC1128 | 3.66436 | acyl carrier protein |
| XAC_RS07845 | XAC1541 | -1.53858 | HlyC/CorC family transporter |
| cydC | XAC2334 | -1.38311 | thiol reductant ABC exporter subunit CydC |
| XAC_RS16925 | XAC3339 | -1.03608 | LysR family transcriptional regulator |
| XAC_RS06175 | - | -1.5882 | hypothetical protein |
| XAC_RS16305 | XAC3216 | -1.48413 | peptidoglycan editing factor PgeF |
| XAC_RS24850 | - | -1.57037 | hypothetical protein |
| XAC_RS01670 | XAC0319 | -1.65338 | alpha/beta hydrolase |
| XAC_RS15175 | XAC2989 | -1.3667 | amino acid permease |
| XAC_RS13180 | XAC2588 | 1.19526 | integration host factor subunit alpha |
| XAC_RS21250 | XAC4213 | -1.86777 | type III secretion system effector XopAD |
| XAC_RS14415 | XAC2839 | -1.20044 | AraC family transcriptional regulator |
| XAC_RS13570 | XAC2674 | -1.07594 | membrane protein |
| XAC_RS01750 | XAC0335 | -1.21366 | DUF1852 domain-containing protein |
| cyoD | XAC1261 | 2.64887 | cytochrome o ubiquinol oxidase subunit IV |
| XAC_RS16420 | XAC3238 | -1.04628 | sigma-54-dependent Fis family transcriptional regulator |
| XAC_RS02885 | XAC0553 | -1.28618 | nucleoside-diphosphate sugar epimerase |
| XAC_RS11430 | - | -1.83872 | hypothetical protein |
| XAC_RS22190 | - | -2.57055 | hypothetical protein |
| XAC_RS08220 | XAC1615 | -1.16527 | FMN-binding negative transcriptional regulator |
| XAC_RS20995 | - | -1.71316 | 4-phosphopantetheinyl transferase |
| XAC_RS02170 | XAC0414 | -1.64007 | EscT/YscT/HrcT family type III secretion system export apparatus protein |
| XAC_RS00900 | XAC0173 | -1.23032 | DUF4375 domain-containing protein |
| psd | XAC2728 | -1.1745 | phosphatidylserine decarboxylase proenzyme |
| XAC_RS06170 | XAC1208 | -1.79422 | hypothetical protein |
| XAC_RS05515 | XAC1079 | 1.53653 | ATP-dependent Clp protease ATP-binding subunit ClpX |
| XAC_RS12140 | XAC2387 | -1.63735 | ribonuclease |
| XAC_RS04490 | XAC0876 | -1.87815 | hypothetical protein |
| XAC_RS00715 | - | -1.23353 | hypothetical protein |
| XAC_RS17935 | XAC3548 | -1.59656 | adhesin |
| XAC_RS02910 | XAC0558 | -1.53729 | siderophore-interacting protein |
| XAC_RS22980 | - | -2.63163 | hypothetical protein |
| XAC_RS20895 | XAC4149 | -1.61319 | universal stress protein |
| XAC_RS11485 | XAC2260 | -1.78774 | TIGR03747 family integrating conjugative element membrane protein |
| pobA | XAC0356 | -1.7511 | 4-hydroxybenzoate 3-monooxygenase |
| cyoA | - | 2.30587 | ubiquinol oxidase subunit II |
| XAC_RS00395 | XAC0076 | -1.77141 | avirulence protein |
| XAC_RS12470 | XAC2452 | -2.02325 | hypothetical protein |
| XAC_RS10170 | XAC2000 | 1.38563 | ATP-dependent Clp protease adaptor ClpS |
| XAC_RS08565 | XAC1681 | -1.3996 | hypothetical protein |
| XAC_RS21205 | - | 1.79769e+308 | hypothetical protein |
| XAC_RS11150 | XAC2193 | -1.71888 | TonB-dependent receptor |
| XAC_RS19470 | XAC3857 | -1.29682 | hypothetical protein |
| XAC_RS22955 | XAC0624 | -1.86309 | hypothetical protein |
| XAC_RS18965 | - | -1.62151 | chemotaxis protein |
| XAC_RS20740 | XAC4116 | -1.90674 | protein kinase |
| XAC_RS20070 | XAC3983 | 2.54265 | hypothetical protein |
| XAC_RS22370 | XACb0030 | -1.71829 | TrwB protein |
| XAC_RS19775 | XAC3920 | -1.4968 | acyltransferase |
| XAC_RS01030 | XAC0197 | -1.19472 | 1-acyl-sn-glycerol-3-phosphate acyltransferase |
| eat | XAC2364 | -1.64868 | ethanolamine permease |
| XAC_RS18815 | XAC3721 | -1.34763 | FAD-binding oxidoreductase |
| rnt | XAC1571 | -1.01462 | ribonuclease T |
| XAC_RS06485 | XAC1271 | -1.00975 | anti-sigma regulatory factor |
| XAC_RS02305 | XAC0440 | -1.55156 | oxidoreductase |
| XAC_RS11215 | XAC2207 | -1.87822 | DUF2857 domain-containing protein |
| uraH | XAC0295 | -1.66855 | hydroxyisourate hydrolase |
| dprA | XAC3803 | -1.56847 | DNA-protecting protein DprA |
| XAC_RS24790 | - | -1.45936 | hypothetical protein |
| XAC_RS09280 | XAC1825 | -1.15332 | Crp/Fnr family transcriptional regulator |
| XAC_RS13485 | XAC2652 | -1.89869 | tail protein |
| XAC_RS08215 | XAC1614 | -1.83361 | hypothetical protein |
| XAC_RS03030 | XAC0583 | -1.47258 | oxidoreductase |
| cobO | XAC3191 | -1.68402 | cob(I)yrinic acid a,c-diamide adenosyltransferase |
| XAC_RS09995 | XAC1965 | 1.63534 | acyl carrier protein |
| XAC_RS09055 | XAC1777 | -1.04614 | MFS transporter |
| XAC_RS00640 | XAC0124 | 1.07656 | fructose 1,6-bisphosphatase |
| XAC_RS03920 | XAC0758 | -1.49997 | potassium-transporting ATPase subunit KdpC |
| rplU | XAC1248 | 2.50168 | 50S ribosomal protein L21 |
| XAC_RS08895 | XAC1747 | -1.74584 | pirin family protein |
| XAC_RS16045 | XAC3166 | -1.16873 | TonB-dependent receptor |
| XAC_RS04520 | XAC0882 | -1.60179 | aldehyde dehydrogenase |
| XAC_RS06510 | XAC1276 | -1.57094 | TonB-dependent receptor |
| XAC_RS01915 | XAC0362 | -1.72521 | oxidoreductase |
| XAC_RS20780 | XAC4124 | -1.65633 | type VI secretion system tip protein VgrG |
| XAC_RS19075 | - | -2.75235 | DUF1653 domain-containing protein |
| XAC_RS22425 | XACb0041 | -1.12613 | type VI secretion protein |
| XAC_RS17510 | XAC3459 | -1.56123 | LysR family transcriptional regulator |
| XAC_RS17575 | XAC3472 | 3.26022 | porin |
| XAC_RS08905 | XAC1748 | -1.30428 | LysR family transcriptional regulator |
| XAC_RS05925 | XAC1161 | -1.21602 | TetR/AcrR family transcriptional regulator |
| XAC_RS02900 | - | -1.41913 | hypothetical protein |
| ssuD | XAC0850 | -1.72892 | alkanesulfonate monooxygenase |
| XAC_RS05815 | XAC1138 | 1.55195 | 2-methylcitrate synthase |
| XAC_RS01335 | XAC0256 | -1.37391 | malate synthase A |
| XAC_RS01465 | XAC0281 | -1.29047 | MarR family transcriptional regulator |
| XAC_RS11225 | - | -1.91374 | integrating conjugative element protein |
| XAC_RS20670 | XAC4102 | -1.1965 | NAD(P)/FAD-dependent oxidoreductase |
| XAC_RS13455 | XAC2646 | -1.72729 | phage capsid completion protein |
| XAC_RS08890 | XAC1746 | 1.17434 | PAS domain S-box protein |
| XAC_RS03935 | - | -1.79769e+308 | hypothetical protein |
| XAC_RS20215 | XAC4013 | 1.5909 | GTPase |
| XAC_RS05700 | XAC1114 | -1.57113 | DUF3488 domain-containing protein |
| XAC_RS00220 | XAC0043 | -1.48052 | undecaprenyl-phosphate glucose phosphotransferase |
| XAC_RS05450 | XAC1064 | -1.72623 | phage-related DNA maturase |
| XAC_RS10805 | XAC2128 | -1.31422 | GMC family oxidoreductase |
| XAC_RS23360 | - | -1.79769e+308 | AlpA family phage regulatory protein |
| XAC_RS04155 | XAC0806 | -1.37443 | phosphoenolpyruvate carboxylase |
| XAC_RS22720 | XAC0071 | -1.78293 | hypothetical protein |
| XAC_RS21465 | XAC4254 | -1.40364 | beta-1,4-xylanase |
| fucP | XAC4190 | -1.09749 | L-fucose:H+ symporter permease |
| XAC_RS07745 | XAC1522 | 2.52976 | molecular chaperone DnaK |
| XAC_RS05050 | XAC0987 | 1.82214 | 50S ribosomal protein L6 |
| XAC_RS05460 | XAC1066 | -3.34843 | hypothetical protein |
| XAC_RS13625 | XAC2685 | -1.34911 | tRNA pseudouridine(55) synthase TruB |
| XAC_RS02620 | XAC0504 | -1.43545 | membrane protein |
| XAC_RS23650 | - | -1.80107 | hypothetical protein |
| XAC_RS00785 | XAC0149 | -1.58294 | hypothetical protein |
| XAC_RS00730 | XAC0140 | -1.52008 | transcriptional regulator |
| XAC_RS21595 | XAC4283 | -1.40085 | sensor histidine kinase |
| XAC_RS17815 | XAC3523 | -1.16944 | hypothetical protein |
| XAC_RS13100 | XAC2572 | -1.44825 | TraB/GumN family protein |
| XAC_RS09580 | XAC1883 | 1.98622 | AbrB/MazE/SpoVT family DNA-binding domain-containing protein |
| XAC_RS04180 | XAC0811 | -1.81324 | TonB-dependent receptor |
| XAC_RS09410 | XAC1851 | -1.27747 | hydroxymethylglutaryl-CoA lyase |
| XAC_RS11275 | XAC2219 | -1.28484 | hypothetical protein |
| XAC_RS08925 | XAC1752 | -1.16158 | hypothetical protein |
| XAC_RS00860 | XAC0164 | -1.45651 | TRAP transporter large permease |
| XAC_RS13995 | XAC2756 | -1.10421 | acyl-CoA thioesterase |
| XAC_RS18555 | XAC3668 | -1.54223 | ABC transporter permease |
| XAC_RS23605 | - | -1.74114 | Ig family protein |
| proB | XAC2343 | -1.34655 | glutamate 5-kinase |
| XAC_RS23675 | - | -2.65598 | KfrA protein |
| XAC_RS14185 | XAC2794 | -1.08895 | DUF4349 domain-containing protein |
| XAC_RS09230 | XAC1814 | -1.47056 | ShlB/FhaC/HecB family hemolysin secretion/activation protein |
| XAC_RS13390 | XAC2631 | -3.02813 | hypothetical protein |
| XAC_RS20610 | XAC4090 | -1.1502 | 3-oxoacyl-ACP reductase FabG |
| XAC_RS01720 | XAC0329 | -1.7921 | hypothetical protein |
| XAC_RS14665 | XAC2888 | -1.55478 | nucleotidyltransferase family protein |
| XAC_RS02800 | XAC0537 | -1.58789 | hypothetical protein |
| XAC_RS21135 | XAC4191 | -1.44291 | IclR family transcriptional regulator |
| XAC_RS06805 | XAC1332 | -1.70537 | FAD-binding protein |
| XAC_RS07195 | XAC1407 | -1.1229 | ribonuclease HII |
| XAC_RS02200 | XAC0420 | -1.37815 | DUF3772 domain-containing protein |
| XAC_RS13945 | XAC2746 | -1.13969 | M13 family peptidase |
| XAC_RS01615 | XAC0309 | -1.47729 | NCS2 family permease |
| XAC_RS05135 | XAC1003 | -1.32921 | DUF2127 domain-containing protein |
| XAC_RS19000 | XAC3760 | -1.75109 | hypothetical protein |
| XAC_RS24600 | XAC4263 | -1.8223 | hypothetical protein |
| XAC_RS06575 | XAC1288 | -1.56967 | membrane protein |
| XAC_RS14335 | XAC2823 | -1.28582 | methylated-DNA--protein-cysteine methyltransferase |
| XAC_RS01950 | XAC0369 | -1.50635 | 3-carboxy-cis,cis-muconate cycloisomerase |
| XAC_RS07715 | XAC1515 | -2.1776 | RnfH family protein |
| XAC_RS21415 | XAC4246 | -1.68898 | DUF2147 domain-containing protein |
| XAC_RS23330 | - | -1.64528 | oxidoreductase |
| pncB | XAC3521 | -1.17603 | nicotinate phosphoribosyltransferase |
| XAC_RS03360 | XAC0647 | -1.64608 | HAD family hydrolase |
| XAC_RS23570 | XAC2146 | -1.76804 | hypothetical protein |
| XAC_RS15510 | XAC3055 | -2.05965 | DUF1304 domain-containing protein |
| XAC_RS11345 | XAC2232 | -1.34757 | NAD(P)H:quinone oxidoreductase |
| XAC_RS12970 | XAC2547 | 2.70463 | dihydrodipicolinate synthase family protein |
| ppk1 | XAC1040 | -1.04864 | polyphosphate kinase 1 |
| XAC_RS19165 | XAC3796 | -1.38713 | glycosyltransferase family 2 protein |
| XAC_RS02195 | XAC0419 | 2.37991 | hypothetical protein |
| XAC_RS03910 | XAC0756 | -1.65766 | potassium-transporting ATPase subunit KdpA |
| XAC_RS04290 | XAC0834 | 5.00464 | DNA-binding response regulator |
| XAC_RS09395 | XAC1848 | -1.29187 | hypothetical protein |
| XAC_RS17825 | XAC3525 | -1.12635 | hypothetical protein |
| XAC_RS23985 | XAC3111 | -2.16109 | hypothetical protein |
| XAC_RS10660 | XAC2097 | -1.80955 | non-ribosomal peptide synthase |
| XAC_RS07595 | XAC1484 | -1.29281 | NAD(P)-dependent oxidoreductase |
| XAC_RS01965 | XAC0372 | -1.28674 | alpha/beta hydrolase |
| XAC_RS24655 | - | -3.24342 | hypothetical protein |
| XAC_RS04910 | XAC0959 | -1.77376 | preprotein translocase subunit SecE |
| XAC_RS20910 | XAC4152 | -1.33749 | pyridine nucleotide-disulfide oxidoreductase |
| XAC_RS00200 | XAC0038 | -1.68507 | NmrA family transcriptional regulator |
| XAC_RS12120 | XAC2383 | -1.30282 | phosphate-binding protein |
| XAC_RS07250 | XAC1418 | 1.95999 | ribosome-recycling factor |
| XAC_RS02615 | XAC0503 | -1.38585 | NAD(+) diphosphatase |
| XAC_RS04265 | XAC0829 | -1.68962 | ABC transporter substrate-binding protein |
| XAC_RS15155 | XAC2986 | -1.71018 | pectate lyase |
| XAC_RS19125 | XAC3789 | -1.54562 | D-aminoacyl-tRNA deacylase |
| XAC_RS03115 | XAC0598 | -1.21367 | cyclohexadienyl dehydratase |
| XAC_RS24570 | - | -2.0222 | hypothetical protein |
| XAC_RS11685 | XAC2294 | -1.39256 | LPS biosynthesis protein |
| XAC_RS21840 | XAC4330 | 3.75701 | hypothetical protein |
| XAC_RS09795 | XAC1924 | -1.80838 | transposase |
| XAC_RS24120 | - | -1.58252 | IS3 family transposase |
| XAC_RS18835 | XAC3727 | -1.85816 | hypothetical protein |
| XAC_RS00285 | XAC0056 | -1.62073 | sugar transporter |
| XAC_RS00110 | XAC0021 | 1.29664 | DUF1820 domain-containing protein |
| XAC_RS07080 | - | -1.49768 | ferredoxin |
| XAC_RS15775 | XAC3110 | -1.48192 | glycosyl transferase |
| XAC_RS16115 | XAC3179 | -1.49549 | MFS transporter |
| XAC_RS17535 | XAC3464 | -1.27972 | 3-deoxy-D-manno-octulosonic acid transferase |
| XAC_RS21850 | XAC4333 | -1.90095 | type III secretion system effector protein |
| rplQ | XAC0997 | 2.14027 | 50S ribosomal protein L17 |
| XAC_RS16545 | XAC3266 | -1.1145 | hypothetical protein |
| XAC_RS20575 | XAC4084 | -1.38975 | ankyrin repeat protein |
| XAC_RS08140 | XAC1600 | -1.49149 | kynurenine 3-monooxygenase |
| XAC_RS07760 | XAC1525 | -1.12159 | prephenate dehydrogenase/arogenate dehydrogenase family protein |
| XAC_RS21765 | - | -1.46734 | enterochelin esterase |
| XAC_RS22090 | - | -1.41098 | PIN domain-containing protein |
| XAC_RS01205 | XAC0231 | -1.72568 | hypothetical protein |
| XAC_RS04545 | XAC0887 | -1.52277 | gluconolactonase |
| XAC_RS23805 | - | -1.80018 | hypothetical protein |
| XAC_RS03680 | XAC0711 | -1.13303 | GntR family transcriptional regulator |
| ccmB | XAC2324 | -1.83448 | heme exporter protein CcmB |
| XAC_RS17765 | XAC3514 | -1.53806 | serine protease |
| XAC_RS01135 | XAC0217 | -1.29245 | glycosyl transferase |
| XAC_RS16415 | XAC3237 | -1.64366 | sensor histidine kinase |
| XAC_RS20270 | XAC4023 | 1.17013 | DNA-binding response regulator |
| XAC_RS21120 | - | -2.85224 | hypothetical protein |
| XAC_RS02540 | XAC0488 | 2.99007 | 30S ribosomal protein S9 |
| XAC_RS07730 | XAC1519 | -1.02517 | DNA repair protein RecN |
| XAC_RS16440 | XAC3240 | 2.08125 | pilin |
| XAC_RS12605 | XAC2478 | -1.48847 | glutamine synthetase |
| XAC_RS23720 | - | 1.79769e+308 | hypothetical protein |
| XAC_RS12065 | XAC2370 | -1.26102 | hypothetical protein |
| XAC_RS23920 | - | -1.15375 | hypothetical protein |
| XAC_RS13475 | XAC2650 | -1.7515 | lysozyme |
| XAC_RS11705 | XAC2298 | 3.22127 | 30S ribosomal protein S1 |
| XAC_RS02895 | - | 2.13813 | polyisoprenoid-binding protein |
| XAC_RS20805 | XAC4129 | -1.55266 | RNA polymerase sigma factor |
| XAC_RS04235 | XAC0823 | -1.57681 | TonB-dependent hemoglobin/transferrin/lactoferrin family receptor |
| XAC_RS07410 | XAC1449 | -1.3672 | hypothetical protein |
| XAC_RS02695 | XAC0517 | -1.78851 | hypothetical protein |
| XAC_RS20990 | XAC4167 | -1.76204 | alkaline phosphatase |
| XAC_RS04160 | XAC0807 | -1.72241 | TetR/AcrR family transcriptional regulator |
| fliS | XAC1973 | 3.42951 | flagellar export chaperone FliS |
| XAC_RS00240 | XAC0047 | -1.53554 | glycosyltransferase family 1 protein |
| XAC_RS02080 | XAC0396 | -1.70449 | HpaB protein |
| XAC_RS10120 | XAC1990 | 1.51239 | flagella protein |
| XAC_RS18610 | XAC3678 | -1.08079 | rRNA pseudouridine synthase |
| XAC_RS02595 | XAC0499 | 1.97108 | iron-sulfur cluster insertion protein ErpA |
| XAC_RS04690 | XAC0914 | -1.38655 | DUF1631 domain-containing protein |
| XAC_RS15515 | XAC3056 | -1.12477 | MFS transporter |
| XAC_RS05805 | XAC1136 | -1.41014 | propionate catabolism operon regulatory protein PrpR |
| XAC_RS08365 | XAC1643 | 1.53968 | poly(hydroxyalkanoate) granule-associated domain protein |
| XAC_RS02005 | XAC0380 | -1.61707 | YdcF family protein |
| XAC_RS01165 | XAC0223 | 4.41128 | Ax21 family protein |
| XAC_RS04765 | - | -1.57672 | peptidase S8 |
| XAC_RS15145 | XAC2984 | -1.35178 | peptidase |
| XAC_RS12190 | XAC2397 | -1.19632 | NAD(P)/FAD-dependent oxidoreductase |
| sucB | XAC1534 | 1.91167 | dihydrolipoyllysine-residue succinyltransferase |
| XAC_RS18435 | XAC3645 | -2.01999 | GtrA family protein |
| XAC_RS10985 | XAC2161 | -1.53117 | MFS transporter |
| XAC_RS08155 | XAC1603 | -1.30825 | 3-hydroxyanthranilate 3,4-dioxygenase |
| XAC_RS00710 | XAC0136 | -1.65075 | DNA-binding response regulator |
| XAC_RS21310 | XAC4224 | -1.01732 | tRNA 2-thiocytidine(32) synthetase TtcA |
| XAC_RS10010 | XAC1968 | 1.94967 | DNA-binding response regulator |
| XAC_RS03165 | XAC0607 | -1.64746 | hypothetical protein |
| treY | XAC0429 | -1.43993 | malto-oligosyltrehalose synthase |
| XAC_RS07295 | XAC1426 | -1.69023 | molecular chaperone |
| XAC_RS19510 | XAC3865 | -1.39968 | hypothetical protein |
| XAC_RS17010 | XAC3357 | -1.1447 | MBL fold metallo-hydrolase |
| XAC_RS15625 | XAC3079 | -1.4623 | amino acid permease |
| XAC_RS15360 | XAC3025 | -2.05051 | hypothetical protein |
| XAC_RS15450 | XAC3043 | -1.79074 | hemolysin D |
| XAC_RS10355 | XAC2036 | -1.22494 | divalent metal cation transporter |
| XAC_RS19350 | XAC3833 | -1.5157 | biotin transporter BioY |
| XAC_RS16875 | XAC3329 | -1.69997 | sulfate adenylyltransferase subunit 2 |
| XAC_RS20675 | XAC4103 | -1.08903 | hypothetical protein |
| XAC_RS17110 | XAC3376 | -1.61208 | VWA domain-containing protein |
| XAC_RS17135 | XAC3382 | 1.7145 | fimbrial protein |
| XAC_RS08640 | XAC1695 | -1.85255 | hypothetical protein |
| XAC_RS16950 | XAC3344 | 2.04251 | fructose-bisphosphate aldolase class I |
| XAC_RS22175 | XACa0027 | -1.45267 | plasmid stable inheritance protein K |
| XAC_RS08450 | XAC1659 | -1.61763 | hypothetical protein |
| XAC_RS07600 | XAC1485 | -1.30443 | RND transporter |
| XAC_RS11915 | XAC2340 | -1.5119 | MFS transporter |
| XAC_RS07915 | XAC1555 | -1.30795 | LacI family DNA-binding transcriptional regulator |
| XAC_RS13540 | XAC2665 | 3.27179 | pilus assembly protein |
| XAC_RS08690 | XAC1705 | -1.50533 | MFS transporter |
| XAC_RS24755 | - | -1.47842 | XRE family transcriptional regulator |
| pstA | XAC1575 | -1.421 | phosphate ABC transporter, permease protein PstA |
| XAC_RS00225 | XAC0044 | -1.29757 | UDP-N-acetylglucosamine 2-epimerase (non-hydrolyzing) |
| XAC_RS11190 | XAC2202 | -1.67981 | type I secretion system permease/ATPase |
| XAC_RS12475 | XAC2453 | 1.18994 | ClpXP protease specificity-enhancing factor |
| XAC_RS06000 | XAC1176 | -1.29881 | glycosyl hydrolase |
| XAC_RS09260 | XAC1821 | -1.52717 | homoserine kinase |
| XAC_RS07010 | XAC1370 | -1.68993 | DUF998 domain-containing protein |
| dapE | XAC1432 | -1.04951 | succinyl-diaminopimelate desuccinylase |
| bfr | XAC1438 | 1.32089 | bacterioferritin |
| XAC_RS09295 | XAC1828 | -1.6383 | ATP phosphoribosyltransferase |
| XAC_RS10345 | XAC2034 | -1.04564 | hypothetical protein |
| XAC_RS14125 | XAC2782 | -1.25331 | DUF998 domain-containing protein |
| XAC_RS16010 | XAC3158 | -1.98777 | TonB-dependent receptor |
| XAC_RS14975 | XAC2950 | -1.14215 | hypothetical protein |
| XAC_RS03320 | XAC0640 | -1.17209 | GNAT family N-acetyltransferase |
| XAC_RS21660 | XAC4296 | -1.03656 | lytic murein transglycosylase |
| XAC_RS14575 | XAC2871 | -1.14041 | phospholipase D family protein |
| XAC_RS18310 | XAC3620 | -1.92865 | TonB-dependent siderophore receptor |
| XAC_RS18490 | XAC3657 | 4.65486 | hypothetical protein |
| XAC_RS01655 | XAC0316 | -1.5377 | LysR family transcriptional regulator |
| XAC_RS19005 | XAC3761 | -1.58023 | LysR family transcriptional regulator |
| XAC_RS13740 | XAC2708 | -1.54163 | KR domain-containing protein |
| XAC_RS03640 | XAC0702 | -1.79365 | type II secretion system protein K |
| XAC_RS02965 | XAC0569 | -1.67884 | haloacid dehalogenase |
| XAC_RS11045 | XAC2173 | -1.57362 | DUF2309 domain-containing protein |
| XAC_RS13975 | XAC2752 | -1.39306 | DUF418 domain-containing protein |
| XAC_RS12415 | XAC2441 | -1.77336 | replication protein |
| XAC_RS17795 | XAC3520 | -1.22298 | hypothetical protein |
| XAC_RS14670 | XAC2889 | -1.05431 | IS3 family transposase |
| XAC_RS07755 | XAC1524 | -1.04631 | pyridoxal kinase |
| XAC_RS20735 | XAC4115 | -1.64336 | hypothetical protein |
| XAC_RS17810 | XAC3522 | -1.36964 | hypothetical protein |
| XAC_RS16070 | XAC3170 | -1.55792 | cytochrome P450 |
| XAC_RS10155 | XAC1997 | -1.01651 | high frequency lysogenization protein HflD |
| XAC_RS21710 | XAC4305 | -1.64286 | efflux RND transporter periplasmic adaptor subunit |
| XAC_RS06110 | XAC1197 | -1.62107 | DNA lesion error-prone repair protein ImuA |
| XAC_RS08970 | XAC1761 | 1.17748 | hypothetical protein |
| cls | XAC4153 | -1.71878 | cardiolipin synthase |
| XAC_RS00265 | XAC0052 | -1.61179 | hypothetical protein |
| XAC_RS07970 | XAC1568 | -1.87251 | GFA family protein |
| XAC_RS01035 | XAC0198 | -1.68684 | alpha/beta hydrolase |
| XAC_RS21025 | XAC4173 | -1.17857 | hypothetical protein |
| XAC_RS20255 | XAC4020 | -1.53791 | DUF1501 domain-containing protein |
| XAC_RS05470 | XAC1069 | -2.13573 | hypothetical protein |
| XAC_RS13525 | - | -1.12222 | IS3 family transposase |
| tuf | XAC0970 | 3.44015 | elongation factor Tu |
| XAC_RS07990 | - | 1.17076 | integral membrane protein |
| XAC_RS21020 | XAC4172 | -1.68871 | ROK family transcriptional regulator |
| XAC_RS07740 | XAC1521 | 1.57985 | nucleotide exchange factor GrpE |
| XAC_RS06630 | XAC1298 | -1.17925 | YafY family transcriptional regulator |
| XAC_RS07190 | XAC1406 | -1.11662 | DNA polymerase III subunit alpha |
| XAC_RS00585 | XAC0113 | -1.27311 | DUF938 domain-containing protein |
| XAC_RS15555 | XAC3065 | -1.39517 | nucleoside triphosphate pyrophosphohydrolase |
| kynU | XAC1601 | -1.05102 | kynureninase |
| XAC_RS21895 | XAC4342 | 1.77302 | organic solvent ABC transporter |
| XAC_RS07175 | XAC1403 | -1.29273 | hypothetical protein |
| XAC_RS24575 | - | -1.83632 | hypothetical protein |
| XAC_RS22925 | - | -1.48688 | prolyl oligopeptidase |
| XAC_RS22530 | - | -2.03094 | IS3 family transposase |
| XAC_RS02810 | XAC0539 | -1.54748 | oxidoreductase |
| XAC_RS12510 | XAC2460 | -1.01441 | glutathione S-transferase family protein |
| XAC_RS05495 | - | -1.72264 | PAS domain-containing sensor histidine kinase |
| recD | XAC4335 | -1.69098 | exodeoxyribonuclease V subunit alpha |
| XAC_RS05525 | XAC1081 | 1.27673 | HU family DNA-binding protein |
| XAC_RS12410 | - | -1.74871 | hypothetical protein |
| XAC_RS05175 | XAC1012 | 4.96518 | membrane protein |
| XAC_RS19195 | XAC3802 | 2.56231 | LysM peptidoglycan-binding domain-containing protein |
| XAC_RS20240 | XAC4018 | -1.50067 | bifunctional biotin--[acetyl-CoA-carboxylase |
| XAC_RS01230 | XAC0236 | -2.01671 | YkgJ family cysteine cluster protein |
| lldD | XAC0133 | -1.40105 | alpha-hydroxy-acid oxidizing enzyme |
| XAC_RS04345 | XAC0845 | -1.79545 | sigma-54-dependent Fis family transcriptional regulator |
| XAC_RS11880 | XAC2333 | -1.66811 | DUF1294 domain-containing protein |
| XAC_RS21345 | XAC4231 | -1.65668 | glucan 1,4-alpha-glucosidase |
| XAC_RS16180 | XAC3192 | -1.591 | hypothetical protein |
| XAC_RS24775 | XACb0049 | -1.98022 | hypothetical protein |
| XAC_RS20365 | XAC4042 | 1.42672 | hypothetical protein |
| XAC_RS12915 | XAC2536 | -1.18513 | hypothetical protein |
| XAC_RS21005 | XAC4169 | 2.64102 | GlsB/YeaQ/YmgE family stress response membrane protein |
| XAC_RS15665 | XAC3087 | 1.18303 | RebB protein |
| XAC_RS08775 | XAC1722 | -1.15875 | 2-C-methyl-D-erythritol 2,4-cyclodiphosphate synthase |
| XAC_RS06220 | XAC1219 | -1.56306 | phosphatase PAP2 family protein |
| XAC_RS21980 | XAC4359 | -1.50401 | CdaR family transcriptional regulator |
| XAC_RS15850 | XAC3126 | 3.13647 | response regulator |
| XAC_RS19840 | XAC3931 | -1.66201 | ATP-binding protein |
| XAC_RS01590 | XAC0304 | 1.9775 | hypothetical protein |
| XAC_RS03380 | XAC0651 | -1.69907 | hypothetical protein |
| XAC_RS08150 | XAC1602 | -1.50022 | FUSC family protein |
| XAC_RS02330 | XAC0445 | 1.07319 | alpha-ketoacid dehydrogenase subunit beta |
| XAC_RS14935 | XAC2942 | -1.35368 | PKHD-type hydroxylase |
| traD | - | -1.83333 | conjugative coupling factor TraD, PFGI-1 class |
| XAC_RS24770 | - | -1.36005 | hypothetical protein |
| XAC_RS10295 | XAC2024 | -1.77147 | TonB-dependent receptor |
| XAC_RS00350 | XAC0068 | -1.54953 | hypothetical protein |
| XAC_RS08470 | XAC1664 | -1.67425 | site-specific integrase |
| XAC_RS07140 | XAC1396 | -1.57006 | hypothetical protein |
| XAC_RS13420 | XAC2638 | -1.01669 | hypothetical protein |
| hppA | XAC3440 | -1.57093 | K+-insensitive pyrophosphate-energized proton pump |
| XAC_RS09340 | XAC1837 | -1.245 | hypothetical protein |
| XAC_RS01200 | XAC0230 | -1.20315 | tRNA (cytidine(34)-2'-O)-methyltransferase |
| XAC_RS16250 | XAC3206 | -1.20172 | hypothetical protein |
| XAC_RS02720 | XAC0522 | -1.76667 | Fis family transcriptional regulator |
| XAC_RS21380 | XAC4238 | -1.66021 | aldehyde dehydrogenase |
| XAC_RS01385 | XAC0266 | -1.44491 | TetR/AcrR family transcriptional regulator |
| XAC_RS00945 | XAC0182 | -1.11673 | ABC transporter ATP-binding protein |
| XAC_RS19885 | XAC3943 | -1.16262 | IS3 family transposase |
| XAC_RS08680 | XAC1703 | -1.55112 | hypothetical protein |
| XAC_RS05145 | XAC1005 | 3.09841 | peptidylprolyl isomerase |
| XAC_RS14375 | XAC2831 | -1.79962 | peptidase S8 |
| XAC_RS05240 | XAC1024 | -1.68199 | phospholipase C, phosphocholine-specific |
| XAC_RS00445 | - | -1.76922 | hypothetical protein |
| XAC_RS02700 | XAC0518 | -1.51716 | TIGR04222 domain-containing membrane protein |
| XAC_RS13450 | XAC2645 | -1.76177 | terminase |
| XAC_RS02880 | XAC0552 | -1.73799 | peptidase S53 |
| XAC_RS03005 | XAC0578 | -1.63965 | IS3 family transposase |
| XAC_RS07235 | XAC1415 | -1.20328 | 1-deoxy-D-xylulose-5-phosphate reductoisomerase |
| XAC_RS02015 | XAC0382 | -1.26422 | lipid A hydroxylase LpxO |
| XAC_RS10380 | XAC2041 | 2.7043 | phosphoenolpyruvate synthase |
| XAC_RS00920 | XAC0177 | -1.50495 | patatin |
| XAC_RS00595 | XAC0115 | -1.67179 | hypothetical protein |
| XAC_RS11240 | XAC2212 | -1.78161 | DNA topoisomerase III |
| XAC_RS08340 | XAC1639 | -1.14365 | formimidoylglutamate deiminase |
| XAC_RS02740 | XAC0525 | -1.53462 | hypothetical protein |
| XAC_RS00560 | XAC0109 | -1.54592 | thioredoxin |
| XAC_RS02090 | XAC0398 | -2.31277 | hypothetical protein |
| XAC_RS01525 | XAC0292 | 2.23632 | hypothetical protein |
| XAC_RS23455 | XAC1911 | 1.97175 | hypothetical protein |
| XAC_RS07430 | XAC1453 | 2.07063 | N-acetyltransferase |
| XAC_RS08840 | XAC1735 | 1.27339 | RNA-binding protein Hfq |
| XAC_RS15000 | XAC2955 | -1.52659 | nucleotidyltransferase family protein |
| XAC_RS17655 | XAC3488 | -1.71289 | MFS transporter |
| XAC_RS14265 | XAC2809 | -1.46725 | EamA/RhaT family transporter |
| XAC_RS06190 | XAC1213 | -1.27272 | carboxylesterase family protein |
| XAC_RS17060 | XAC3367 | 1.21574 | murein L,D-transpeptidase |
| XAC_RS18580 | XAC3673 | -1.27982 | hybrid sensor histidine kinase/response regulator |
| XAC_RS03565 | XAC0688 | -1.03415 | KR domain-containing protein |
| XAC_RS12900 | XAC2533 | -1.53051 | glycosyl hydrolase family 43 |
| XAC_RS18755 | XAC3708 | -1.64466 | LysR family transcriptional regulator |
| XAC_RS11145 | XAC2192 | -1.17021 | DUF4880 domain-containing protein |
| XAC_RS02185 | - | -1.83048 | lytic transglycosylase |
| XAC_RS10915 | XAC2148 | -1.32564 | RND transporter |
| XAC_RS08710 | XAC1709 | -1.21083 | HlyC/CorC family transporter |
| XAC_RS15390 | XAC3031 | -1.61722 | hybrid sensor histidine kinase/response regulator |
| XAC_RS14350 | XAC2826 | -1.44348 | alcohol dehydrogenase |
| XAC_RS00400 | XAC0077 | -1.50289 | exonuclease |
| XAC_RS22390 | - | -1.00993 | hypothetical protein |
| XAC_RS14615 | XAC2879 | -1.45032 | OsmC family peroxiredoxin |
| XAC_RS16800 | XAC3316 | -1.31845 | RNA methyltransferase |
| XAC_RS19090 | XAC3780 | -1.2489 | hypothetical protein |
| XAC_RS04390 | XAC0854 | -1.62145 | acyl-CoA dehydrogenase |
| XAC_RS13160 | XAC2584 | -1.02679 | GumC protein |
| XAC_RS13500 | XAC2656 | -1.37973 | phage tail protein I |
| XAC_RS11420 | XAC2247 | -1.12587 | hypothetical protein |
| XAC_RS13110 | XAC2574 | -1.13001 | glycosyltransferase |
| XAC_RS05870 | XAC1150 | 1.27174 | peroxiredoxin |
| hrpA | XAC3122 | -1.35482 | ATP-dependent RNA helicase HrpA |
| XAC_RS19820 | XAC3927 | -1.65884 | kinase |
| XAC_RS02605 | XAC0501 | -1.78364 | hypothetical protein |
| XAC_RS13700 | XAC2700 | 2.14657 | NADH-quinone oxidoreductase subunit NuoE |
| XAC_RS12875 | XAC2528 | 1.43507 | molecular chaperone HtpG |
| XAC_RS07445 | XAC1456 | 2.33302 | M3 family peptidase |
| XAC_RS01355 | - | 4.77952 | putative modified peptide |
| XAC_RS03850 | XAC0744 | -1.53071 | transcriptional regulator NrdR |
| XAC_RS11575 | XAC2274 | -1.72628 | conjugative transfer ATPase |
| mdcB | XAC0565 | -1.55341 | triphosphoribosyl-dephospho-CoA synthase MdcB |
| XAC_RS22170 | XACa0026 | -2.50797 | hypothetical protein |
| XAC_RS20440 | XAC4058 | -1.64765 | beta-xylosidase |
| XAC_RS16120 | XAC3180 | -1.72419 | iron transporter |
| XAC_RS06780 | XAC1327 | -1.44184 | DNA repair protein RecO |
| XAC_RS08025 | XAC1579 | -1.77154 | porin |
| XAC_RS02705 | XAC0519 | -1.30964 | CDP-alcohol phosphatidyltransferase family protein |
| XAC_RS13545 | XAC2667 | 2.5596 | prepilin-type cleavage/methylation domain-containing protein |
| XAC_RS01360 | - | -1.59877 | putative peptide maturation dehydrogenase |
| XAC_RS00270 | XAC0053 | -1.03658 | methyltransferase |
| XAC_RS03195 | XAC0613 | -1.51258 | SulP family inorganic anion transporter |
| XAC_RS03595 | XAC0693 | -1.86942 | TonB-dependent receptor |
| XAC_RS11465 | XAC2255 | -1.44498 | TIGR03759 family integrating conjugative element protein |
| XAC_RS17560 | XAC3469 | -1.26581 | glycoside hydrolase family 92 protein |
| XAC_RS05430 | XAC1060 | -1.2041 | hypothetical protein |
| XAC_RS02125 | XAC0405 | -1.74432 | hypersensitivity response secretion protein hrcV |
| XAC_RS13585 | XAC2677 | -1.4313 | superoxide dismutase |
| XAC_RS04595 | XAC0894 | -1.63716 | glutathione S-transferase |
| XAC_RS16395 | XAC3233 | -1.92515 | DDE transposase |
| XAC_RS14495 | XAC2855 | -1.59088 | two-component system response regulator CreB |
| XAC_RS18255 | XAC3609 | 1.21134 | fumarylacetoacetate hydrolase |
| XAC_RS13505 | - | -1.78044 | hypothetical protein |
| XAC_RS00380 | XAC0073 | -1.6327 | DUF1868 domain-containing protein |
| rplK | XAC0961 | 3.57539 | 50S ribosomal protein L11 |
| XAC_RS01175 | XAC0225 | -1.43226 | sensor histidine kinase |
| XAC_RS10500 | XAC2065 | -1.19227 | AcrB/AcrD/AcrF family protein |
| XAC_RS16080 | XAC3172 | -1.51316 | hypothetical protein |
| XAC_RS00090 | XAC0017 | -1.41509 | radical SAM protein |
| XAC_RS05160 | XAC1008 | -1.04198 | cell wall hydrolase |
| XAC_RS19395 | XAC3842 | -1.72414 | type I-C CRISPR-associated endonuclease Cas1 |
| XAC_RS09275 | XAC1824 | -1.67693 | hypothetical protein |
| XAC_RS11590 | XAC2277 | -1.62689 | NAD(P)-dependent oxidoreductase |
| XAC_RS14705 | XAC2897 | -1.62163 | GGDEF domain-containing protein |
| XAC_RS20340 | XAC4038 | -1.5659 | LysR family transcriptional regulator |
| XAC_RS02690 | XAC0516 | -1.34538 | ser/threonine protein phosphatase |
| XAC_RS16235 | XAC3203 | -1.48132 | glutathione S-transferase |
| XAC_RS06350 | XAC1244 | -1.11761 | enoyl-CoA hydratase/isomerase family protein |
| XAC_RS15010 | XAC2957 | -1.09987 | AI-2E family transporter |
| XAC_RS19285 | XAC3820 | -1.27994 | NYN domain-containing protein |
| XAC_RS05865 | XAC1149 | 3.27177 | bacterioferritin |
| XAC_RS12670 | XAC2491 | -1.30023 | DUF445 domain-containing protein |
| XAC_RS09415 | XAC1852 | -1.45413 | VOC family protein |
| XAC_RS00060 | XAC0011 | 2.36405 | biopolymer transporter ExbD |
| XAC_RS12500 | XAC2458 | -1.34927 | transglycosylase |
| XAC_RS20835 | XAC4136 | -1.72138 | hypothetical protein |
| XAC_RS15060 | XAC2967 | -1.33257 | KpsF/GutQ family sugar-phosphate isomerase |
| XAC_RS22260 | - | 1.10114 | hypothetical protein |
| XAC_RS05000 | XAC0977 | 2.26921 | 50S ribosomal protein L22 |
| XAC_RS05225 | XAC1022 | 1.32735 | L-threonine 3-dehydrogenase |
| XAC_RS17770 | XAC3515 | -1.58074 | cellulose synthase operon protein C |
| XAC_RS10270 | XAC2019 | 1.31273 | membrane protein |
| ppk2 | XAC0941 | 1.82103 | polyphosphate kinase 2 |
| XAC_RS24720 | XACa0032 | -1.515 | YecA family protein |
| XAC_RS14230 | XAC2803 | -1.29533 | DNA-binding response regulator |
| XAC_RS06505 | XAC1275 | -1.53282 | glycoside hydrolase family 43 protein |
| XAC_RS07585 | XAC1482 | -1.65213 | MexE family multidrug efflux RND transporter periplasmic adaptor subunit |
| XAC_RS14460 | XAC2848 | -1.07576 | DUF3616 domain-containing protein |
| XAC_RS06515 | XAC1277 | -1.13924 | thioredoxin TrxC |
| XAC_RS18355 | XAC3630 | -1.49145 | copper resistance system multicopper oxidase |
| XAC_RS14835 | XAC2922 | -1.55877 | DNA-binding protein |
| XAC_RS20790 | - | -2.51139 | type VI secretion protein |
| XAC_RS18400 | XAC3639 | -1.21331 | ABC transporter ATP-binding protein |
| XAC_RS21485 | XAC4258 | -1.61469 | alpha-N-arabinofuranosidase |
| XAC_RS14515 | XAC2859 | -1.53725 | hypothetical protein |
| XAC_RS09265 | XAC1822 | -1.42833 | EthD family reductase |
| XAC_RS05560 | XAC1088 | -1.05474 | hypothetical protein |
| XAC_RS04995 | XAC0976 | 1.94429 | 30S ribosomal protein S19 |
| XAC_RS03375 | XAC0650 | -1.08353 | hypothetical protein |
| XAC_RS17580 | XAC3473 | -1.66508 | hybrid sensor histidine kinase/response regulator |
| XAC_RS13305 | XAC2615 | 4.28779 | hypothetical protein |
| cas7c | XAC3840 | -1.52468 | type I-C CRISPR-associated protein Cas7/Csd2 |
| XAC_RS18565 | XAC3670 | -1.5447 | EamA/RhaT family transporter |
| XAC_RS16005 | XAC3157 | -1.52079 | MFS transporter |
| XAC_RS02850 | XAC0547 | -1.61514 | hypothetical protein |
| XAC_RS05150 | XAC1006 | 2.62614 | malate dehydrogenase |
| XAC_RS08660 | XAC1699 | -1.49371 | glycosyltransferase family 1 protein |
| XAC_RS12565 | XAC2470 | -1.61169 | putrescine ABC transporter permease PotI |
| mviN | XAC1252 | -1.439 | murein biosynthesis integral membrane protein MurJ |
| XAC_RS19765 | XAC3918 | -1.13678 | hypothetical protein |
| XAC_RS12610 | XAC2479 | -1.51176 | gamma-glutamyl-gamma-aminobutyrate hydrolase |
| XAC_RS00190 | XAC0036 | 4.01696 | hypothetical protein |
| XAC_RS06225 | XAC1220 | -1.51 | glycosyltransferase family 39 protein |
| XAC_RS17590 | XAC3475 | -1.36146 | molybdenum ABC transporter substrate-binding protein |
| XAC_RS23680 | - | -2.14385 | relaxase |
| XAC_RS11235 | XAC2211 | -2.11691 | single-stranded DNA-binding protein |
| XAC_RS05465 | XAC1068 | -1.84999 | hypothetical protein |
| XAC_RS11060 | XAC2176 | -1.64566 | XRE family transcriptional regulator |
| XAC_RS15960 | XAC3148 | -1.09258 | potassium transporter Kup |
| XAC_RS20870 | XAC4144 | -1.17936 | hypothetical protein |
| XAC_RS00420 | XAC0081 | -2.50673 | type II toxin-antitoxin system RelE/ParE family toxin |
| XAC_RS03550 | XAC0684 | 1.36764 | response regulator |
| XAC_RS02110 | XAC0402 | -1.84558 | EscR/YscR/HrcR family type III secretion system export apparatus protein |
| XAC_RS19500 | XAC3863 | -1.16261 | transpeptidase |
| XAC_RS01610 | XAC0308 | -1.15515 | adenosine deaminase |
| XAC_RS15980 | XAC3152 | -1.68963 | hypothetical protein |
| XAC_RS06270 | XAC1228 | -1.59887 | sensor histidine kinase |
| XAC_RS06475 | XAC1269 | 1.15396 | STAS domain-containing protein |
| XAC_RS21095 | XAC4184 | -1.36183 | aldo/keto reductase |
| XAC_RS14430 | XAC2842 | -1.59906 | multidrug transporter |
| XAC_RS13885 | XAC2734 | -1.26374 | transcription elongation factor GreB |
| XAC_RS19495 | XAC3862 | -1.39957 | dipeptide epimerase |
| XAC_RS04385 | - | -1.56649 | hypothetical protein |
| XAC_RS07680 | XAC1509 | -1.08264 | DUF4209 domain-containing protein |
| phaR | XAC2402 | 2.16179 | polyhydroxyalkanoate synthesis repressor PhaR |
| XAC_RS20375 | XAC4046 | -1.34236 | S9 family peptidase |
| XAC_RS02865 | XAC0550 | -1.488 | [glutamate--ammonia-ligase |
| XAC_RS17600 | XAC3477 | -1.64463 | aldo/keto reductase |
| XAC_RS09490 | XAC1866 | -1.43887 | RHS repeat protein |
| XAC_RS20700 | XAC4108 | -1.53766 | hypothetical protein |
| XAC_RS10505 | XAC2066 | -1.25848 | AcrB/AcrD/AcrF family protein |
| XAC_RS17660 | XAC3489 | -1.59798 | TonB-denpendent receptor |
| XAC_RS14865 | XAC2928 | -1.25591 | M23 family peptidase |
| XAC_RS17910 | XAC3543 | -1.54189 | type II secretion system F family protein |
| XAC_RS16740 | XAC3305 | -1.41459 | Rieske (2Fe-2S) protein |
| XAC_RS17610 | XAC3479 | -1.11324 | hypothetical protein |
| XAC_RS08985 | XAC1764 | -1.04141 | SMP-30/gluconolactonase/LRE family protein |
| XAC_RS04350 | XAC0846 | -1.76948 | monooxygenase |
| XAC_RS16890 | XAC3332 | -1.81813 | phosphoadenosine phosphosulfate reductase |
| XAC_RS15150 | XAC2985 | -1.12258 | amino acid permease |
| XAC_RS14225 | XAC2802 | -1.652 | outer membrane channel protein |
| XAC_RS17775 | XAC3516 | -1.50494 | cellulase |
| XAC_RS20625 | XAC4093 | -1.37825 | membrane protein |
| XAC_RS21930 | XAC4349 | -1.60258 | zinc-binding alcohol dehydrogenase family protein |
| XAC_RS10760 | XAC2118 | -1.62678 | hypothetical protein |
| XAC_RS18225 | XAC3604 | 2.11793 | DUF465 domain-containing protein |
| XAC_RS19175 | XAC3798 | -1.60103 | glycosyltransferase family 39 protein |
| XAC_RS21355 | XAC4233 | -1.72023 | glyoxalase/bleomycin resistance/extradiol dioxygenase family protein |
| XAC_RS21335 | XAC4229 | -1.70679 | D-galactonate dehydratase family protein |
| XAC_RS04095 | XAC0795 | -1.65249 | protease |
| XAC_RS20725 | XAC4113 | -1.7166 | filamentous hemagglutinin N-terminal domain-containing protein |
| XAC_RS02870 | XAC0551 | -1.62337 | membrane protein |
| XAC_RS10190 | XAC2004 | -1.17047 | N-acetyltransferase |
| XAC_RS06235 | XAC1222 | -1.24136 | sensor histidine kinase |
| XAC_RS14605 | XAC2877 | -1.0284 | pirin family protein |
| XAC_RS01805 | XAC0342 | -1.64466 | IS3 family transposase |
| XAC_RS22310 | XACb0015 | -1.84048 | avirulence protein |
| XAC_RS21715 | XAC4306 | -1.70773 | RND transporter |
| XAC_RS10785 | XAC2123 | -1.30489 | PIG-L family deacetylase |
| XAC_RS07025 | XAC1373 | -1.44836 | DUF1295 domain-containing protein |
| XAC_RS13810 | XAC2720 | -1.11415 | tRNA pseudouridine(38-40) synthase TruA |
| XAC_RS08540 | XAC1676 | -1.59308 | heme lyase CcmF/NrfE family subunit |
| dnaE2 | XAC1199 | -1.63875 | error-prone DNA polymerase |
| XAC_RS03025 | XAC0582 | 1.93317 | hypothetical protein |
| XAC_RS01625 | XAC0310 | -1.70859 | oxidoreductase |
| XAC_RS22485 | XACb0052 | -1.86008 | partition gene repressor |
| XAC_RS02960 | XAC0568 | -1.44655 | GntR family transcriptional regulator |
| XAC_RS08910 | XAC1749 | -1.30132 | membrane protein |
| mlaD | XAC4341 | 1.21336 | outer membrane lipid asymmetry maintenance protein MlaD |
| XAC_RS04760 | XAC0928 | -1.45737 | peptidase S8 |
| XAC_RS05715 | XAC1117 | -1.44637 | hypothetical protein |
| XAC_RS11210 | XAC2206 | -1.79527 | hypothetical protein |
| XAC_RS16210 | XAC3198 | -1.59483 | aliphatic sulfonate ABC transporter substrate-binding protein |
| XAC_RS07350 | - | -1.59669 | penicillin acylase |
| XAC_RS16230 | XAC3202 | -1.40886 | GIY-YIG nuclease family protein |
| lptG | XAC3554 | -1.32461 | LPS export ABC transporter permease LptG |
| XAC_RS07625 | XAC1492 | 3.28918 | hypothetical protein |
| XAC_RS06470 | XAC1268 | -1.28741 | hypothetical protein |
| mpl | XAC3436 | -1.15096 | UDP-N-acetylmuramate:L-alanyl-gamma-D-glutamyl-meso-diaminopimelate ligase |
| XAC_RS21165 | XAC4196 | -1.47677 | MFS transporter |
| XAC_RS02165 | XAC0413 | -1.19791 | type III secretion protein HrpB7 |
| XAC_RS18140 | XAC3588 | -1.52098 | TIGR00374 family protein |
| XAC_RS10735 | XAC2113 | -1.13008 | DUF3300 domain-containing protein |
| XAC_RS21075 | XAC4182 | -1.71069 | cytochrome c biogenesis protein |
| XAC_RS23930 | - | -1.79769e+308 | hypothetical protein |
| XAC_RS10910 | XAC2147 | -1.5066 | CusA/CzcA family heavy metal efflux RND transporter |
| XAC_RS00865 | XAC0165 | -1.6702 | beta-xylosidase |
| XAC_RS18735 | XAC3704 | -1.74819 | uracil-DNA glycosylase |
| XAC_RS23105 | XAC1067 | -1.71756 | hypothetical protein |
| XAC_RS24380 | XAC3874 | -1.21239 | hypothetical protein |
| XAC_RS11355 | XAC2234 | -1.93636 | MFS transporter |
| XAC_RS24685 | XACa0006 | -1.60699 | recombinase family protein |
| XAC_RS24010 | - | -2.35541 | hypothetical protein |
| XAC_RS01340 | XAC0257 | -1.50576 | isocitrate lyase |
| XAC_RS03155 | XAC0606 | -1.44299 | endonuclease |
| XAC_RS08110 | XAC1595 | -1.01892 | NAD(+) kinase |
| XAC_RS01595 | XAC0305 | -1.47622 | gamma-glutamyltransferase family protein |
| XAC_RS14535 | XAC2863 | -1.67036 | type I toxin-antitoxin system SymE family toxin |
| XAC_RS15040 | XAC2963 | 1.30205 | DUF3108 domain-containing protein |
| XAC_RS17390 | XAC3435 | -1.42754 | hypothetical protein |
| XAC_RS00215 | XAC0042 | -1.36178 | glycosyl transferase |
| XAC_RS07345 | - | -2.77578 | hypothetical protein |
| XAC_RS20765 | XAC4121 | -1.85131 | type VI secretion system baseplate subunit TssK |
| fliR | XAC1941 | -1.87828 | flagellar biosynthetic protein FliR |
| XAC_RS20095 | XAC3989 | -1.63503 | RNA polymerase sigma factor |
| XAC_RS11315 | XAC2226 | -1.38139 | threonine transporter |
| XAC_RS24675 | XACa0003 | 1.14099 | hypothetical protein |
| XAC_RS11555 | - | -1.63346 | TIGR03746 family integrating conjugative element protein |
| XAC_RS02980 | XAC0572 | -1.60262 | HAMP domain-containing protein |
| XAC_RS08650 | XAC1697 | -1.52544 | amidohydrolase |
| XAC_RS20335 | XAC4037 | -2.13234 | DNA/RNA non-specific endonuclease |
| XAC_RS04740 | XAC0924 | -1.33236 | NADP transhydrogenase subunit alpha |
| XAC_RS15215 | XAC2998 | -1.5288 | TonB-dependent receptor |
| prmC | XAC0908 | -1.65858 | peptide chain release factor N(5)-glutamine methyltransferase |
| XAC_RS19190 | XAC3801 | 1.35881 | peptide deformylase |
| XAC_RS05730 | XAC1120 | -1.25234 | septum formation inhibitor Maf |
| XAC_RS13165 | XAC2585 | -1.33056 | GumB protein |
| XAC_RS17630 | XAC3483 | -1.5189 | DNA-binding response regulator |
| XAC_RS08590 | XAC1685 | -1.62006 | hypothetical protein |
| XAC_RS02975 | XAC0571 | -1.20515 | ATP-binding protein |
| XAC_RS20345 | XAC4039 | -1.77456 | membrane protein |
| XAC_RS08560 | XAC1680 | -1.54069 | serine protease |
| XAC_RS01040 | - | -2.81809 | hypothetical protein |
| XAC_RS12240 | XAC2407 | -1.47902 | tRNA (adenosine(37)-N6)-threonylcarbamoyltransferase complex ATPase subunit type 1 TsaE |
| XAC_RS13290 | XAC2612 | 1.2437 | VirB6 protein |
| XAC_RS02460 | XAC0473 | -1.58704 | porin family protein |
| XAC_RS00525 | XAC0103 | -1.8947 | hypothetical protein |
| XAC_RS14080 | XAC2773 | -1.03153 | TonB-dependent Receptor Plug domain protein |
| XAC_RS00795 | XAC0151 | -1.87707 | hypothetical protein |
| XAC_RS03515 | XAC0677 | 1.26619 | DUF2242 domain-containing protein |
| XAC_RS11470 | XAC2256 | -1.73459 | lytic transglycosylase |
| XAC_RS17260 | XAC3407 | 1.79769e+308 | TIGR02449 family protein |
| XAC_RS06075 | XAC1190 | -1.57177 | DUF763 domain-containing protein |
| XAC_RS16465 | XAC3245 | -1.33517 | RHS repeat protein |
| XAC_RS16025 | XAC3162 | 2.92109 | class A beta-lactamase |
| XAC_RS14945 | XAC2944 | -1.60799 | membrane protein |
| XAC_RS00250 | XAC0049 | -1.54893 | hypothetical protein |
| XAC_RS20875 | XAC4145 | -2.19896 | hypothetical protein |
| XAC_RS04245 | - | -1.98387 | hypothetical protein |
| XAC_RS23840 | - | -1.58734 | hypothetical protein |
| XAC_RS00615 | XAC0119 | -1.25864 | DUF4159 domain-containing protein |
| efp | XAC2380 | 2.77228 | elongation factor P |
| tsaB | XAC3106 | -1.01724 | tRNA (adenosine(37)-N6)-threonylcarbamoyltransferase complex dimerization subunit type 1 TsaB |
| XAC_RS05825 | XAC1140 | 2.18861 | type II toxin-antitoxin system ParD family antitoxin |
| XAC_RS06640 | XAC1300 | -1.20712 | RNA-binding S4 domain-containing protein |
| XAC_RS00820 | XAC0156 | -1.15695 | 1,4-alpha-glucan branching protein GlgB |
| XAC_RS10310 | XAC2027 | -2.01621 | hypothetical protein |
| XAC_RS08570 | XAC1682 | -1.24204 | DNA-directed RNA polymerase sigma-70 factor |
| XAC_RS06080 | XAC1191 | -1.73507 | hypothetical protein |
| XAC_RS20885 | XAC4147 | -1.26913 | type VI secretion system contractile sheath small subunit |
| XAC_RS02785 | XAC0535 | -1.13587 | divalent-cation tolerance protein CutA |
| XAC_RS10615 | XAC2088 | -1.23827 | tetraacyldisaccharide 4'-kinase |
| XAC_RS20435 | - | -1.75208 | hypothetical protein |
| XAC_RS18640 | XAC3684 | -1.90509 | hypothetical protein |
| XAC_RS04310 | XAC0838 | 2.02162 | LemA family protein |
| XAC_RS21520 | XAC4269 | -1.05051 | nuclear receptor-binding factor-like protein |
| XAC_RS10305 | XAC2026 | -1.31929 | DUF4142 domain-containing protein |
| XAC_RS11585 | XAC2276 | -1.79394 | LysR family transcriptional regulator |
| XAC_RS17715 | XAC3501 | -1.05262 | hypothetical protein |
| XAC_RS02260 | XAC0432 | -1.4483 | nucleoside-diphosphate sugar epimerase |
| XAC_RS02510 | XAC0482 | -1.41176 | haloacid dehalogenase |
| XAC_RS22150 | XACa0022 | -1.79446 | avirulence protein |
| XAC_RS05235 | XAC1023 | -1.68959 | TonB-dependent receptor |
| XAC_RS01650 | XAC0315 | -1.9508 | hypothetical protein |
| XAC_RS12460 | XAC2450 | -1.48992 | ATP-dependent DNA helicase |
| relA | XAC3113 | -1.01762 | bifunctional (p)ppGpp synthetase/guanosine-3',5'-bis(diphosphate) 3'-pyrophosphohydrolase |
| XAC_RS21750 | XAC4313 | -2.07431 | type II toxin-antitoxin system RelE/ParE family toxin |
| hisH | XAC1832 | -1.91059 | imidazole glycerol phosphate synthase subunit HisH |
| XAC_RS11720 | XAC2301 | 1.56333 | hypothetical protein |
| XAC_RS15270 | - | 2.50564 | DUF2170 domain-containing protein |
| XAC_RS08610 | XAC1689 | -1.72472 | hypothetical protein |
| XAC_RS23600 | - | -1.30453 | DUF3742 domain-containing protein |
| XAC_RS13745 | XAC2709 | -1.11786 | group 1 truncated hemoglobin |
| XAC_RS03325 | XAC0641 | -1.23288 | HlyD family secretion protein |
| XAC_RS12660 | XAC2489 | -1.22135 | aspartate aminotransferase family protein |
| XAC_RS21995 | XAC4362 | -1.33887 | peptidase M19 |
| XAC_RS13150 | XAC2582 | -1.36028 | hypothetical protein |
| XAC_RS16520 | XAC3258 | -1.35787 | succinoglycan biosynthesis protein |
| XAC_RS00325 | XAC0063 | -1.5673 | aryl sulfotransferase |
| galE | XAC3740 | -1.60471 | UDP-glucose 4-epimerase GalE |
| XAC_RS10995 | XAC2164 | -1.27499 | DoxX family protein |
| XAC_RS15720 | XAC3099 | 1.69864 | methyl-accepting chemotaxis protein |
| XAC_RS23645 | XAC2373 | -1.0864 | pectate lyase |
| XAC_RS13950 | XAC2747 | 1.15287 | M13 family peptidase |
| XAC_RS14210 | XAC2799 | -1.60197 | AcrB/AcrD/AcrF family protein |
| XAC_RS24835 | - | -1.98328 | IS3 family transposase |
| XAC_RS18360 | XAC3631 | -1.54721 | copper resistance protein B |
| XAC_RS20720 | XAC4112 | -1.7212 | type VI secretion system protein TssA |
| XAC_RS10765 | - | -1.00123 | hypothetical protein |
| XAC_RS09170 | XAC1802 | -1.70589 | aspartate/glutamate racemase family protein |
| XAC_RS20830 | XAC4135 | -1.42333 | hypothetical protein |
| clpV | XAC4140 | -1.86282 | type VI secretion system ATPase TssH |
| XAC_RS08260 | XAC1622 | 2.42487 | 50S ribosomal protein L9 |
| XAC_RS21210 | XAC4206 | -1.79566 | hypothetical protein |
| XAC_RS13330 | XAC2620 | 2.83783 | hypothetical protein |
| XAC_RS11395 | XAC2242 | -1.5569 | O-methyltransferase |
| XAC_RS02390 | XAC0458 | -2.2702 | K+/H+ antiporter subunit F |
| rpsB | XAC1422 | 3.75998 | 30S ribosomal protein S2 |
| XAC_RS05980 | XAC1172 | -1.7917 | hypothetical protein |
| ubiB | XAC1738 | -1.552 | 2-polyprenylphenol 6-hydroxylase |
| XAC_RS05850 | XAC1146 | -1.74505 | TonB-dependent receptor |
| XAC_RS00095 | XAC0018 | -1.11593 | hypothetical protein |
| XAC_RS20510 | XAC4071 | -1.66177 | hypothetical protein |
| XAC_RS18905 | XAC3741 | -1.82799 | glycosyltransferase family 1 protein |
| XAC_RS20160 | XAC4002 | -1.04802 | hypothetical protein |
| XAC_RS01755 | XAC0336 | -1.24463 | methionine synthase |
| XAC_RS12305 | XAC2419 | -1.50778 | membrane protein |
| XAC_RS02150 | XAC0410 | -1.62641 | type III secretion protein HrpB4 |
| XAC_RS17130 | XAC3381 | 2.55871 | type IV pilus secretin PilQ |
| XAC_RS24530 | XAC4134 | -3.54406 | hypothetical protein |
| XAC_RS16035 | XAC3164 | -1.26148 | hypothetical protein |
| XAC_RS22135 | XACa0019 | -1.52856 | hypothetical protein |
| XAC_RS06715 | XAC1314 | 2.32387 | enoyl-CoA hydratase |
| XAC_RS12860 | XAC2525 | 2.91855 | hypothetical protein |
| XAC_RS21610 | XAC4286 | -1.2802 | formamidopyrimidine-DNA glycosylase |
| cobT | XAC3186 | -1.45297 | nicotinate-nucleotide--dimethylbenzimidazole phosphoribosyltransferase |
| XAC_RS13960 | XAC2749 | -1.57783 | methylated-DNA--protein-cysteine methyltransferase |
| XAC_RS01320 | XAC0253 | -1.52639 | AraC family transcriptional regulator |
| XAC_RS22665 | XAC3891 | -1.23846 | hypothetical protein |
| XAC_RS01430 | XAC0275 | -1.67038 | MerC domain-containing protein |
| XAC_RS07240 | XAC1416 | -1.41494 | phosphatidate cytidylyltransferase |
| bamB | XAC2020 | 1.50184 | outer membrane protein assembly factor BamB |
| XAC_RS09030 | XAC1772 | -1.58718 | glycoside hydrolase |
| XAC_RS01520 | XAC0291 | -1.81967 | TonB-dependent receptor |
| XAC_RS19960 | XAC3960 | -1.25683 | ferredoxin reductase |
| XAC_RS14650 | XAC2886 | -1.42069 | DUF819 domain-containing protein |
| lpdA | XAC3659 | 1.77311 | dihydrolipoyl dehydrogenase |
| XAC_RS21505 | XAC4264 | -2.20618 | hypothetical protein |
| XAC_RS09325 | XAC1834 | -1.20984 | imidazole glycerol phosphate synthase cyclase subunit |
| XAC_RS21970 | XAC4357 | -1.14836 | DUF2628 domain-containing protein |
| XAC_RS05165 | XAC1010 | -1.59154 | NADPH-dependent 2,4-dienoyl-CoA reductase |
| XAC_RS18700 | XAC3696 | -1.86672 | DUF4031 domain-containing protein |
| XAC_RS10595 | XAC2084 | -1.56872 | DNA internalization-related competence protein ComEC/Rec2 |
| XAC_RS19730 | - | -1.70881 | hypothetical protein |
| XAC_RS07530 | XAC1473 | -1.50067 | DUF3857 domain-containing protein |
| XAC_RS22295 | XACb0011 | -1.27332 | hypothetical protein |
| XAC_RS13020 | - | -1.67348 | hypothetical protein |
| XAC_RS00020 | XAC0003 | -1.71141 | DNA replication and repair protein RecF |
| XAC_RS21720 | XAC4307 | -1.59795 | FUSC family protein |
| ccmA | XAC2323 | -1.00155 | cytochrome c biogenesis ATP-binding export protein CcmA |
| XAC_RS21965 | XAC4355 | -1.30667 | glycoside hydrolase family 92 protein |
| XAC_RS11455 | XAC2253 | -2.19438 | PilL protein |
| xerD | XAC3551 | -1.50597 | site-specific tyrosine recombinase XerD |
| XAC_RS15835 | XAC3123 | 2.27 | DNA starvation/stationary phase protection protein |
| XAC_RS15435 | XAC3040 | -1.54798 | homoserine O-acetyltransferase |
| XAC_RS09460 | XAC1861 | 1.03661 | carbamoyl-phosphate synthase small subunit |
| XAC_RS01730 | XAC0331 | -1.28508 | methylenetetrahydrofolate reductase |
| mreD | XAC0658 | -1.57668 | rod shape-determining protein MreD |
| XAC_RS19155 | XAC3794 | -1.5427 | membrane protein |
| XAC_RS06375 | XAC1249 | 1.63768 | 50S ribosomal protein L27 |
| XAC_RS02735 | XAC0524 | -1.2769 | transcriptional regulator |
| XAC_RS20360 | XAC4041 | -2.4507 | hypothetical protein |
| XAC_RS10250 | XAC2015 | 3.51401 | nucleoside-diphosphate kinase |
| XAC_RS06165 | XAC1207 | -1.5337 | AraC family transcriptional regulator |
| XAC_RS12375 | XAC2434 | 2.9574 | hypothetical protein |
| XAC_RS15025 | XAC2960 | -1.77372 | hypothetical protein |
| XAC_RS05590 | XAC1094 | -1.33044 | lipopolysaccharide heptosyltransferase family protein |
| XAC_RS23945 | - | -1.79769e+308 | hypothetical protein |
| XAC_RS23435 | - | -1.54451 | hypothetical protein |
| XAC_RS16770 | XAC3310 | -1.53621 | LacI family DNA-binding transcriptional regulator |
| XAC_RS01560 | XAC0298 | -1.59144 | DUF3225 domain-containing protein |
| XAC_RS17450 | XAC3447 | -1.60588 | phosphomethylpyrimidine synthase |
| XAC_RS22030 | XAC4369 | -1.7419 | phosphatase PAP2 family protein |
| XAC_RS06985 | XAC1365 | -1.81483 | 2'-5' RNA ligase |
| clpA | XAC2001 | 1.37315 | ATP-dependent Clp protease ATP-binding subunit ClpA |
| XAC_RS11950 | XAC2347 | -1.08617 | N-acetyltransferase |
| XAC_RS04505 | XAC0879 | -2.21907 | protocatechuate 3,4-dioxygenase subunit alpha |
| XAC_RS10485 | XAC2062 | -1.35296 | cytochrome c biogenesis protein CcsA |
| XAC_RS17380 | XAC3433 | -1.40127 | phosphatase PAP2 family protein |
| XAC_RS11955 | XAC2348 | -1.08253 | acetylglutamate kinase |
| XAC_RS11595 | XAC2278 | -1.78305 | tripartite tricarboxylate transporter substrate binding protein |
| XAC_RS18625 | XAC3681 | -1.56415 | sorbosone dehydrogenase |
| XAC_RS02230 | XAC0426 | -1.02095 | 1,4-alpha-glucan branching enzyme |
| XAC_RS00530 | XAC0104 | -1.5481 | M4 family peptidase |
| XAC_RS07375 | XAC1442 | -1.62276 | AraC family transcriptional regulator |
| XAC_RS08505 | XAC1669 | -1.23745 | hybrid sensor histidine kinase/response regulator |
| XAC_RS01400 | XAC0269 | -1.78706 | DUF3325 domain-containing protein |
| XAC_RS18590 | XAC3675 | -1.08047 | DUF1415 domain-containing protein |
| XAC_RS23535 | - | -1.81278 | hypothetical protein |
| pilV | XAC2668 | 2.92609 | type IV pilus modification protein PilV |
| XAC_RS16755 | XAC3308 | 1.60318 | large conductance mechanosensitive channel protein MscL |
| XAC_RS17215 | XAC3398 | -1.26169 | glyoxalase |
| XAC_RS17690 | XAC3495 | -1.23035 | SGNH/GDSL hydrolase family protein |
| XAC_RS20820 | XAC4132 | -1.63933 | histidine-type phosphatase |
| XAC_RS20595 | XAC4088 | -1.2614 | glycosyltransferase family 2 protein |
| XAC_RS22735 | - | -2.00856 | hypothetical protein |
| XAC_RS12145 | XAC2388 | -1.10159 | barstar family protein |
| XAC_RS17605 | XAC3478 | -1.79699 | ketosteroid isomerase |
| XAC_RS17455 | XAC3448 | -1.85513 | TonB-dependent receptor |
| XAC_RS03505 | XAC0675 | -1.31226 | hypothetical protein |
| gspF | XAC0697 | -1.56862 | type II secretion system protein GspF |
| XAC_RS11115 | XAC2186 | -1.81698 | conjugal transfer protein TraG |
| XAC_RS20200 | XAC4010 | 2.0144 | hypothetical protein |
| XAC_RS24155 | XAC3380 | 4.46891 | hypothetical protein |
| XAC_RS11255 | XAC2215 | -1.04132 | hypothetical protein |
| XAC_RS19750 | XAC3915 | -1.81029 | JAB domain-containing protein |
| XAC_RS04010 | XAC0777 | -1.0065 | phospho-N-acetylmuramoyl-pentapeptide-transferase |
| XAC_RS06105 | XAC1196 | -1.49837 | LexA repressor 1 |
| XAC_RS10930 | XAC2151 | -1.69403 | outer membrane protein |
| XAC_RS15440 | XAC3041 | -1.55418 | M23 family peptidase |
| XAC_RS01330 | XAC0255 | -1.49275 | LysR family transcriptional regulator |
| XAC_RS02120 | XAC0404 | -1.98064 | type III secretion protein HpaP |
| XAC_RS18185 | XAC3597 | 1.58593 | phytanoyl-CoA dioxygenase family protein |
| XAC_RS14200 | XAC2797 | -1.19905 | ABC transporter ATP-binding protein |
| XAC_RS11550 | XAC2271 | -1.23931 | TIGR03750 family conjugal transfer protein |
| XAC_RS03065 | XAC0590 | -1.4224 | DUF3817 domain-containing protein |
| XAC_RS16330 | XAC3222 | -1.26278 | endonuclease domain-containing protein |
| XAC_RS21470 | XAC4255 | -1.62099 | MFS transporter |
| fliO | XAC1945 | -1.55975 | flagellar biosynthetic protein FliO |
| XAC_RS12035 | XAC2365 | -1.64497 | ethanolamine ammonia-lyase subunit EutB |
| yccS | XAC0510 | -1.47095 | TIGR01666 family membrane protein |
| XAC_RS10780 | - | -1.17156 | dehydrogenase |
| XAC_RS13985 | XAC2753 | -1.42082 | NlpC/P60 family protein |
| pgaB | XAC1812 | -1.53298 | poly-beta-1,6-N-acetyl-D-glucosamine N-deacetylase PgaB |
| XAC_RS04705 | XAC0917 | -1.62472 | TetR/AcrR family transcriptional regulator |
| XAC_RS03755 | XAC0726 | -1.57238 | MBL fold metallo-hydrolase |
| XAC_RS03760 | XAC0727 | -1.36451 | AraC family transcriptional regulator |
| XAC_RS15190 | XAC2993 | -1.14304 | hypothetical protein |
| XAC_RS16295 | XAC3214 | -1.08967 | thiol-disulfide oxidoreductase DCC family protein |
| XAC_RS20155 | XAC4003 | -1.18643 | drug/metabolite exporter YedA |
| mmsB | XAC1316 | 1.50323 | 3-hydroxyisobutyrate dehydrogenase |
| XAC_RS13445 | XAC2644 | -1.79039 | phage major capsid protein, P2 family |
| XAC_RS21925 | XAC4348 | -1.1372 | DNA recombination protein RmuC |
| XAC_RS11020 | XAC2169 | 2.03389 | HAMP domain-containing protein |
| XAC_RS05285 | - | -1.79769e+308 | hypothetical protein |
| XAC_RS02280 | XAC0435 | -1.47235 | hypothetical protein |
| XAC_RS17695 | XAC3496 | -1.31536 | methyltransferase domain-containing protein |
| XAC_RS18975 | - | -1.98491 | DUF2894 domain-containing protein |
| ssrS | - | 1.49274 | - |
| XAC_RS13970 | XAC2751 | -1.53608 | FAD-dependent oxidoreductase |
| XAC_RS02670 | XAC0512 | -1.13231 | hypothetical protein |
| XAC_RS10400 | XAC2045 | -1.27091 | 3-hydroxybutyrate dehydrogenase |
| XAC_RS21175 | XAC4199 | -1.61261 | polyvinylalcohol dehydrogenase |
| XAC_RS08555 | XAC1679 | -1.29022 | heme ABC transporter permease |
| XAC_RS02495 | XAC0479 | -1.56265 | flavin reductase family protein |
| XAC_RS09830 | XAC1931 | 2.49681 | chemotaxis protein |
| XAC_RS04850 | XAC0947 | -1.13421 | outer membrane lipoprotein LolB |
| XAC_RS11230 | - | -1.84798 | integrase |
| XAC_RS14145 | - | -1.35322 | hypothetical protein |
| XAC_RS13130 | XAC2578 | -1.44214 | GumI protein |
| XAC_RS17705 | XAC3499 | -1.16656 | polysaccharide deacetylase family protein |
| XAC_RS20050 | XAC3979 | 1.80337 | histidine biosynthesis protein HisIE |
| XAC_RS05735 | XAC1121 | 1.67801 | hypothetical protein |
| XAC_RS23285 | XAC1510 | -1.0345 | integrase |
| XAC_RS14520 | XAC2860 | -1.79113 | hypothetical protein |
| XAC_RS11505 | XAC2264 | -1.05233 | hypothetical protein |
| XAC_RS17930 | XAC3547 | -1.74453 | protease |
| XAC_RS20685 | XAC4105 | -1.18207 | AMP-ligase |
| phoU | XAC1573 | -1.44382 | phosphate transport system regulatory protein PhoU |
| XAC_RS17190 | XAC3393 | -1.16228 | bifunctional (p)ppGpp synthetase/guanosine-3',5'-bis(diphosphate) 3'-pyrophosphohydrolase |
| XAC_RS06925 | XAC1355 | -2.21123 | DksA/TraR family C4-type zinc finger protein |
| XAC_RS01080 | XAC0206 | -1.44359 | ammonium transporter |
| XAC_RS07480 | XAC1463 | 1.53766 | phospholipase |
| XAC_RS23440 | - | 1.14007 | hypothetical protein |
| XAC_RS15490 | XAC3051 | -1.04309 | sulfotransferase family protein |
| XAC_RS20235 | XAC4017 | -1.12462 | type III pantothenate kinase |
| XAC_RS17855 | XAC3532 | -1.41251 | UTRA domain-containing protein |
| XAC_RS18740 | XAC3705 | -1.04963 | putative DNA modification/repair radical SAM protein |
| XAC_RS09310 | XAC1831 | -1.27068 | bifunctional histidinol-phosphatase/imidazoleglycerol-phosphate dehydratase |
| XAC_RS14680 | - | -1.63725 | xanthine dehydrogenase |
| XAC_RS16990 | XAC3353 | -1.84453 | DUF4440 domain-containing protein |
| XAC_RS15370 | XAC3027 | -1.37677 | MFS transporter |
| fusA | XAC0969 | 2.73109 | elongation factor G |
| XAC_RS04540 | XAC0886 | -1.26885 | hypothetical protein |
| XAC_RS08535 | XAC1675 | -1.78144 | DsbE family thiol:disulfide interchange protein |
| XAC_RS14855 | XAC2926 | -1.1701 | pyrroline-5-carboxylate reductase |
| XAC_RS15110 | XAC2977 | 1.82933 | PTS fructose IIA component family protein |
| XAC_RS11360 | XAC2235 | -1.40169 | AraC family transcriptional regulator |
| recC | XAC4337 | -1.47369 | exodeoxyribonuclease V subunit gamma |
| thiD | XAC1753 | -1.05039 | bifunctional hydroxymethylpyrimidine kinase/phosphomethylpyrimidine kinase |
| XAC_RS11745 | XAC2306 | -1.51413 | endonuclease |
| XAC_RS02295 | - | -1.52846 | efflux transporter periplasmic adaptor subunit |
| trxB | XAC2005 | 1.18048 | thioredoxin-disulfide reductase |
| rseP | XAC1414 | -1.4964 | RIP metalloprotease RseP |
| XAC_RS20550 | - | -1.92245 | carbonic anhydrase |
| XAC_RS04610 | XAC0897 | -1.46721 | histidine kinase |
| XAC_RS06945 | XAC1357 | -1.22726 | heat-shock protein Hsp70 |
| XAC_RS19045 | XAC3769 | -1.01279 | DNA/RNA non-specific endonuclease |
| XAC_RS08035 | XAC1580 | -1.11581 | carbonic anhydrase |
| XAC_RS15815 | XAC3119 | -1.48193 | PhoPQ-regulated protein |
| XAC_RS04555 | XAC0889 | -1.0965 | 23S rRNA (cytidine(2498)-2'-O)-methyltransferase RlmM |
| lptA | XAC2970 | 1.40948 | lipopolysaccharide transport periplasmic protein LptA |
| gph | XAC3621 | -1.33537 | phosphoglycolate phosphatase |
| XAC_RS23955 | - | -1.85959 | hypothetical protein |
| rpsH | XAC0986 | 2.26151 | 30S ribosomal protein S8 |
| XAC_RS24325 | - | -3.09721 | hypothetical protein |
| XAC_RS21740 | XAC4311 | -1.40749 | hypothetical protein |
| XAC_RS19460 | XAC3855 | -1.12998 | hypothetical protein |
| XAC_RS06335 | XAC1241 | -1.12636 | hypothetical protein |
| XAC_RS04500 | XAC0878 | -1.51746 | protocatechuate 3,4-dioxygenase subunit beta |
| XAC_RS12600 | XAC2477 | -1.13074 | aspartate aminotransferase family protein |
| XAC_RS19055 | - | -1.61786 | LysR family transcriptional regulator |
| XAC_RS20495 | XAC4068 | -1.31152 | 2-dehydropantoate 2-reductase |
| XAC_RS13440 | XAC2643 | -1.64807 | phage capsid scaffolding protein |
| bioB | XAC0388 | -1.28475 | biotin synthase |
| XAC_RS21655 | XAC4295 | -1.50696 | tetracycline resistance MFS efflux pump |
| nagA | XAC0715 | -1.18636 | N-acetylglucosamine-6-phosphate deacetylase |
| XAC_RS20965 | XAC4162 | -1.2757 | TolC family protein |
| XAC_RS02585 | XAC0497 | -1.22109 | hypothetical protein |
| XAC_RS14405 | XAC2838 | -1.60765 | LysR family transcriptional regulator |
| XAC_RS18760 | XAC3709 | 1.16251 | flavodoxin family protein |
| XAC_RS00895 | XAC0172 | -1.62859 | hypothetical protein |
| XAC_RS04770 | XAC0930 | 1.24604 | peptidase S8 |
| XAC_RS16350 | XAC3224 | -1.66936 | hypothetical protein |
| XAC_RS04620 | XAC0899 | -1.99089 | DUF4440 domain-containing protein |
| XAC_RS18805 | XAC3719 | -1.65429 | DNA-binding response regulator |
| XAC_RS02070 | XAC0394 | -2.04081 | HrpF protein |
| XAC_RS20300 | - | -1.11353 | catalase |
| XAC_RS17485 | XAC3454 | -1.40825 | threonine/serine dehydratase |
| XAC_RS11200 | XAC2205 | -1.61935 | ParA family protein |
| XAC_RS23610 | XAC2199 | -2.76032 | hypothetical protein |
| XAC_RS19950 | XAC3958 | -1.57518 | EAL domain-containing protein |
| XAC_RS15350 | XAC3023 | -1.63987 | DUF1906 domain-containing protein |
| nusA | XAC2688 | 1.25223 | transcription termination/antitermination protein NusA |
| XAC_RS24865 | - | -1.79769e+308 | hypothetical protein |
| XAC_RS13785 | XAC2716 | -1.17594 | tryptophan synthase subunit alpha |
| phoR | XAC1041 | -1.65027 | phosphate regulon sensor histidine kinase PhoR |
| XAC_RS12570 | XAC2471 | -1.57984 | putrescine ABC transporter permease |
| XAC_RS07785 | XAC1529 | -1.49634 | hydrogen peroxide-inducible genes activator |
| XAC_RS01310 | XAC0251 | -1.18226 | TetR/AcrR family transcriptional regulator |
| XAC_RS00280 | XAC0055 | -1.49005 | hypothetical protein |
| XAC_RS10730 | XAC2112 | -1.17888 | 23S rRNA pseudouridine(955/2504/2580) synthase |
| XAC_RS21955 | XAC4353 | -1.41144 | hypothetical protein |
| XAC_RS16140 | XAC3184 | -1.63339 | adenosylcobinamide-GDP ribazoletransferase |
| XAC_RS04400 | XAC0856 | -1.61711 | ABC transporter oligopeptide-binding protein |
| XAC_RS04410 | XAC0858 | -1.72058 | ABC transporter permease |
| XAC_RS23705 | - | -1.79769e+308 | hypothetical protein |
| XAC_RS00335 | XAC0065 | 1.55308 | phage tail protein |
| XAC_RS07085 | XAC1385 | -1.36303 | HAD family hydrolase |
| XAC_RS05350 | XAC1046 | 2.82664 | isocitrate dehydrogenase |
| XAC_RS14260 | XAC2808 | -1.04576 | DUF72 domain-containing protein |
| XAC_RS08675 | XAC1702 | -1.88137 | radical SAM protein |
| kdpB | XAC0757 | -1.78093 | potassium-transporting ATPase subunit B |
| XAC_RS21585 | XAC4281 | -1.17766 | ATP-dependent DNA helicase Rep |
| XAC_RS05740 | XAC1122 | 2.74489 | 50S ribosomal protein L32 |
| XAC_RS20205 | XAC4011 | 3.12138 | hypothetical protein |
| XAC_RS21325 | XAC4227 | -1.69802 | alpha-glucuronidase |
| XAC_RS18010 | XAC3562 | -1.63367 | pectate lyase |
| XAC_RS00410 | XAC0079 | -1.3722 | hemolysin III |
| XAC_RS19050 | XAC3770 | -1.52786 | alpha/beta hydrolase |
| XAC_RS16805 | XAC3317 | -1.06883 | N-acetyltransferase |
| XAC_RS02520 | XAC0484 | 2.02871 | S-adenosylmethionine decarboxylase proenzyme |
| XAC_RS12620 | XAC2481 | -1.36708 | FAD-binding oxidoreductase |
| XAC_RS19010 | XAC3762 | -1.67221 | enamine deaminase RidA |
| XAC_RS21500 | XAC4262 | 3.8845 | hypothetical protein |
| XAC_RS19815 | XAC3926 | -1.09406 | hypothetical protein |
| XAC_RS11180 | XAC2200 | -1.8568 | hypothetical protein |
| XAC_RS17145 | XAC3384 | 2.27247 | fimbrial protein |
| XAC_RS08585 | XAC1684 | -1.31151 | cytochrome c |
| XAC_RS01410 | - | -1.50447 | DUF3649 domain-containing protein |
| XAC_RS20460 | XAC4062 | -1.74458 | TonB-dependent siderophore receptor |
| XAC_RS14805 | XAC2917 | -1.20953 | Holliday junction resolvase RuvX |
| XAC_RS16015 | XAC3160 | -1.63123 | phospholipase C, phosphocholine-specific |
| XAC_RS07170 | XAC1402 | -1.33044 | membrane protein |
| XAC_RS10775 | XAC2121 | -1.28329 | O-methyltransferase |
| XAC_RS05400 | - | 1.6852 | hypothetical protein |
| XAC_RS13070 | XAC2566 | -1.49521 | glycosyl transferase |
| XAC_RS22145 | XACa0021 | -1.83368 | replication protein A |
| XAC_RS00995 | XAC0191 | -1.49284 | AEC family transporter |
| XAC_RS04745 | XAC0925 | -1.02344 | hypothetical protein |
| XAC_RS17035 | XAC3362 | -1.75205 | cell envelope biogenesis protein TonB |
| XAC_RS02145 | XAC0409 | -1.73278 | EscJ/YscJ/HrcJ family type III secretion inner membrane ring protein |
| XAC_RS21195 | XAC4203 | -1.35847 | translocation/assembly module TamB |
| XAC_RS08785 | XAC1724 | -1.30444 | SMR domain protein |
| XAC_RS24550 | - | -1.80911 | hypothetical protein |
| XAC_RS14630 | XAC2882 | -3.72249 | putative selenoprotein |
| XAC_RS14500 | XAC2856 | -1.02381 | 23S rRNA (adenine(2030)-N(6))-methyltransferase RlmJ |
| raiA | XAC2973 | 1.23969 | ribosomal subunit interface protein |
| XAC_RS14040 | XAC2765 | -1.25957 | metalloprotease PmbA |
| XAC_RS21170 | XAC4197 | -1.46749 | gluconokinase |
| XAC_RS02570 | XAC0494 | -1.36238 | hybrid sensor histidine kinase/response regulator |
| XAC_RS13095 | XAC2571 | -1.39495 | ketoacyl-ACP synthase III |
| XAC_RS08200 | - | -1.40764 | hypothetical protein |
| XAC_RS12635 | XAC2484 | -1.50819 | MFS transporter |
| mrdA | XAC0659 | -1.67653 | penicillin-binding protein 2 |
| XAC_RS20600 | XAC4089 | -1.03358 | NAD(P)/FAD-dependent oxidoreductase |
| mmsA | XAC1312 | 2.03088 | methylmalonate-semialdehyde dehydrogenase (CoA acylating) |
| XAC_RS01635 | XAC0312 | -1.51828 | LysR family transcriptional regulator |
| XAC_RS04935 | XAC0964 | 4.36142 | 50S ribosomal protein L7/L12 |
| XAC_RS07335 | XAC1434 | 1.76953 | right-handed parallel beta-helix repeat-containing protein |
| XAC_RS21115 | XAC4188 | -1.40449 | L-fuconate dehydratase |
| XAC_RS22400 | XACb0036 | -1.39042 | lytic transglycosylase |
| XAC_RS15455 | XAC3044 | -1.27324 | AsmA family protein |
| XAC_RS17850 | XAC3531 | -1.66526 | nicotinamide riboside transporter PnuC |
| XAC_RS08105 | XAC1594 | -1.18868 | ribosomal RNA large subunit methyltransferase K/L |
| XAC_RS14290 | XAC2814 | -1.64311 | sigma-70 family RNA polymerase sigma factor |
| XAC_RS00745 | XAC0143 | -1.13836 | sugar kinase |
| XAC_RS11435 | XAC2249 | -1.83445 | hypothetical protein |
| XAC_RS20145 | XAC3999 | -1.58539 | alpha/beta hydrolase |
| xerC | XAC0636 | -1.4781 | tyrosine recombinase XerC |
| XAC_RS20315 | XAC4033 | -1.88304 | hypothetical protein |
| XAC_RS04085 | - | -1.60108 | hypothetical protein |
| XAC_RS15160 | XAC2987 | -1.86815 | amino acid amidase |
| XAC_RS09815 | XAC1928 | -1.72195 | hypothetical protein |
| XAC_RS23825 | - | -1.63614 | hypothetical protein |
| XAC_RS01425 | XAC0274 | -1.08242 | nuclease |
| XAC_RS03480 | XAC0670 | -1.46627 | sulfite exporter TauE/SafE family protein |
| XAC_RS17150 | XAC3385 | 2.70846 | pilus assembly protein PilM |
| XAC_RS08725 | XAC1712 | -1.11941 | YdcF family protein |
| XAC_RS08995 | XAC1766 | -1.47915 | 2-dehydro-3-deoxy-6-phosphogalactonate aldolase |
| XAC_RS06810 | XAC1333 | -1.59067 | deoxyribodipyrimidine photo-lyase |
| XAC_RS02035 | XAC0386 | -1.60707 | hypothetical protein |
| XAC_RS19115 | XAC3787 | 1.28032 | hypothetical protein |
| XAC_RS17115 | XAC3377 | -1.25581 | DUF4381 domain-containing protein |
| dksA | - | 1.57967 | RNA polymerase-binding protein DksA |
| XAC_RS11695 | XAC2296 | 1.37448 | DUF1049 domain-containing protein |
| XAC_RS08625 | XAC1692 | -1.6812 | O-antigen translocase |
| XAC_RS00050 | XAC0009 | 2.74536 | MotA/TolQ/ExbB proton channel family protein |
| XAC_RS11340 | XAC2231 | -1.58742 | hypothetical protein |
| XAC_RS24280 | - | -1.71612 | folate hydrolase |
| XAC_RS08485 | XAC1667 | -1.39622 | FAD-binding monooxygenase |
| galU | XAC2292 | 2.79108 | UTP--glucose-1-phosphate uridylyltransferase |
| XAC_RS03970 | XAC0769 | -1.73687 | hypothetical protein |
| XAC_RS18040 | XAC3568 | 2.55366 | DNA-binding protein |
| XAC_RS07050 | XAC1378 | -1.47014 | acyl-CoA desaturase |
| XAC_RS04035 | XAC0782 | -1.02584 | cell division protein FtsQ |
| XAC_RS08170 | XAC1606 | -2.44702 | DUF2589 domain-containing protein |
| XAC_RS21010 | - | -1.04762 | GFA family protein |
| XAC_RS23120 | - | -1.91279 | hypothetical protein |
| XAC_RS03590 | XAC0692 | -1.61922 | hypothetical protein |
| XAC_RS24250 | - | -3.42191 | hypothetical protein |
| trpD | XAC0480 | -1.19051 | anthranilate phosphoribosyltransferase |
| XAC_RS24615 | - | -2.58073 | hypothetical protein |
| XAC_RS21160 | XAC4195 | -1.26771 | NdvB protein |
| XAC_RS15790 | XAC3114 | -1.05695 | pyrroloquinoline quinone biosynthesis protein B |
| XAC_RS15195 | XAC2994 | -1.48994 | MBL fold metallo-hydrolase |
| XAC_RS02405 | XAC0461 | -1.40666 | Na+/H+ antiporter subunit C |
| XAC_RS00125 | XAC0024 | -1.57683 | peptidase M23 |
| XAC_RS05695 | XAC1113 | 2.5642 | Starvation-inducible hypothetical protein |
| XAC_RS20260 | XAC4021 | -1.47699 | DUF1800 domain-containing protein |
| XAC_RS05990 | XAC1174 | -1.33514 | histidine phosphatase family protein |
| XAC_RS21515 | XAC4268 | -2.53366 | DUF465 domain-containing protein |
| XAC_RS16460 | XAC3244 | -1.67563 | dephospho-CoA kinase |
| glf | XAC3742 | -1.73097 | UDP-galactopyranose mutase |
| cas2 | XAC3843 | -2.68508 | CRISPR-associated endonuclease Cas2 |
| XAC_RS01575 | XAC0301 | -1.5225 | allantoate amidohydrolase |
| XAC_RS07655 | - | 1.79769e+308 | hypothetical protein |
| XAC_RS18475 | XAC3654 | 2.34929 | F0F1 ATP synthase subunit C |
| XAC_RS02820 | XAC0541 | 1.53015 | molecular chaperone GroES |
| XAC_RS18935 | XAC3747 | -1.91437 | glutathione-dependent formaldehyde dehydrogenase |
| XAC_RS19230 | - | -1.58548 | hypothetical protein |
| XAC_RS11010 | XAC2167 | -1.70222 | histidine kinase |
| XAC_RS14790 | XAC2914 | 2.23583 | glycine zipper 2TM domain-containing protein |
| XAC_RS18855 | XAC3731 | -1.06884 | response regulator |
| XAC_RS21475 | XAC4256 | -1.64864 | TonB-dependent receptor |
| XAC_RS10555 | XAC2077 | 1.48065 | succinate dehydrogenase flavoprotein subunit |
| XAC_RS18520 | XAC3663 | -1.85243 | DNA-deoxyinosine glycosylase |
| XAC_RS14345 | XAC2825 | -1.31516 | chloride channel protein |
| XAC_RS19270 | XAC3817 | -1.31825 | hypothetical protein |
| queG | XAC2409 | -1.33979 | tRNA epoxyqueuosine(34) reductase QueG |
| XAC_RS11270 | XAC2218 | -2.17914 | hypothetical protein |
| XAC_RS01095 | - | -1.36972 | superoxide dismutase family protein |
| pcaG | XAC0368 | -1.28504 | protocatechuate 3,4-dioxygenase subunit alpha |
| XAC_RS02180 | XAC0416 | -1.57127 | hypothetical protein |
| XAC_RS04955 | XAC0968 | 3.48707 | 30S ribosomal protein S7 |
| XAC_RS00540 | XAC0106 | -1.2728 | NUDIX domain-containing protein |
| XAC_RS12730 | XAC2504 | -1.39027 | carbohydrate porin |
| XAC_RS13480 | XAC2651 | -1.76631 | hypothetical protein |
| XAC_RS23130 | - | -1.67028 | hypothetical protein |
| XAC_RS11405 | - | -1.58167 | hypothetical protein |
| XAC_RS01350 | XAC0259 | -1.50999 | putative peptide modification system cyclase |
| XAC_RS23765 | - | -1.61488 | hypothetical protein |
| XAC_RS22605 | XAC1495 | 2.42841 | virulence regulator |
| XAC_RS24310 | - | -2.38194 | hypothetical protein |
| XAC_RS12685 | XAC2494 | -1.53465 | MFS transporter |
| XAC_RS18045 | XAC3569 | -1.52146 | membrane protein |
| XAC_RS02375 | - | -1.5872 | hypothetical protein |
| XAC_RS21460 | XAC4253 | -1.80982 | hypothetical protein |
| gspG | XAC3542 | -1.73584 | type II secretion system protein GspG |
| hisD | XAC1829 | -1.16682 | histidinol dehydrogenase |
| XAC_RS05945 | XAC1165 | -1.69089 | hypothetical protein |
| clpP | XAC1078 | 2.65323 | ATP-dependent Clp protease proteolytic subunit |
| XAC_RS11810 | XAC2319 | 2.75183 | hypothetical protein |
| recA | XAC1740 | 1.41028 | DNA recombination/repair protein RecA |
| XAC_RS08290 | XAC1628 | -1.35447 | EF-P lysine aminoacylase GenX |
| glpK | XAC0358 | 1.8117 | glycerol kinase |
| XAC_RS16945 | XAC3343 | 1.85946 | O-acetyl-ADP-ribose deacetylase |
| XAC_RS20045 | XAC3977 | -1.74276 | DUF3313 domain-containing protein |
| XAC_RS02715 | XAC0521 | -1.57418 | phosphatidate cytidylyltransferase |
| XAC_RS00105 | XAC0020 | -1.45096 | rhomboid family intramembrane serine protease |
| XAC_RS16105 | XAC3177 | -1.66857 | siderophore biosynthesis protein PvsA |
| XAC_RS00930 | XAC0179 | -1.34772 | ABC transporter ATP-binding protein |
| XAC_RS00305 | XAC0059 | -1.55273 | asparagine synthetase B |
| XAC_RS21330 | XAC4228 | -1.53127 | 9-O-acetylesterase |
| metB | XAC3039 | -1.59099 | O-succinylhomoserine (thiol)-lyase |
| XAC_RS18695 | - | 1.14945 | hypothetical protein |
| XAC_RS17050 | XAC3365 | -1.77356 | hypothetical protein |
| XAC_RS00290 | XAC0057 | -1.67212 | ABC transporter ATP-binding protein |
| XAC_RS20890 | XAC4148 | -1.64141 | AraC family transcriptional regulator |
| XAC_RS21690 | XAC4301 | -1.37971 | DUF3375 domain-containing protein |
| XAC_RS07120 | XAC1392 | -1.26392 | homocysteine S-methyltransferase |
| XAC_RS22840 | - | -2.22088 | HpaI protein |
| XAC_RS24535 | XAC4138 | -1.66148 | hypothetical protein |
| XAC_RS09735 | XAC1910 | -1.00065 | TonB-dependent receptor |
| XAC_RS01395 | XAC0268 | -1.3581 | RES domain-containing protein |
| XAC_RS01890 | XAC0357 | -1.37872 | hypothetical protein |
| XAC_RS09755 | XAC1914 | -1.43681 | type I toxin-antitoxin system SymE family toxin |
| XAC_RS11040 | XAC2172 | -1.45084 | oxidoreductase |
| XAC_RS03510 | XAC0676 | -1.45154 | SAM-dependent methyltransferase |
| XAC_RS22035 | XAC4370 | -1.4659 | tRNA uridine-5-carboxymethylaminomethyl(34) synthesis GTPase MnmE |
| XAC_RS07015 | XAC1371 | 1.66316 | RidA family protein |
| XAC_RS07460 | XAC1459 | -1.58175 | ABC transporter ATP-binding protein |
| XAC_RS05010 | XAC0979 | 3.64906 | 50S ribosomal protein L16 |
| XAC_RS07840 | XAC1540 | -1.093 | CAP domain-containing protein |
| XAC_RS22625 | - | -1.57525 | response regulator |
| pyrG | XAC1716 | 1.43823 | CTP synthetase |
| moaA | XAC1097 | -1.4672 | GTP 3',8-cyclase MoaA |
| XAC_RS20420 | XAC4054 | -1.28757 | ABC transporter ATP-binding protein |
| XAC_RS12425 | XAC2443 | 1.04884 | hypothetical protein |
| XAC_RS22300 | XACb0013 | -1.12251 | IS3 family transposase |
| XAC_RS00385 | XAC0074 | -1.72815 | TonB-dependent receptor |
| XAC_RS14780 | XAC2912 | -1.6367 | PhzF family phenazine biosynthesis protein |
| XAC_RS15875 | XAC3132 | 3.62899 | methyl-accepting chemotaxis protein |
| XAC_RS02640 | XAC0507 | -1.36749 | MFS transporter |
| XAC_RS24695 | - | -2.09833 | transposase |
| XAC_RS02100 | XAC0400 | -1.7505 | hypothetical protein |
| XAC_RS12885 | XAC2530 | -1.38507 | glycoside hydrolase family 127 protein |
| XAC_RS03540 | XAC0682 | 3.20734 | BON domain-containing protein |
| XAC_RS21755 | XAC4314 | -1.90352 | Arc family DNA-binding protein |
| XAC_RS11570 | - | -1.42676 | TIGR03751 family conjugal transfer lipoprotein |
| XAC_RS05940 | XAC1164 | -2.0844 | hypothetical protein |
| XAC_RS09060 | XAC1778 | -1.04075 | sensor kinase |
| XAC_RS11845 | XAC2326 | 1.79769e+308 | heme exporter protein CcmD |
| XAC_RS10955 | XAC2156 | 2.38189 | stress-induced protein |
| XAC_RS02450 | XAC0470 | 2.0739 | phosphoribosylaminoimidazolesuccinocarboxamide synthase |
| XAC_RS04790 | XAC0935 | -1.34388 | DUF2069 domain-containing protein |
| XAC_RS01290 | XAC0248 | -1.16916 | isoaspartyl peptidase/L-asparaginase |
| XAC_RS19040 | XAC3768 | -1.72051 | methyl-accepting chemotaxis protein |
| XAC_RS20775 | XAC4123 | -1.33274 | hypothetical protein |
| XAC_RS10105 | XAC1987 | 1.64026 | chemotaxis protein CheV |
| XAC_RS21065 | XAC4181 | -1.93163 | DUF4424 domain-containing protein |
| XAC_RS22510 | XACb0058 | -1.84684 | hypothetical protein |
| XAC_RS20400 | XAC4050 | -1.08463 | cupin-like domain-containing protein |
| XAC_RS02915 | XAC0559 | -1.08869 | PadR family transcriptional regulator |
| XAC_RS12075 | XAC2374 | -1.60458 | polygalacturonase |
| XAC_RS19585 | XAC3881 | -1.5832 | hypothetical protein |
| XAC_RS24785 | XACb0063 | -1.5294 | hypothetical protein |
| XAC_RS10375 | XAC2040 | 1.57441 | mechanosensitive ion channel family protein |
| XAC_RS06280 | XAC1230 | 3.11205 | polyketide cyclase |
| XAC_RS19520 | XAC3867 | -1.71946 | membrane protein |
| XAC_RS15410 | XAC3035 | -2.77415 | glutaredoxin family protein |
| XAC_RS18405 | XAC3640 | -1.75633 | ABC transporter ATP-binding protein |
| XAC_RS11475 | XAC2257 | -1.48475 | integrating conjugative element protein |
| XAC_RS11565 | XAC2273 | -1.70819 | TIGR03752 family integrating conjugative element protein |
| XAC_RS13430 | XAC2641 | -1.6487 | phage portal protein |
| XAC_RS03045 | - | -1.18996 | VOC family protein |
| XAC_RS17895 | XAC3540 | -1.40413 | prepilin-type N-terminal cleavage/methylation domain-containing protein |
| XAC_RS13920 | XAC2741 | -1.38113 | iron-sulfur cluster carrier protein ApbC |
| XAC_RS10905 | XAC2145 | -1.69079 | efflux RND transporter periplasmic adaptor subunit |
| XAC_RS06305 | XAC1235 | -1.76503 | hypothetical protein |
| XAC_RS01645 | XAC0314 | -1.57794 | hypothetical protein |
| XAC_RS04645 | XAC0905 | -1.15909 | LysR family transcriptional regulator |
| XAC_RS01380 | XAC0265 | 1.05849 | isovaleryl-CoA dehydrogenase |
| XAC_RS09880 | XAC1940 | -1.41986 | GGDEF domain-containing protein |
| XAC_RS04975 | XAC0972 | 2.76601 | 50S ribosomal protein L3 |
| XAC_RS14370 | XAC2830 | -1.6265 | TonB-dependent receptor |
| fabG | XAC1127 | 1.03618 | 3-oxoacyl-ACP reductase FabG |
| XAC_RS01815 | - | -1.84974 | phage-related integrase |
| XAC_RS03385 | XAC0652 | -1.64278 | S-(hydroxymethyl)glutathione dehydrogenase/class III alcohol dehydrogenase |
| pgaC | XAC1811 | -1.66467 | poly-beta-1,6 N-acetyl-D-glucosamine synthase |
| XAC_RS08685 | XAC1704 | -1.45771 | ABC transporter ATP-binding protein |
| XAC_RS00295 | XAC0058 | -1.48418 | hypothetical protein |
| XAC_RS18460 | XAC3651 | 1.63249 | ATP synthase subunit alpha |
| XAC_RS08530 | XAC1674 | -1.17157 | cytochrome c-type biogenesis protein CcmH |
| XAC_RS07200 | XAC1408 | -1.14153 | lipid-A-disaccharide synthase |
| gspL | XAC0703 | -1.58242 | type II secretion system protein GspL |
| XAC_RS04135 | XAC0802 | -1.49931 | sulfotransferase |
| XAC_RS04210 | XAC0817 | -1.82065 | sugar-binding protein |
| XAC_RS00950 | XAC0183 | -1.58749 | ABC transporter amino acid permease |
| XAC_RS07360 | XAC1439 | -1.31582 | thiopurine S-methyltransferase |
| XAC_RS22185 | XACa0029 | -1.51434 | recombinase family protein |
| XAC_RS01150 | XAC0220 | -1.23929 | rhodanese-like domain-containing protein |
| XAC_RS03670 | XAC0709 | -1.60992 | alpha-N-acetylglucosaminidase |
| XAC_RS15745 | XAC3104 | -1.60721 | energy transducer TonB |
| XAC_RS21760 | XAC4315 | -1.08405 | PIN domain-containing protein |
| XAC_RS20470 | XAC4064 | -1.70669 | transcriptional regulator FtrA |
| XAC_RS17530 | XAC3463 | 1.17361 | membrane protein |
| XAC_RS18985 | XAC3757 | -1.62724 | DUF3526 domain-containing protein |
| XAC_RS06010 | XAC1178 | -1.01957 | NAD(P)-dependent oxidoreductase |
| XAC_RS19345 | XAC3832 | -1.65482 | bifunctional isocitrate dehydrogenase kinase/phosphatase |
| XAC_RS12820 | XAC2519 | -1.58833 | phytase |
| XAC_RS02650 | XAC0509 | -1.38004 | MFS transporter |
| XAC_RS15635 | XAC3081 | -1.84599 | 6-phospho-beta-glucosidase |
| XAC_RS08635 | XAC1694 | -1.32246 | hypothetical protein |
| XAC_RS03895 | XAC0753 | -1.55764 | hypothetical protein |
| XAC_RS00705 | XAC0135 | -1.28826 | sensor histidine kinase |
| XAC_RS18795 | XAC3717 | -1.17424 | APC family permease |
| XAC_RS23700 | - | -2.19217 | hypothetical protein |
| pyk | XAC3345 | 1.19423 | pyruvate kinase |
| XAC_RS03825 | XAC0739 | -1.57823 | EamA/RhaT family transporter |
| XAC_RS24195 | - | -1.79769e+308 | magnesium transporter |
| XAC_RS18960 | XAC3752 | -1.27215 | DUF3348 domain-containing protein |
| XAC_RS00840 | XAC0160 | -1.34623 | alpha/beta hydrolase |
| XAC_RS12815 | XAC2518 | -1.71948 | phosphoesterase |
| XAC_RS17200 | XAC3395 | -1.2615 | guanylate kinase |
| XAC_RS09120 | XAC1792 | -1.71864 | DUF839 domain-containing protein |
| mtnC | XAC1838 | 1.52605 | acireductone synthase |
| XAC_RS15700 | XAC3095 | -1.59855 | GNAT family N-acetyltransferase |
| XAC_RS20825 | XAC4133 | -1.80805 | hypothetical protein |
| XAC_RS01870 | XAC0353 | -1.49506 | 3-alpha-hydroxysteroid dehydrogenase |
| XAC_RS02580 | XAC0496 | -1.45301 | DUF4126 domain-containing protein |
| XAC_RS00175 | XAC0033 | -1.53284 | glutamate synthase large subunit |
| XAC_RS21920 | XAC4347 | -1.34175 | hypothetical protein |
| XAC_RS20075 | XAC3985 | -1.24067 | AI-2E family transporter |
| XAC_RS04775 | XAC0931 | -1.59946 | Cys-tRNA(Pro) deacylase |
| XAC_RS20745 | XAC4117 | -1.91849 | serine/threonine-protein phosphatase |
| XAC_RS21340 | XAC4230 | -1.64478 | glycoside hydrolase family 43 protein |
| XAC_RS03095 | XAC0595 | -1.45188 | hypothetical protein |
| XAC_RS07615 | XAC1488 | -1.6176 | GGDEF domain-containing protein |
| XAC_RS20015 | XAC3971 | 1.25541 | hypothetical protein |
| XAC_RS19505 | XAC3864 | -1.40202 | amino acid permease |
| XAC_RS21580 | XAC4280 | -1.41425 | N-acetyltransferase |
| XAC_RS11840 | XAC2325 | -1.297 | heme ABC transporter permease |
| XAC_RS22790 | - | -2.12778 | hypothetical protein |
| XAC_RS02710 | XAC0520 | -1.61157 | 1-acyl-sn-glycerol-3-phosphate acyltransferase |
| XAC_RS21640 | XAC4292 | -1.42949 | membrane protein |
| XAC_RS03110 | XAC0597 | -1.29084 | EamA/RhaT family transporter |
| XAC_RS04060 | XAC0787 | -1.19591 | M23 family peptidase |
| XAC_RS06140 | XAC1203 | -1.83104 | transporter |
| XAC_RS12835 | XAC2522 | -1.01744 | cellulase |
| XAC_RS15475 | XAC3048 | -1.05941 | heat-shock protein Hsp33 |
| minD | XAC1225 | 1.00244 | septum site-determining protein MinD |
| XAC_RS24165 | - | -1.88118 | hypothetical protein |
| XAC_RS17055 | XAC3366 | -1.76741 | TonB-dependent receptor |
| XAC_RS15970 | XAC3150 | -1.38051 | crossover junction endodeoxyribonuclease RuvC |
| XAC_RS21525 | XAC4270 | -1.04206 | glycerol-3-phosphate 1-O-acyltransferase PlsB |
| XAC_RS05680 | XAC1110 | 2.08856 | YbaB/EbfC family nucleoid-associated protein |
| XAC_RS09635 | - | -2.58232 | hypothetical protein |
| XAC_RS03130 | XAC0601 | -2.24793 | hypothetical protein |
| XAC_RS15265 | XAC3007 | 1.24096 | PspA/IM30 family protein |
| XAC_RS06005 | XAC1177 | -1.70019 | glycoside hydrolase family 15 protein |
| XAC_RS07180 | XAC1404 | -1.2355 | hypothetical protein |
| XAC_RS14330 | XAC2822 | -1.39354 | DNA-3-methyladenine glycosylase 2 family protein |
| XAC_RS16975 | XAC3350 | -1.28026 | DUF2339 domain-containing protein |
| XAC_RS07780 | XAC1528 | -1.18604 | ABC transporter ATP-binding protein |
| XAC_RS16360 | XAC3226 | -1.48128 | DUF4158 domain-containing protein |
| XAC_RS11390 | XAC2241 | -1.36957 | hypothetical protein |
| XAC_RS02115 | XAC0403 | -1.71806 | YscQ/HrcQ family type III secretion apparatus protein |
| XAC_RS11370 | XAC2237 | -1.01847 | DUF3275 domain-containing protein |
| XAC_RS03365 | XAC0648 | -1.71231 | PQQ-dependent dehydrogenase, methanol/ethanol family |
| XAC_RS17960 | XAC3552 | -1.57249 | RDD family protein |
| treZ | XAC0427 | -1.03025 | malto-oligosyltrehalose trehalohydrolase |
| XAC_RS03780 | XAC0731 | -1.40468 | glycosyl transferase |
| XAC_RS20585 | XAC4086 | -1.03811 | beta-ketoacyl-[acyl-carrier-protein |
| XAC_RS08575 | - | -1.53045 | hypothetical protein |
| XAC_RS17445 | XAC3446 | -1.56778 | biopolymer transporter Tol |
| XAC_RS11610 | XAC2281 | -1.37382 | DNA repair protein RadC |
| XAC_RS17170 | XAC3389 | 5.25644 | 50S ribosomal protein L31 type B |
| XAC_RS16815 | - | -1.95572 | hypothetical protein |
| XAC_RS22160 | XACa0024 | -2.65169 | hypothetical protein |
| XAC_RS06990 | XAC1366 | -1.24045 | hypothetical protein |
| XAC_RS17470 | XAC3451 | -1.2177 | ketol-acid reductoisomerase |
| XAC_RS23250 | - | -1.55466 | asparagine synthase |
| XAC_RS17370 | XAC3431 | -1.29403 | short chain dehydrogenase |
| XAC_RS16550 | XAC3267 | -1.29502 | DUF1629 domain-containing protein |
| XAC_RS20865 | XAC4143 | -1.67318 | type VI secretion system baseplate subunit TssE |
| XAC_RS13045 | XAC2561 | -1.13829 | membrane protein |
| XAC_RS15285 | XAC3011 | -1.32021 | 3-dehydroquinate synthase |
| XAC_RS11520 | - | -1.86067 | DUF4124 domain-containing protein |
| XAC_RS07415 | XAC1450 | -1.29943 | MFS transporter |
| XAC_RS20350 | XAC4040 | 1.13472 | porphobilinogen synthase |
| XAC_RS00750 | XAC0144 | -1.19408 | TonB-dependent receptor |
| XAC_RS02025 | XAC0384 | -1.3908 | 3-oxoacyl-ACP reductase |
| flgM | XAC1989 | 3.36571 | flagellar biosynthesis anti-sigma factor FlgM |
| XAC_RS15425 | XAC3038 | -1.88452 | homoserine dehydrogenase |
| XAC_RS01185 | XAC0227 | -1.80139 | hypothetical protein |
| gspD | XAC3534 | -1.65942 | type II secretion system protein GspD |
| XAC_RS02780 | XAC0534 | -1.28914 | cytochrome C biogenesis protein |
| XAC_RS14485 | XAC2853 | -1.8543 | peptidase C1 |
| XAC_RS18680 | XAC3692 | -1.47669 | hypothetical protein |
| XAC_RS10150 | XAC1996 | 3.6131 | chemotaxis protein |
| XAC_RS16515 | XAC3257 | 1.2018 | hypothetical protein |
| XAC_RS19405 | XAC3844 | -1.40977 | hypothetical protein |
| XAC_RS06835 | XAC1338 | -1.58898 | FAD-dependent monooxygenase |
| XAC_RS03725 | XAC0720 | -1.35875 | high-affinity choline transporter BetT |
| XAC_RS14310 | XAC2818 | -1.10024 | membrane protein |
| XAC_RS04055 | - | -1.24349 | DUF721 domain-containing protein |
| XAC_RS05040 | XAC0985 | 3.13197 | 30S ribosomal protein S14 |
| XAC_RS24020 | XAC3159 | -1.68531 | phospholipase C |
| XAC_RS16490 | XAC3250 | 1.37151 | DNA-binding response regulator |
| hflB | XAC1732 | 1.28614 | ATP-dependent metallopeptidase FtsH/Yme1/Tma family protein |
| XAC_RS05055 | XAC0988 | 1.69654 | 50S ribosomal protein L18 |
| XAC_RS18455 | XAC3650 | 1.15835 | ATP synthase subunit gamma |
| XAC_RS17230 | XAC3401 | -1.50222 | DUF1631 domain-containing protein |
| XAC_RS11375 | XAC2238 | -1.82139 | hypothetical protein |
| XAC_RS21790 | XAC4321 | -1.81798 | hypothetical protein |
| XAC_RS05885 | XAC1154 | 1.45817 | response regulator |
| XAC_RS18605 | XAC3677 | -1.65199 | HAD family phosphatase |
| XAC_RS10030 | XAC1972 | 2.58876 | hypothetical protein |
| XAC_RS21285 | XAC4220 | -1.06758 | ferrochelatase |
| XAC_RS03815 | XAC0737 | -1.5403 | PLP-dependent aminotransferase family protein |
| XAC_RS19955 | XAC3959 | -1.13401 | acyl-CoA desaturase |
| XAC_RS15570 | XAC3068 | -1.18232 | adenosylmethionine--8-amino-7-oxononanoate transaminase |
| XAC_RS00650 | XAC0126 | -1.22009 | TonB-dependent receptor |
| atpD | XAC3649 | 1.65791 | ATP synthase subunit beta |
| XAC_RS22845 | - | -2.0811 | hypothetical protein |
| XAC_RS04710 | XAC0918 | -1.16758 | NAD(P) transhydrogenase subunit alpha |
| XAC_RS13435 | XAC2642 | -1.74645 | terminase |
| XAC_RS02065 | XAC0393 | -1.81116 | type III secretion system effector XopAE |
| XAC_RS04510 | XAC0880 | -1.73001 | transcriptional regulator |
| XAC_RS13465 | XAC2648 | -1.87009 | membrane protein |
| XAC_RS18670 | XAC3690 | -1.36839 | hypothetical protein |
| folK | XAC1784 | -1.29664 | 2-amino-4-hydroxy-6-hydroxymethyldihydropteridine diphosphokinase |
| XAC_RS11940 | XAC2345 | -1.15849 | argininosuccinate lyase |
| XAC_RS15675 | XAC3090 | -1.8661 | hypothetical protein |
| XAC_RS22110 | - | -1.31019 | plasmid stabilization protein |
| XAC_RS01985 | XAC0376 | -1.17192 | DUF2490 domain-containing protein |
| gspH | XAC3541 | -1.56992 | type II secretion system protein GspH |
| XAC_RS11365 | XAC2236 | -1.74284 | DUF3577 domain-containing protein |
| XAC_RS24660 | XAC4356 | -1.97915 | hypothetical protein |
| XAC_RS11600 | XAC2279 | -1.2266 | amino acid synthesis family protein |
| XAC_RS08670 | XAC1701 | -1.81548 | hypothetical protein |
| XAC_RS04785 | - | -1.92483 | hypothetical protein |
| ligK | XAC4155 | -1.61984 | 4-carboxy-4-hydroxy-2-oxoadipate aldolase/oxaloacetate decarboxylase |
| XAC_RS20475 | XAC4066 | -1.10461 | ferredoxin--NADP reductase |
| rsmD | XAC2527 | -1.44355 | 16S rRNA (guanine(966)-N(2))-methyltransferase RsmD |
| XAC_RS14205 | XAC2798 | -1.74813 | plasmid pRiA4b ORF-3 family protein |
| XAC_RS23975 | - | -1.79769e+308 | hypothetical protein |
| XAC_RS02625 | XAC0505 | -1.62098 | DUF2752 domain-containing protein |
| XAC_RS07115 | XAC1391 | -1.50143 | S-methylmethionine permease |
| XAC_RS02315 | XAC0442 | -1.17458 | ATP-dependent helicase |
| XAC_RS22560 | XACb0071 | -1.616 | recombinase family protein |
| XAC_RS05295 | XAC1035 | -1.40782 | hypothetical protein |
| XAC_RS02155 | XAC0411 | -1.88608 | HrpE/YscL family type III secretion apparatus protein |
| rarD | - | -1.18964 | protein RarD |
| XAC_RS11035 | XAC2171 | -1.1566 | LysR family transcriptional regulator |
| XAC_RS07720 | XAC1516 | 1.85625 | outer membrane protein assembly factor BamE |
| XAC_RS05280 | XAC1033 | -1.61697 | DUF455 domain-containing protein |
| XAC_RS22950 | XAC0617 | -1.92883 | hypothetical protein |
| XAC_RS05655 | XAC1105 | -1.29761 | hypothetical protein |
| XAC_RS05855 | XAC1147 | -1.4514 | glycerophosphodiester phosphodiesterase |
| XAC_RS18150 | XAC3590 | -1.42296 | FAD-binding oxidoreductase |
| XAC_RS08630 | XAC1693 | -1.66924 | glycosyl transferase |
| XAC_RS17780 | XAC3517 | -1.6299 | divalent ion tolerance protein CutA |
| XAC_RS16165 | XAC3189 | -1.91566 | threonine-phosphate decarboxylase |
| XAC_RS20265 | XAC4022 | -1.44635 | two-component sensor histidine kinase |
| XAC_RS24210 | - | -1.66772 | IS3 family transposase |
| pal | XAC3141 | 2.91057 | peptidoglycan-associated lipoprotein Pal |
| XAC_RS22015 | XAC4366 | -1.32414 | rhomboid family intramembrane serine protease |
| XAC_RS02160 | XAC0412 | -1.83484 | EscN/YscN/HrcN family type III secretion system ATPase |
| XAC_RS20570 | XAC4083 | -1.89078 | hypothetical protein |
| XAC_RS09035 | XAC1773 | -1.24752 | DUF5110 domain-containing protein |
| XAC_RS07045 | XAC1377 | -1.08758 | FAD-dependent oxidoreductase |
| XAC_RS08185 | - | -1.95248 | hypothetical protein |
| XAC_RS06200 | XAC1215 | -1.34544 | MFS transporter |
| XAC_RS17875 | XAC3536 | -1.30397 | general secretion pathway protein GspM |
| XAC_RS09250 | XAC1819 | -1.67708 | tryptophan-rich sensory protein |
| XAC_RS13295 | XAC2613 | 1.23462 | hypothetical protein |
| XAC_RS17570 | XAC3471 | 1.38234 | C4-dicarboxylate transporter |
| XAC_RS08355 | XAC1641 | -1.27611 | hypothetical protein |
| XAC_RS12550 | XAC2468 | -1.00589 | magnesium and cobalt transport protein CorA |
| XAC_RS09240 | XAC1816 | -1.86222 | hemagglutinin |
| XAC_RS03655 | XAC0705 | -1.86802 | type II secretion system protein N |
| XAC_RS07340 | XAC1435 | -1.27262 | TonB-dependent siderophore receptor |
| XAC_RS14215 | XAC2800 | -1.75923 | efflux RND transporter periplasmic adaptor subunit |
| XAC_RS17425 | XAC3442 | 3.28611 | inorganic pyrophosphatase |
| XAC_RS14400 | XAC2837 | -1.45349 | MFS transporter |
| XAC_RS15365 | XAC3026 | -1.75645 | AraC family transcriptional regulator |
| XAC_RS07070 | XAC1382 | -1.93462 | hypothetical protein |
| XAC_RS09820 | XAC1929 | -1.89858 | transposase |
| XAC_RS23380 | - | -2.05238 | hypothetical protein |
| XAC_RS01630 | XAC0311 | -1.65054 | aromatic ring-hydroxylating dioxygenase subunit alpha |
| XAC_RS14395 | XAC2835 | -1.60345 | aldo/keto reductase |
| XAC_RS15820 | XAC3120 | -1.12106 | glucokinase |
| XAC_RS00735 | XAC0141 | 1.33486 | enoyl-[acyl-carrier-protein |
| XAC_RS09610 | XAC1889 | 1.82402 | chemoreceptor glutamine deamidase CheD |
| XAC_RS05220 | XAC1021 | 1.83611 | hypothetical protein |
| XAC_RS16485 | XAC3249 | -1.47372 | sensor histidine kinase |
| XAC_RS01995 | XAC0378 | 1.06546 | hypothetical protein |
| XAC_RS23085 | - | -1.79769e+308 | hypothetical protein |
| XAC_RS02345 | XAC0448 | -1.19086 | tryptophan 2,3-dioxygenase |
| XAC_RS09705 | XAC1905 | 3.47079 | STAS domain-containing protein |
| XAC_RS00375 | XAC0072 | -1.25758 | saccharopine dehydrogenase |
| XAC_RS18410 | XAC3641 | -1.78402 | ABC transporter permease |
| XAC_RS20035 | XAC3975 | -1.40121 | two-component sensor histidine kinase |
| XAC_RS10795 | XAC2125 | -1.58874 | glycosyl transferase |
| XAC_RS00100 | XAC0019 | 2.1504 | membrane protein |
| XAC_RS20515 | XAC4072 | -1.30955 | thioredoxin |
| XAC_RS23245 | XAC1490 | -1.86711 | hypothetical protein |
| XAC_RS02745 | XAC0526 | -1.07054 | 50S ribosomal protein L11 methyltransferase |
| bioH | XAC0385 | -1.12376 | pimeloyl-[acyl-carrier protein |
| XAC_RS08040 | XAC1581 | -1.45816 | SulP family inorganic anion transporter |
| XAC_RS15615 | XAC3077 | -1.90808 | TonB-dependent receptor |
| XAC_RS06970 | XAC1363 | -1.60557 | MFS transporter |
| XAC_RS14380 | XAC2832 | -1.68033 | hypothetical protein |
| XAC_RS10740 | XAC2114 | -1.60657 | zinc transporter ZupT |
| XAC_RS20105 | XAC3991 | -1.08485 | cytochrome b |
| XAC_RS07035 | XAC1375 | -1.16214 | class I SAM-dependent methyltransferase |
| XAC_RS00890 | XAC0171 | -1.76876 | cupin domain-containing protein |
| XAC_RS02955 | XAC0567 | -1.32651 | hypothetical protein |
| XAC_RS06600 | XAC1292 | 1.49219 | 30S ribosomal protein S16 |
| XAC_RS16215 | XAC3199 | -1.27343 | glyceraldehyde 3-phosphate reductase |
| XAC_RS00170 | XAC0032 | -1.4988 | glutamate synthase subunit beta |
| XAC_RS09775 | XAC1918 | -1.16033 | hemolysin |
| XAC_RS13935 | XAC2744 | -1.29897 | NAD(P)/FAD-dependent oxidoreductase |
| XAC_RS20660 | XAC4100 | -1.43891 | ketosynthase |
| XAC_RS16735 | XAC3304 | -1.42777 | SLC13 family permease |
| XAC_RS06230 | XAC1221 | -1.19025 | DNA-binding response regulator |
| XAC_RS09810 | XAC1927 | -1.52703 | radical SAM protein |
| XAC_RS06885 | XAC1347 | 5.13938 | hypothetical protein |
| XAC_RS14700 | XAC2896 | -1.33752 | NAD(P)-dependent alcohol dehydrogenase |
| XAC_RS19490 | XAC3861 | -1.79414 | hypothetical protein |
| acnA | XAC1882 | 1.97738 | aconitate hydratase AcnA |
| XAC_RS08665 | XAC1700 | -1.5841 | glycosyltransferase family 1 protein |
| XAC_RS06695 | XAC1310 | -1.55596 | TonB-dependent receptor |
| XAC_RS06660 | XAC1304 | -1.8843 | type IV secretion protein Rhs |
| XAC_RS15620 | XAC3078 | -1.78927 | beta-galactosidase |
| XAC_RS13800 | XAC2718 | -1.5122 | LysR family transcriptional regulator |
| XAC_RS21245 | XAC4212 | -1.16661 | type II/IV secretion system protein |
| XAC_RS04105 | XAC0797 | -1.30231 | gfo/Idh/MocA family oxidoreductase |
| XAC_RS20295 | XAC4028 | -1.48385 | ankyrin repeat domain-containing protein |
| XAC_RS08435 | - | -3.9266 | AlpA family phage regulatory protein |
| XAC_RS09115 | - | -1.58858 | hypothetical protein |
| XAC_RS03955 | XAC0764 | -1.96597 | YraN family protein |
| XAC_RS10045 | XAC1975 | 4.40245 | flagellin |
| cysT | XAC1018 | -1.41338 | sulfate ABC transporter permease subunit CysT |
| XAC_RS06035 | XAC1182 | -1.20442 | FAD-dependent oxidoreductase |
| XAC_RS14025 | XAC2762 | -1.13358 | polyprenyl synthetase family protein |
| XAC_RS15735 | XAC3102 | 2.96786 | response regulator |
| XAC_RS07500 | XAC1468 | -1.08434 | hypothetical protein |
| XAC_RS20750 | XAC4118 | -1.58633 | type VI secretion system-associated protein TagF |
| XAC_RS17620 | XAC3481 | -1.3394 | ABC transporter substrate-binding protein |
| XAC_RS11070 | XAC2179 | -1.18669 | DNA repair protein RadC |
| XAC_RS15375 | XAC3028 | -1.68266 | histidine kinase |
| XAC_RS10920 | XAC2149 | -1.60903 | sensor histidine kinase |
| XAC_RS04230 | XAC0822 | 1.97542 | hemin uptake protein HemP |
| phoB | XAC1042 | -1.22768 | phosphate regulon transcriptional regulatory protein PhoB |
| XAC_RS05600 | XAC1096 | -1.25951 | MBL fold metallo-hydrolase |
| pgsA | XAC2093 | -1.76262 | CDP-diacylglycerol--glycerol-3-phosphate 3-phosphatidyltransferase |
| cysI | XAC3331 | -1.59654 | assimilatory sulfite reductase (NADPH) hemoprotein subunit |
| XAC_RS11625 | XAC2284 | -1.82231 | integrating conjugative element protein |
| XAC_RS20840 | - | -3.11549 | hypothetical protein |
| xth | XAC4171 | -1.49 | exodeoxyribonuclease III |
| XAC_RS01155 | XAC0221 | 2.66976 | protein-export protein SecB |
| XAC_RS00485 | XAC0095 | -3.75149 | hypothetical protein |
| XAC_RS22610 | XAC1496 | -1.16398 | hypothetical protein |
| XAC_RS24525 | - | -2.37873 | hypothetical protein |
| XAC_RS19290 | XAC3821 | -1.69168 | hypothetical protein |
| XAC_RS20000 | XAC3969 | -1.55592 | DUF3182 domain-containing protein |
| XAC_RS01920 | XAC0363 | -1.65834 | aromatic ring-hydroxylating dioxygenase subunit alpha |
| XAC_RS11325 | XAC2228 | -1.79001 | TetR/AcrR family transcriptional regulator |
| XAC_RS20425 | XAC4055 | -1.40284 | cysteine proteinase |
| XAC_RS17830 | XAC3526 | -1.48298 | glycosyltransferase family 2 protein |
| XAC_RS15170 | XAC2988 | -1.13693 | LuxR family transcriptional regulator |
| XAC_RS20635 | XAC4095 | -2.21188 | hypothetical protein |
| XAC_RS16160 | XAC3188 | -1.62812 | cobyric acid synthase CobQ |
| XAC_RS19760 | XAC3917 | -1.02981 | SPOR domain-containing protein |
| XAC_RS03105 | XAC0596 | -1.07991 | SAM-dependent methyltransferase |
| XAC_RS09020 | XAC1770 | -1.6783 | hypothetical protein |
| XAC_RS09100 | XAC1787 | 2.21198 | aspartate 1-decarboxylase |
| XAC_RS03190 | XAC0612 | 1.1083 | endoglucanase |
| XAC_RS09070 | XAC1781 | -1.69477 | GTP cyclohydrolase I FolE2 |
| XAC_RS22020 | XAC4367 | -1.75723 | glycerophosphoryl diester phosphodiesterase |
| XAC_RS00570 | XAC0110 | -1.52282 | proline/glycine betaine transporter ProP |
| cydB | XAC3736 | -1.31555 | cytochrome d ubiquinol oxidase subunit II |
| XAC_RS08135 | XAC1599 | -1.28974 | exodeoxyribonuclease I |
| XAC_RS13205 | XAC2593 | 1.01037 | translation initiation factor IF-3 |
| XAC_RS11245 | XAC2213 | -1.97572 | DNA cytosine methyltransferase |
| XAC_RS11535 | XAC2269 | -1.96341 | hypothetical protein |
| XAC_RS03450 | XAC0664 | 1.09587 | D-alanyl-D-alanine carboxypeptidase |
| XAC_RS04605 | XAC0896 | -1.42187 | response regulator |
| XAC_RS01275 | XAC0245 | -1.19866 | DUF2059 domain-containing protein |
| XAC_RS18970 | - | -1.647 | hypothetical protein |
| XAC_RS08180 | XAC1608 | -2.40555 | hypothetical protein |
| XAC_RS03810 | - | -1.77483 | hypothetical protein |
| XAC_RS22705 | XAC0040 | -2.1686 | hypothetical protein |
| XAC_RS14825 | XAC2920 | -1.36421 | DUF72 domain-containing protein |
| XAC_RS03075 | XAC0592 | -1.20011 | DUF1615 domain-containing protein |
| XAC_RS13490 | XAC2653 | -2.03899 | phage virion morphogenesis protein |
| XAC_RS05875 | XAC1151 | 3.13123 | Hsp20/alpha crystallin family protein |
| XAC_RS20815 | XAC4131 | -1.76634 | TonB-dependent receptor |
| XAC_RS15565 | XAC3067 | -1.25788 | ADP compounds hydrolase NudE |
| XAC_RS17845 | - | -1.41733 | TonB-dependent siderophore receptor |
| XAC_RS08165 | XAC1605 | -1.24874 | acetylmuramidase |
| XAC_RS22885 | - | -2.34222 | hypothetical protein |
| XAC_RS03015 | XAC0580 | -1.67028 | NAD(P)-dependent oxidoreductase |
| XAC_RS07425 | - | 2.76197 | hypothetical protein |
| XAC_RS14055 | XAC2768 | -1.36978 | hypothetical protein |
| XAC_RS01775 | XAC0339 | -1.89676 | NAD(P)-dependent oxidoreductase |
| XAC_RS13135 | XAC2579 | -1.00845 | glycosyltransferase family 1 protein |
| XAC_RS18440 | XAC3647 | -1.4407 | gamma subclass chorismate mutase AroQ |
| XAC_RS07770 | XAC1526 | -1.67564 | TolC family protein |
| recB | XAC4336 | -1.6735 | exodeoxyribonuclease V subunit beta |
| XAC_RS02930 | XAC0562 | -1.27728 | biotin-independent malonate decarboxylase subunit beta |
| XAC_RS10625 | XAC2090 | -1.05015 | low molecular weight phosphotyrosine protein phosphatase |
| XAC_RS06650 | XAC1302 | -1.49157 | DUF937 domain-containing protein |
| XAC_RS03660 | XAC0706 | -1.87216 | TonB-dependent receptor |
| XAC_RS15805 | XAC3117 | -1.51262 | coenzyme PQQ synthesis protein E |
| XAC_RS18470 | XAC3653 | 1.63268 | ATP synthase subunit B |
| XAC_RS18270 | XAC3613 | -1.84119 | TonB-dependent receptor |
| XAC_RS18420 | - | -1.13625 | sigma-54-dependent Fis family transcriptional regulator |
| XAC_RS02535 | XAC0487 | 2.56894 | 50S ribosomal protein L13 |
| XAC_RS00255 | - | -1.65445 | methyltransferase type 12 |
| XAC_RS16125 | XAC3181 | -1.57864 | siderophore biosynthesis PLP-dependent protein |
| XAC_RS13225 | XAC2597 | -1.30441 | MFS transporter |
| XAC_RS14885 | XAC2932 | -1.63398 | type 1 glutamine amidotransferase |
| XAC_RS21435 | XAC4249 | -1.46388 | endo-1,4-beta-xylanase |
| XAC_RS16110 | XAC3178 | -1.47718 | IucA/IucC family siderophore biosynthesis protein |
| XAC_RS23460 | - | -1.72972 | hypothetical protein |
| XAC_RS12905 | XAC2534 | -1.64815 | carbohydrate-binding protein |
| XAC_RS09595 | XAC1886 | -1.42037 | 2-succinyl-6-hydroxy-2,4-cyclohexadiene-1-carboxylate synthase |
| XAC_RS15230 | XAC3001 | -1.72198 | MFS transporter |
| XAC_RS05260 | XAC1029 | -1.1127 | bifunctional tetrahydrofolate synthase/dihydrofolate synthase |
| XAC_RS09270 | XAC1823 | -1.36429 | threonine synthase |
| XAC_RS08475 | XAC1665 | -1.21055 | DUF488 domain-containing protein |
| XAC_RS01060 | XAC0202 | -1.15326 | META domain-containing protein |
| XAC_RS23800 | XAC2635 | 2.45641 | DUF1629 domain-containing protein |
| ssuB | XAC3196 | -1.52583 | ABC transporter ATP-binding protein |
| XAC_RS00990 | XAC0190 | 1.01204 | hypothetical protein |
| XAC_RS14235 | XAC2804 | -1.62485 | two-component sensor histidine kinase |
| XAC_RS02995 | XAC0575 | -1.55346 | arabinogalactan endo-1,4-beta-galactosidase |
| XAC_RS03975 | XAC0770 | -1.33476 | hypothetical protein |
| XAC_RS16940 | XAC3342 | -1.21245 | isoprenylcysteine carboxylmethyltransferase family protein |
| XAC_RS07040 | XAC1376 | -1.32987 | DUF1365 domain-containing protein |
| tatC | XAC4216 | -1.20261 | twin-arginine translocase subunit TatC |
| XAC_RS20710 | XAC4110 | -1.03616 | DNA polymerase I |
| XAC_RS09455 | XAC1860 | -1.15011 | 4-hydroxy-tetrahydrodipicolinate reductase |
| XAC_RS06710 | XAC1313 | 2.01345 | acyl-CoA dehydrogenase |
| XAC_RS17180 | XAC3391 | -1.24902 | DNA helicase RecG |
| XAC_RS11795 | XAC2316 | -1.31506 | segregation/condensation protein A |
| XAC_RS23225 | XAC1361 | -1.49194 | hypothetical protein |
| XAC_RS02485 | XAC0477 | -1.48693 | amino acid lyase |
| XAC_RS13370 | XAC2628 | -1.82108 | integrase |
| XAC_RS10840 | XAC2133 | -1.33756 | oxidoreductase |
| XAC_RS01440 | - | -1.87234 | hypothetical protein |
| XAC_RS06125 | XAC1200 | -1.52677 | S9 family peptidase |
| XAC_RS23970 | XAC3088 | -1.39431 | hypothetical protein |
| XAC_RS17545 | XAC3466 | -1.38288 | O-antigen ligase family protein |
| XAC_RS05505 | XAC1077 | 1.50379 | trigger factor |
| XAC_RS13075 | XAC2567 | -1.67416 | hypothetical protein |
| XAC_RS17030 | XAC3361 | -1.34804 | hypothetical protein |
| XAC_RS03280 | XAC0632 | -1.88198 | DUF454 domain-containing protein |
| XAC_RS16410 | XAC3236 | 3.13579 | succinyl-CoA ligase subunit beta |
| atzF | XAC4327 | -1.79871 | allophanate hydrolase |
| XAC_RS00825 | XAC0157 | -1.57299 | metallophosphoesterase |
| XAC_RS11005 | XAC2166 | -1.46079 | AraC family transcriptional regulator |
| XAC_RS21900 | XAC4343 | 1.39075 | STAS domain-containing protein |
| XAC_RS08655 | XAC1698 | -1.55418 | class I SAM-dependent methyltransferase |
| XAC_RS12135 | XAC2386 | 2.56898 | superoxide dismutase |
| XAC_RS04455 | XAC0869 | -1.33391 | acetoin utilization protein |
| XAC_RS17735 | XAC3505 | -1.23768 | rhamnogalacturonase B |
| XAC_RS14955 | XAC2946 | -1.46348 | DUF4198 domain-containing protein |
| XAC_RS01725 | XAC0330 | -1.60028 | conditioned medium factor |
| XAC_RS01980 | XAC0375 | -1.27707 | alpha/beta hydrolase |
| XAC_RS22675 | - | -4.67233 | hypothetical protein |
| XAC_RS17870 | XAC3535 | -1.01079 | hypothetical protein |
| XAC_RS14915 | XAC2938 | -1.05883 | cysteine desulfurase |
| XAC_RS18875 | XAC3735 | -1.58326 | cytochrome ubiquinol oxidase subunit I |
| XAC_RS21440 | XAC4250 | -1.52679 | DUF4982 domain-containing protein |
| XAC_RS09255 | XAC1820 | -1.5307 | bifunctional aspartate kinase/homoserine dehydrogenase I |
| XAC_RS11095 | XAC2183 | -1.75694 | DUF4102 domain-containing protein |
| XAC_RS14420 | XAC2840 | -1.68233 | cupin domain-containing protein |
| XAC_RS13140 | XAC2580 | -1.58339 | GumG protein |
| XAC_RS01850 | XAC0349 | -1.46838 | MFS transporter |
| XAC_RS14410 | - | -1.79769e+308 | hypothetical protein |
| XAC_RS08195 | - | -1.6511 | hypothetical protein |
| XAC_RS09565 | XAC1881 | -1.27081 | hypothetical protein |
| rpoH | XAC3824 | 1.25014 | RNA polymerase sigma factor RpoH |
| XAC_RS08380 | XAC1646 | -1.75345 | sulfoxide reductase heme-binding subunit YedZ |
| XAC_RS22225 | XACa0039 | -1.68617 | avirulence protein |
| XAC_RS18915 | XAC3743 | -1.7829 | beta-glucosidase |
| XAC_RS18980 | XAC3756 | -1.77392 | TonB-dependent siderophore receptor |
| XAC_RS16390 | - | -1.65001 | hypothetical protein |
| XAC_RS23510 | - | -3.46916 | hypothetical protein |
| XAC_RS02140 | XAC0408 | -1.93555 | type III secretion protein HrpB2 |
| XAC_RS12655 | XAC2488 | 2.25181 | MFS transporter |
| nth | XAC1582 | -1.06627 | endonuclease III |
| XAC_RS02175 | XAC0415 | -1.69825 | EscC/YscC/HrcC family type III secretion system outer membrane ring protein |
| XAC_RS16450 | XAC3242 | 1.20786 | type II secretion system F family protein |
| XAC_RS18425 | - | -1.40926 | ATP-binding protein |
| XAC_RS11085 | XAC2181 | -1.5562 | nucleotidyl transferase AbiEii/AbiGii toxin family protein |
| XAC_RS10215 | XAC2009 | -1.7552 | hypothetical protein |
| XAC_RS00040 | XAC0007 | 1.54618 | tetratricopeptide repeat protein |
| XAC_RS15880 | XAC3133 | -1.63292 | amino acid transporter |
| XAC_RS11560 | XAC2272 | -1.85274 | TIGR03749 family integrating conjugative element protein |
| XAC_RS20555 | XAC4080 | -1.50006 | glutathione-regulated potassium-efflux system protein |
| XAC_RS22025 | XAC4368 | -1.71072 | TonB-dependent receptor |
| pdxA | XAC0864 | -1.37225 | 4-hydroxythreonine-4-phosphate dehydrogenase PdxA |
| XAC_RS06325 | XAC1239 | -1.06521 | arginyl-tRNA-protein transferase |
| XAC_RS17950 | XAC3550 | 1.22585 | hypothetical protein |
| rfbC | XAC3583 | 1.36478 | dTDP-4-dehydrorhamnose 3,5-epimerase |
| XAC_RS02840 | XAC0545 | -1.68229 | 3-deoxy-7-phosphoheptulonate synthase |
| XAC_RS04800 | XAC0937 | -1.30056 | hypothetical protein |
| XAC_RS15085 | XAC2972 | -1.13802 | RNA polymerase sigma-54 factor |
| XAC_RS09765 | XAC1916 | -1.75899 | IS4/IS5 family transposase |
| groL | XAC0542 | 3.41484 | molecular chaperone GroEL |
| XAC_RS13285 | - | 2.15906 | hypothetical protein |
| XAC_RS02905 | XAC0557 | -1.52937 | histidine-type phosphatase |
| XAC_RS20940 | XAC4157 | -1.7265 | amidohydrolase |
| XAC_RS11000 | XAC2165 | -1.85381 | hydrolase |
| XAC_RS11015 | XAC2168 | -1.15658 | DNA-binding response regulator |
| XAC_RS01450 | XAC0278 | -1.4502 | hypothetical protein |
| XAC_RS02285 | XAC0436 | -1.65519 | transporter |
| XAC_RS09800 | XAC1925 | -1.51439 | transcriptional regulator |
| XAC_RS08545 | XAC1677 | -1.55881 | cytochrome c-type biogenesis protein CcmE 1 |
| XAC_RS15750 | XAC3105 | -1.40814 | ADP-ribosylglycohydrolase family protein |
| XAC_RS03330 | XAC0642 | -1.54449 | MFS transporter |
| XAC_RS03060 | XAC0589 | -1.16831 | hypothetical protein |
| XAC_RS20415 | XAC4053 | -1.38992 | ABC transporter permease |
| XAC_RS08620 | XAC1691 | -1.65989 | aminotransferase class V-fold PLP-dependent enzyme |
| XAC_RS07645 | - | -1.36355 | hypothetical protein |
| XAC_RS19570 | XAC3878 | -1.6706 | thiol reductase thioredoxin |
| XAC_RS08465 | XAC1662 | -1.7705 | hypothetical protein |
| XAC_RS08990 | XAC1765 | -1.54028 | D-galactonate dehydratase |
| XAC_RS19690 | XAC3903 | -1.17325 | orotate phosphoribosyltransferase |
| XAC_RS11945 | XAC2346 | -1.61282 | N-acetyl-gamma-glutamyl-phosphate reductase |
| XAC_RS08250 | XAC1620 | 3.56011 | 30S ribosomal protein S6 |
| XAC_RS13695 | XAC2699 | 1.59907 | NADH-quinone oxidoreductase subunit F |
| ftsW | XAC0778 | -1.01855 | putative lipid II flippase FtsW |
| XAC_RS15760 | XAC3107 | -1.28689 | ATP-dependent DNA helicase |
| XAC_RS05710 | XAC1116 | -1.16679 | MoxR family ATPase |
| XAC_RS11290 | XAC2222 | -1.93436 | DUF4102 domain-containing protein |
| XAC_RS20450 | XAC4060 | -1.6991 | cation transporter |
| XAC_RS19480 | XAC3859 | -1.27946 | D-alanyl-D-alanine dipeptidase |
| trpC | XAC0481 | -1.20954 | indole-3-glycerol-phosphate synthase |
| flhA | XAC1936 | -1.49942 | flagellar biosynthesis protein FlhA |
| XAC_RS04100 | - | -2.36417 | lactoylglutathione lyase |
| hisA | XAC1833 | -1.11108 | 1-(5-phosphoribosyl)-5-((5-phosphoribosylamino)methylideneamino)imidazole-4-carboxamide isomerase |
| XAC_RS08210 | XAC1613 | -2.11566 | hypothetical protein |
| XAC_RS04205 | XAC0816 | -1.16176 | tRNA dihydrouridine synthase DusB |
| XAC_RS05725 | XAC1119 | -1.63416 | hypothetical protein |
| XAC_RS09390 | XAC1847 | -1.52622 | DUF4105 domain-containing protein |
| XAC_RS19085 | - | -2.31289 | hypothetical protein |
| XAC_RS00670 | XAC0130 | -1.82299 | DUF779 domain-containing protein |
| XAC_RS21410 | XAC4245 | -1.56223 | hypothetical protein |
| XAC_RS21395 | - | -1.70288 | aspartate aminotransferase family protein |
| XAC_RS04045 | XAC0784 | 1.11226 | cell division protein FtsZ |
| XAC_RS22220 | XACa0038 | -1.63667 | recombinase family protein |
| XAC_RS19515 | XAC3866 | -1.55569 | hypothetical protein |
| XAC_RS18920 | XAC3744 | -1.79844 | ABC transporter ATP-binding protein |
| XAC_RS02515 | XAC0483 | 2.01751 | CRP-like protein Clp |
| XAC_RS05025 | XAC0982 | 2.77835 | 50S ribosomal protein L14 |
| XAC_RS23555 | - | -1.75007 | IS3 family transposase |
| XAC_RS01675 | XAC0320 | -1.26327 | NAD-dependent protein deacetylase |
| XAC_RS01790 | - | -2.13843 | hypothetical protein |
| XAC_RS19670 | XAC3899 | -1.19093 | anhydro-N-acetylmuramic acid kinase |
| XAC_RS20325 | XAC4035 | -1.39095 | hypothetical protein |
| XAC_RS09465 | - | -1.12904 | hypothetical protein |
| XAC_RS23030 | XAC0820 | -1.61198 | hypothetical protein |
| XAC_RS11670 | XAC2291 | -1.41948 | potassium transporter |
| XAC_RS23730 | - | -1.76283 | hypothetical protein |
| XAC_RS24500 | - | -1.69173 | hypothetical protein |
| XAC_RS23935 | XAC2991 | -1.79769e+308 | hypothetical protein |
| XAC_RS19555 | XAC3875 | -1.06558 | YihY/virulence factor BrkB family protein |
| XAC_RS05080 | XAC0993 | 3.61139 | 30S ribosomal protein S13 |
| XAC_RS14170 | XAC2791 | -1.73649 | LysR family transcriptional regulator |
| XAC_RS19260 | XAC3815 | -1.61189 | DUF3667 domain-containing protein |
| XAC_RS24665 | - | -1.809 | transporter |
| XAC_RS18950 | XAC3750 | -1.79769e+308 | hypothetical protein |
| XAC_RS23725 | - | -1.9 | hypothetical protein |
| XAC_RS06595 | XAC1291 | -1.17335 | hypothetical protein |
| XAC_RS04260 | XAC0828 | -1.6762 | ABC transporter ATP-binding protein |
| XAC_RS21035 | XAC4175 | -1.73026 | glycerophosphodiester phosphodiesterase |
| XAC_RS13705 | XAC2701 | 1.17072 | NADH-quinone oxidoreductase subunit D |
| XAC_RS19370 | XAC3837 | -1.57296 | CRISPR-associated endonuclease Cas3'' |
| XAC_RS11155 | XAC2194 | -1.02087 | hypothetical protein |
| XAC_RS24215 | - | 3.78513 | hypothetical protein |
| XAC_RS09530 | XAC1874 | -1.23906 | LytTR family transcriptional regulator |
| XAC_RS18750 | XAC3707 | -1.21488 | DoxX family protein |
| XAC_RS14850 | XAC2925 | -1.27822 | YggS family pyridoxal phosphate-dependent enzyme |
| XAC_RS03225 | XAC0620 | -1.59944 | sensor histidine kinase |
| XAC_RS07030 | XAC1374 | -1.26821 | DUF2878 domain-containing protein |
| XAC_RS21845 | XAC4331 | -1.53735 | peptidase |
| XAC_RS11850 | XAC2327 | -1.79048 | cytochrome c-type biogenesis protein CcmE 2 |
| tilS | XAC2760 | -1.26034 | tRNA lysidine(34) synthetase TilS |
| XAC_RS21290 | XAC4221 | -1.40285 | alpha/beta hydrolase |
| XAC_RS23210 | - | -1.79769e+308 | hypothetical protein |
| XAC_RS00310 | XAC0060 | -1.59021 | class I SAM-dependent methyltransferase |
| XAC_RS14745 | XAC2905 | 1.97278 | single-stranded DNA-binding protein |
| XAC_RS20135 | XAC3997 | -1.60089 | ABC transporter permease |
| XAC_RS20755 | XAC4119 | -1.54386 | type VI secretion system membrane subunit TssM |
| XAC_RS00845 | XAC0161 | -1.62901 | 2-keto-4-pentenoate hydratase |
| XAC_RS02635 | - | -2.62508 | hypothetical protein |
| XAC_RS12115 | XAC2382 | -1.40706 | GGDEF domain-containing protein |
| XAC_RS20770 | XAC4122 | -1.8997 | type VI secretion system-associated FHA domain protein TagH |
| XAC_RS21675 | XAC4298 | -1.65025 | hypothetical protein |
| XAC_RS22645 | XAC2664 | 3.2074 | type IV pilin protein |
| XAC_RS05845 | XAC1145 | -1.51551 | alpha-2-macroglobulin family protein |
| XAC_RS07925 | XAC1557 | -1.42978 | carbohydrate kinase |
| XAC_RS19435 | XAC3850 | -1.48578 | AcrB/AcrD/AcrF family protein |
| XAC_RS14250 | XAC2807 | -1.45497 | ribonuclease |
| XAC_RS14845 | XAC2924 | 1.10487 | type IV pili twitching motility protein PilT |
| XAC_RS17225 | XAC3400 | -1.38848 | YggW family oxidoreductase |
| XAC_RS20590 | XAC4087 | -1.11099 | hypothetical protein |
| XAC_RS18300 | - | -2.48736 | hypothetical protein |
| XAC_RS18385 | XAC3636 | -1.24189 | hypothetical protein |
| XAC_RS13030 | XAC2558 | -1.41578 | excinuclease Cho |
| mdcE | XAC0563 | -1.50554 | biotin-independent malonate decarboxylase subunit gamma |
| XAC_RS18235 | XAC3606 | 1.76183 | DUF4398 domain-containing protein |
| XAC_RS02455 | XAC0471 | -1.2767 | J domain-containing protein |
| XAC_RS00955 | XAC0184 | -1.37169 | aminotransferase class V-fold PLP-dependent enzyme |
| XAC_RS18930 | XAC3746 | -1.57328 | DUF4142 domain-containing protein |
| XAC_RS03900 | XAC0754 | -1.87678 | type III secretion system effector protein XopI |
| XAC_RS11785 | XAC2314 | -3.47931 | BolA family transcriptional regulator |
| XAC_RS01085 | XAC0207 | -1.83725 | his Kinase A domain protein |
| XAC_RS12680 | XAC2493 | -1.17549 | two-component system response regulator |
| XAC_RS03580 | XAC0690 | -1.90678 | TonB-dependent receptor |
| XAC_RS17335 | XAC3424 | -1.99498 | hypothetical protein |
| XAC_RS00600 | XAC0116 | -1.5134 | membrane protein |
| XAC_RS02300 | XAC0439 | -1.79487 | AcrB/AcrD/AcrF family protein |
| XAC_RS06665 | XAC1305 | -1.75581 | wall-associated protein |
| XAC_RS05955 | XAC1167 | -1.36738 | hypothetical protein |
| cas8c | - | -1.48381 | type I-C CRISPR-associated protein Cas8c/Csd1 |
| XAC_RS05170 | XAC1011 | -1.3782 | zinc-binding alcohol dehydrogenase family protein |
| XAC_RS00835 | XAC0159 | -1.62987 | carboxylesterase/lipase family protein |
| sdhC | XAC2075 | 1.43114 | succinate dehydrogenase, cytochrome b556 subunit |
| modC | XAC3360 | -1.36427 | molybdenum ABC transporter ATP-binding protein |
| XAC_RS22460 | XACb0048 | 1.06026 | hypothetical protein |
| yeiP | XAC1849 | 1.48717 | elongation factor P-like protein YeiP |
| XAC_RS12285 | XAC2416 | 3.21315 | virulence regulator |
| XAC_RS16205 | XAC3197 | -1.48715 | aliphatic sulfonate ABC transporter permease SsuC |
| XAC_RS14220 | XAC2801 | -1.7067 | MipA/OmpV family protein |
| XAC_RS00725 | XAC0139 | 1.60546 | hypothetical protein |
| XAC_RS14390 | XAC2834 | -1.29987 | hypothetical protein |
| XAC_RS03430 | XAC0661 | -1.5773 | endopolygalacturonase |
| XAC_RS20785 | XAC4125 | -1.37985 | tetratricopeptide repeat protein |
| XAC_RS11855 | XAC2328 | -1.30555 | heme lyase CcmF/NrfE family subunit |
| XAC_RS19170 | XAC3797 | -1.31838 | O-antigen ligase family protein |
| XAC_RS14140 | XAC2784 | -1.13263 | DUF4442 domain-containing protein |
| XAC_RS00360 | XAC0070 | -1.37879 | hypothetical protein |
| XAC_RS10315 | XAC2028 | -1.65642 | glutathione-dependent formaldehyde dehydrogenase |
| XAC_RS06460 | XAC1266 | -1.06198 | AraC family transcriptional regulator |
| XAC_RS01700 | XAC0325 | -1.47054 | DNA-binding response regulator |
| XAC_RS09370 | XAC1843 | -1.1002 | NUDIX domain-containing protein |
| XAC_RS19180 | XAC3799 | -1.27822 | ribosomal RNA small subunit methyltransferase B |
| XAC_RS02790 | XAC0536 | -1.23479 | AraC family transcriptional regulator |
| XAC_RS17540 | XAC3465 | -1.63784 | lauroyl acyltransferase |
| XAC_RS15630 | - | -1.58425 | carbohydrate kinase family protein |
| XAC_RS06875 | XAC1345 | -1.21258 | GGDEF domain-containing protein |
| XAC_RS10135 | XAC1994 | -1.01942 | PAS domain S-box protein |
| XAC_RS03150 | XAC0605 | -1.42494 | DUF72 domain-containing protein |
| XAC_RS07775 | XAC1527 | -1.51116 | hypothetical protein |
| XAC_RS00450 | XAC0088 | -1.63373 | NAD(P)H oxidoreductase |
| XAC_RS05895 | XAC1156 | 1.03974 | protease modulator HflC |
| XAC_RS11545 | XAC2270 | -1.75257 | TIGR03745 family integrating conjugative element membrane protein |
| XAC_RS05985 | XAC1173 | -1.67975 | TetR/AcrR family transcriptional regulator |
| XAC_RS14360 | XAC2828 | -1.1633 | MerC domain-containing protein |
| XAC_RS00605 | XAC0117 | -1.47515 | DUF58 domain-containing protein |
| XAC_RS00850 | XAC0162 | -1.47153 | C4-dicarboxylate ABC transporter |
| XAC_RS04305 | XAC0837 | -1.58142 | diacylglycerol kinase |
| XAC_RS00210 | XAC0041 | -1.33489 | glycosyltransferase family 1 protein |
| cysW | XAC1019 | -1.37867 | sulfate ABC transporter permease subunit CysW |
| XAC_RS11295 | XAC2223 | -1.53691 | DUF1016 domain-containing protein |
| XAC_RS07290 | XAC1425 | -1.39495 | fimbrial biogenesis outer membrane usher protein |
| XAC_RS15580 | XAC3070 | -1.11203 | glucokinase |
| XAC_RS00425 | XAC0082 | -1.31517 | KR domain-containing protein |
| XAC_RS01715 | XAC0328 | -1.70534 | multidrug efflux RND transporter permease subunit |
| XAC_RS14165 | XAC2790 | -1.4189 | EamA family transporter |
| XAC_RS24745 | - | -2.00516 | hypothetical protein |
| XAC_RS03735 | XAC0722 | 1.22418 | thiol:disulfide interchange protein DsbA/DsbL |
| XAC_RS06880 | XAC1346 | 1.06271 | hypothetical protein |
| XAC_RS11530 | - | -1.79769e+308 | hypothetical protein |
| XAC_RS17665 | XAC3490 | -1.57285 | amylosucrase |
| XAC_RS22650 | XAC2666 | 2.05246 | pilus assembly protein |
| XAC_RS24590 | XAC4247 | -2.10822 | hypothetical protein |
| XAC_RS23280 | XAC1508 | 1.67478 | hypothetical protein |
| pabB | XAC1130 | -1.3221 | aminodeoxychorismate synthase, component I |
| XAC_RS09385 | XAC1846 | 1.96715 | DUF2388 domain-containing protein |
| XAC_RS11120 | XAC2187 | -1.05808 | PIN domain-containing protein |
| XAC_RS22360 | - | -1.31962 | hypothetical protein |
| XAC_RS16795 | XAC3315 | -1.18237 | carboxylesterase |
| XAC_RS12880 | XAC2529 | -1.67505 | DUF4329 domain-containing protein |
| XAC_RS01550 | XAC0296 | -1.58835 | NAD(P)/FAD-dependent oxidoreductase |
| XAC_RS24145 | XAC3371 | -1.27003 | alpha/beta hydrolase |
| XAC_RS14480 | XAC2852 | -1.51387 | cell envelope integrity protein CreD |
| XAC_RS01460 | XAC0280 | -1.33183 | cell division protein ZapE |
| XAC_RS06020 | - | -1.46478 | short-chain dehydrogenase |
| XAC_RS06690 | XAC1309 | -1.54429 | arabinogalactan endo-1,4-beta-galactosidase |
| XAC_RS13310 | XAC2616 | 2.88203 | hypothetical protein |
| XAC_RS07105 | XAC1389 | -1.12372 | ABC transporter ATP-binding protein |
| XAC_RS08595 | XAC1686 | -1.39161 | cytochrome-c oxidase |
