# Supplementary material for: RNA-seq analysis provides insights into cold stress responses of Xanthomonas citri pv. citri
Source: BMC Genomics. 2019 Nov 6;20:807. doi: 10.1186/s12864-019-6193-0 (PMC6833247; doi:10.1186/s12864-019-6193-0)
Supplement: Supplementary file 4 — Additional file 4: Table S4. List of genes related to carbohydrate metabolic process in Xcc regulated by temperature. [file 12864_2019_6193_MOESM4_ESM.docx]

**Table S4. List of genes related to carbohydrate metabolic process in *Xcc* regulated by temperature**

| Gene ID | Gene name | log2 fold change (15°C/ 28°C) | Gene Description |
| --- | --- | --- | --- |
| XAC_RS16950 | XAC3344 | 2.04251 | fructose-bisphosphate aldolase class I |
| XAC_RS08760 | XAC1719 | 1.81046 | enolase |
| XAC_RS03190 | XAC0612 | 1.1083 | endoglucanase |
| XAC_RS00640 | XAC0124 | 1.07656 | fructose 1,6-bisphosphatase |
| XAC_RS10380 | XAC2041 | 2.7043 | phosphoenolpyruvate synthase |
| XAC_RS05150 | XAC1006 | 2.62614 | malate dehydrogenase |
| XAC_RS18100 | XAC3580 | 1.16546 | mannose-1-phosphate guanylyltransferase/ mannose-6-phosphate isomerase |
| XAC_RS02230 | XAC0426 | -1.02095 | 1,4-alpha-glucan branching enzyme |
| XAC_RS12900 | XAC2533 | -1.53051 | glycosyl hydrolase family 43 |
| XAC_RS21435 | XAC4249 | -1.46388 | endo-1,4-beta-xylanase |
| XAC_RS04085 | - | -1.60108 | hypothetical protein |
| XAC_RS02240 | XAC0428 | -1.24056 | 4-alpha-glucanotransferase |
| XAC_RS00145 | XAC0028 | -1.53947 | cellulase |
| XAC_RS21405 | XAC4244 | -1.60103 | xylulokinase |
| XAC_RS06015 | XAC1179 | -1.0402 | glycoside hydrolase family 92 protein |
| XAC_RS15350 | XAC3023 | -1.63987 | DUF1906 domain-containing protein |
| XAC_RS09025 | XAC1771 | -1.49575 | 9-O-acetylesterase |
| XAC_RS17785 | XAC3518 | -1.76031 | UDP-forming cellulose synthase catalytic subunit |
| XAC_RS19670 | XAC3899 | -1.19093 | anhydro-N-acetylmuramic acid kinase |
| XAC_RS21085 | XAC4183 | -1.64831 | xylosidase |
| XAC_RS15650 | XAC3084 | -1.27269 | beta-galactosidase |
| XAC_RS21450 | - | -1.96452 | 1,4-beta-xylanase |
| XAC_RS21340 | XAC4230 | -1.64478 | glycoside hydrolase family 43 protein |
| XAC_RS12835 | XAC2522 | -1.01744 | cellulase |
| XAC_RS16380 | XAC3230 | -1.68284 | hypothetical protein |
| XAC_RS21445 | XAC4251 | -1.29778 | uronate isomerase |
| XAC_RS15635 | XAC3081 | -1.84599 | 6-phospho-beta-glucosidase |
| XAC_RS17705 | XAC3499 | -1.16656 | polysaccharide deacetylase family protein |
| XAC_RS04785 | - | -1.92483 | hypothetical protein |
| XAC_RS06000 | XAC1176 | -1.29881 | glycosyl hydrolase |
| XAC_RS21965 | XAC4355 | -1.30667 | glycoside hydrolase family 92 protein |
| XAC_RS19715 | XAC3908 | -1.27681 | KR domain-containing protein |
| XAC_RS15610 | XAC3076 | -1.06213 | glycoside hydrolase family 3 |
| XAC_RS02205 | XAC0421 | -1.67491 | phosphoglycerol transferase I |
| XAC_RS21330 | XAC4228 | -1.53127 | 9-O-acetylesterase |
| XAC_RS15580 | XAC3070 | -1.11203 | glucokinase |
| XAC_RS21465 | XAC4254 | -1.40364 | beta-1,4-xylanase |
| XAC_RS08640 | XAC1695 | -1.85255 | hypothetical protein |
| XAC_RS00820 | XAC0156 | -1.15695 | 1,4-alpha-glucan branching protein GlgB |
| XAC_RS06555 | XAC1285 | -1.06752 | glycoside hydrolase family 16 protein |
| XAC_RS16780 | XAC3312 | -1.2626 | beta-galactosidase |
| XAC_RS17810 | XAC3522 | -1.36964 | hypothetical protein |
| XAC_RS23645 | XAC2373 | -1.0864 | pectate lyase |
| XAC_RS15060 | XAC2967 | -1.33257 | KpsF/GutQ family sugar-phosphate isomerase |
| XAC_RS21485 | XAC4258 | -1.61469 | alpha-N-arabinofuranosidase |
| XAC_RS17735 | XAC3505 | -1.23768 | rhamnogalacturonase B |
| XAC_RS21440 | XAC4250 | -1.52679 | DUF4982 domain-containing protein |
| XAC_RS10615 | XAC2088 | -1.23827 | tetraacyldisaccharide 4'-kinase |
| XAC_RS17770 | XAC3515 | -1.58074 | cellulose synthase operon protein C |
| XAC_RS15155 | XAC2986 | -1.71018 | pectate lyase |
| XAC_RS06690 | XAC1309 | -1.54429 | arabinogalactan endo-1,4-beta-galactosidase |
| XAC_RS03665 | - | -1.74589 | beta-galactosidase |
| XAC_RS00840 | XAC0160 | -1.34623 | alpha/beta hydrolase |
| XAC_RS21660 | XAC4296 | -1.03656 | lytic murein transglycosylase |
| XAC_RS18625 | XAC3681 | -1.56415 | sorbosone dehydrogenase |
| XAC_RS10620 | XAC2089 | -1.28025 | 3-deoxy-manno-octulosonate cytidylyltransferase |
| XAC_RS21160 | XAC4195 | -1.26771 | NdvB protein |
| XAC_RS21170 | XAC4197 | -1.46749 | gluconokinase |
| XAC_RS15820 | XAC3120 | -1.12106 | glucokinase |
| XAC_RS21345 | XAC4231 | -1.65668 | glucan 1,4-alpha-glucosidase |
| XAC_RS22040 | XAC4371 | -1.64658 | tetratricopeptide repeat protein |
| XAC_RS15620 | XAC3078 | -1.78927 | beta-galactosidase |
| XAC_RS02930 | XAC0562 | -1.27728 | biotin-independent malonate decarboxylase subunit beta |
| XAC_RS17775 | XAC3516 | -1.50494 | cellulase |
| XAC_RS01335 | XAC0256 | -1.37391 | malate synthase A |
| XAC_RS20440 | XAC4058 | -1.64765 | beta-xylosidase |
| XAC_RS09035 | XAC1773 | -1.24752 | DUF5110 domain-containing protein |
| XAC_RS18915 | XAC3743 | -1.7829 | beta-glucosidase |
| XAC_RS17665 | XAC3490 | -1.57285 | amylosucrase |
| XAC_RS02995 | XAC0575 | -1.55346 | arabinogalactan endo-1,4-beta-galactosidase |
| XAC_RS00875 | XAC0168 | -1.1495 | 5-keto-4-deoxyuronate isomerase |
| XAC_RS12075 | XAC2374 | -1.60458 | polygalacturonase |
| XAC_RS18435 | XAC3645 | -2.01999 | GtrA family protein |
| XAC_RS06685 | XAC1308 | -1.44898 | DUF4982 domain-containing protein |
| XAC_RS17780 | XAC3517 | -1.6299 | divalent ion tolerance protein CutA |
| XAC_RS15645 | XAC3083 | -1.24878 | hypothetical protein |
| XAC_RS19825 | XAC3928 | -1.23355 | beta-N-acetylglucosaminidase |
| XAC_RS08990 | XAC1765 | -1.54028 | D-galactonate dehydratase |
| XAC_RS13160 | XAC2584 | -1.02679 | GumC protein |
| XAC_RS21325 | XAC4227 | -1.69802 | alpha-glucuronidase |
| XAC_RS17540 | XAC3465 | -1.63784 | lauroyl acyltransferase |
| XAC_RS19345 | XAC3832 | -1.65482 | bifunctional isocitrate dehydrogenase kinase/phosphatase |
| XAC_RS00865 | XAC0165 | -1.6702 | beta-xylosidase |
| XAC_RS12905 | XAC2534 | -1.64815 | carbohydrate-binding protein |
| XAC_RS03430 | XAC0661 | -1.5773 | endopolygalacturonase |
| XAC_RS06505 | XAC1275 | -1.53282 | glycoside hydrolase family 43 protein |
| XAC_RS18010 | XAC3562 | -1.63367 | pectate lyase |
| XAC_RS08980 | XAC1763 | -1.14403 | MFS transporter |
| XAC_RS07200 | XAC1408 | -1.14153 | lipid-A-disaccharide synthase |
| XAC_RS19725 | XAC3910 | -1.59088 | membrane protein |
| XAC_RS01565 | XAC0299 | -1.97237 | allantoinase PuuE |
| XAC_RS17560 | XAC3469 | -1.26581 | glycoside hydrolase family 92 protein |
| XAC_RS04190 | XAC0813 | 2.68784 | S-adenosylmethionine synthase |
| XAC_RS04145 | XAC0804 | 1.76915 | adenosylhomocysteinase |
| XAC_RS20550 | XAC4079 | -1.92245 | a-type carbonic anhydrase |
| XAC_RS01730 | XAC0331 | -1.28508 | 5,10-methylenetetrahydrofolate reductase |
| XAC_RS24500 |  | -1.69173 | hypothetical protein |
