# Supplementary material for: RNA-seq analysis provides insights into cold stress responses of Xanthomonas citri pv. citri
Source: BMC Genomics. 2019 Nov 6;20:807. doi: 10.1186/s12864-019-6193-0 (PMC6833247; doi:10.1186/s12864-019-6193-0)
Supplement: Supplementary file 5 — Additional file 5: Table S5. List of genes related to cellular nitrogen compound biosynthetic process in Xcc regulated by temperature. [file 12864_2019_6193_MOESM5_ESM.docx]

**Table S5. List of genes related to cellular nitrogen compound biosynthetic process in *Xcc* regulated by temperature**

| Gene ID | Gene name | log2 fold change (15°C/ 28°C) | Gene Description |
| --- | --- | --- | --- |
| XAC_RS17195 | XAC3394 | 2.77922 | DNA-directed RNA polymerase subunit omega |
| XAC_RS09100 | XAC1787 | 2.21198 | aspartate 1-decarboxylase |
| XAC_RS02450 | XAC0470 | 2.0739 | phosphoribosylaminoimidazolesuccinocarboxamide synthase |
| XAC_RS02520 | XAC0484 | 2.02871 | S-adenosylmethionine decarboxylase proenzyme |
| XAC_RS10250 | XAC2015 | 3.51401 | nucleoside-diphosphate kinase |
| XAC_RS09460 | XAC1861 | 1.03661 | carbamoyl-phosphate synthase small subunit |
| XAC_RS18455 | XAC3650 | 1.15835 | ATP synthase subunit gamma |
| XAC_RS18475 | XAC3654 | 2.34929 | F0F1 ATP synthase subunit C |
| XAC_RS05095 | XAC0996 | 2.53305 | DNA-directed RNA polymerase subunit alpha |
| XAC_RS11655 | XAC2288 | 1.45156 | IMP dehydrogenase |
| XAC_RS18460 | XAC3651 | 1.63249 | ATP synthase subunit alpha |
| XAC_RS17400 | XAC3437 | 2.10944 | adenylate kinase |
| XAC_RS18470 | XAC3653 | 1.63268 | ATP synthase subunit B |
| XAC_RS04190 | XAC0813 | 2.68784 | S-adenosylmethionine synthase |
| XAC_RS20350 | XAC4040 | 1.13472 | porphobilinogen synthase |
| XAC_RS08155 | XAC1603 | -1.30825 | 3-hydroxyanthranilate 3,4-dioxygenase |
| XAC_RS15805 | XAC3117 | -1.51262 | coenzyme PQQ synthesis protein E |
| XAC_RS16460 | XAC3244 | -1.67563 | dephospho-CoA kinase |
| XAC_RS16460 | XAC3244 | -1.67563 | dephospho-CoA kinase |
| XAC_RS01130 | XAC0216 | -1.32601 | uroporphyrinogen III methyltransferase |
| XAC_RS00065 | XAC0012 | -1.12926 | pyridoxine 5'-phosphate synthase |
| XAC_RS10620 | XAC2089 | -1.28025 | 3-deoxy-manno-octulosonate cytidylyltransferase |
| XAC_RS20800 | XAC4128 | -1.70013 | DNA-directed RNA polymerase sigma-70 factor |
| XAC_RS07755 | XAC1524 | -1.04631 | pyridoxal kinase |
| XAC_RS04840 | - | -1.2772 | glutamyl-tRNA reductase |
| XAC_RS15570 | XAC3068 | -1.18232 | adenosylmethionine--8-amino-7-oxononanoate transaminase |
| XAC_RS18320 | XAC3622 | -1.62054 | DNA polymerase IV |
| XAC_RS19275 | XAC3818 | -1.64442 | primosomal protein N' |
| XAC_RS23585 | - | -1.27123 | uroporphyrin-III C-methyltransferase |
| XAC_RS01350 | XAC0259 | -1.50999 | putative peptide modification system cyclase |
| XAC_RS04070 | XAC0789 | -1.35642 | 8-oxo-dGTP diphosphatase MutT |
| XAC_RS15795 | XAC3115 | -1.07951 | pyrroloquinoline-quinone synthase |
| XAC_RS15790 | XAC3114 | -1.05695 | pyrroloquinoline quinone biosynthesis protein B |
| XAC_RS23250 | - | -1.55466 | asparagine synthase |
| XAC_RS18020 | XAC3564 | -1.06696 | hypothetical protein |
| XAC_RS15910 | XAC3139 | -1.29781 | 7-carboxy-7-deazaguanine synthase QueE |
| XAC_RS09070 | XAC1781 | -1.69477 | GTP cyclohydrolase I FolE2 |
| XAC_RS15085 | XAC2972 | -1.13802 | RNA polymerase sigma-54 factor |
| XAC_RS05485 | XAC1072 | -1.58932 | phage-related DNA-directed RNA polymerase |
| XAC_RS16155 | XAC3187 | -1.71529 | bifunctional adenosylcobinamide kinase/ adenosylcobinamide-phosphate guanylyltransferase |
| XAC_RS20495 | XAC4068 | -1.31152 | 2-dehydropantoate 2-reductase |
| XAC_RS08110 | XAC1595 | -1.01892 | NAD(+) kinase |
| XAC_RS02620 | XAC0504 | -1.43545 | membrane protein |
| XAC_RS20805 | XAC4129 | -1.55266 | RNA polymerase sigma factor |
| XAC_RS17225 | XAC3400 | -1.38848 | YggW family oxidoreductase |
| XAC_RS05260 | XAC1029 | -1.1127 | bifunctional tetrahydrofolate synthase/  dihydrofolate synthase |
| XAC_RS08570 | XAC1682 | -1.24204 | DNA-directed RNA polymerase sigma-70 factor |
| XAC_RS10970 | XAC2159 | -1.33876 | siroheme synthase |
| XAC_RS16120 | XAC3180 | -1.72419 | iron transporter |
| XAC_RS23245 | XAC1490 | -1.86711 | hypothetical protein |
| XAC_RS17450 | XAC3447 | -1.60588 | phosphomethylpyrimidine synthase |
| XAC_RS20095 | XAC3989 | -1.63503 | RNA polymerase sigma factor |
| XAC_RS20710 | XAC4110 | -1.03616 | DNA polymerase I |
| XAC_RS16110 | XAC3178 | -1.47718 | IucA/IucC family siderophore biosynthesis protein |
| XAC_RS16160 | XAC3188 | -1.62812 | cobyric acid synthase CobQ |
| XAC_RS19575 | XAC3879 | -1.54925 | protoheme IX farnesyltransferase |
| XAC_RS02160 | XAC0412 | -1.83484 | EscN/YscN/HrcN family type III secretion system ATPase |
| XAC_RS14105 | XAC2778 | -1.67582 | nicotinate-nucleotide adenylyltransferase |
| XAC_RS04730 | XAC0922 | -1.58349 | RNA polymerase sigma factor |
| XAC_RS16170 | XAC3190 | -1.39032 | cobalamin biosynthesis protein |
| XAC_RS20235 | XAC4017 | -1.12462 | type III pantothenate kinase |
| XAC_RS16140 | XAC3184 | -1.63339 | adenosylcobinamide-GDP ribazoletransferase |
| XAC_RS19740 | XAC3913 | -1.04834 | deoxyuridine 5'-triphosphate nucleotidohydrolase |
| XAC_RS19690 | XAC3903 | -1.17325 | orotate phosphoribosyltransferase |
| XAC_RS07190 | XAC1406 | -1.11662 | DNA polymerase III subunit alpha |
| XAC_RS14110 | XAC2779 | -1.4517 | DNA polymerase III subunit delta |
| XAC_RS08140 | XAC1600 | -1.49149 | kynurenine 3-monooxygenase |
| XAC_RS14290 | XAC2814 | -1.64311 | sigma-70 family RNA polymerase sigma factor |
| XAC_RS00305 | XAC0059 | -1.55273 | asparagine synthetase B |
| XAC_RS13785 | XAC2716 | -1.17594 | tryptophan synthase subunit alpha |
| XAC_RS19560 | XAC3876 | -1.34365 | DNA primase |
| XAC_RS16165 | XAC3189 | -1.91566 | threonine-phosphate decarboxylase |
| XAC_RS21285 | XAC4220 | -1.06758 | Ferrochelatase |
