# Supplementary material for: RNA-seq analysis provides insights into cold stress responses of Xanthomonas citri pv. citri
Source: BMC Genomics. 2019 Nov 6;20:807. doi: 10.1186/s12864-019-6193-0 (PMC6833247; doi:10.1186/s12864-019-6193-0)
Supplement: Supplementary file 6 — Additional file 6: Table S6. List of genes related to flagellum in Xcc regulated by temperature. [file 12864_2019_6193_MOESM6_ESM.docx]

**Table S6. List of genes related to flagellum in *Xcc* regulated by temperature**

| Gene ID | Gene name | log2 fold change (15°C/ 28°C) | Gene Description |
| --- | --- | --- | --- |
| XAC_RS09830 | XAC1931 | 2.49681 | chemotaxis protein |
| XAC_RS10085 | XAC1983 | 1.27924 | flagellar hook protein FlgE |
| XAC_RS10045 | XAC1975 | 4.40245 | flagellin |
| XAC_RS10040 | XAC1974 | 2.21335 | flagellar protein |
| XAC_RS09950 | XAC1955 | -1.81915 | flagellar hook-basal body complex protein FliE |
| XAC_RS10095 | XAC1985 | -1.17699 | flagellar basal body rod protein FlgC |
