# Supplementary material for: RNA-seq analysis provides insights into cold stress responses of Xanthomonas citri pv. citri
Source: BMC Genomics. 2019 Nov 6;20:807. doi: 10.1186/s12864-019-6193-0 (PMC6833247; doi:10.1186/s12864-019-6193-0)
Supplement: Supplementary file 7 — Additional file 7: Table S7. List of genes related to pilus organization in Xcc regulated by temperature. [file 12864_2019_6193_MOESM7_ESM.docx]

**Table S7. List of genes related to pilus organization in *Xcc* regulated by temperature**

| Gene ID | Gene name | log2 fold change (15°C/ 28°C) | Gene Description |
| --- | --- | --- | --- |
| XAC_RS13545 | XAC2667 | 2.5596 | prepilin-type cleavage/methylation domain-containing protein |
| XAC_RS17130 | XAC3381 | 2.55871 | type IV pilus secretin PilQ |
