# Supplementary material for: RNA-seq analysis provides insights into cold stress responses of Xanthomonas citri pv. citri
Source: BMC Genomics. 2019 Nov 6;20:807. doi: 10.1186/s12864-019-6193-0 (PMC6833247; doi:10.1186/s12864-019-6193-0)
Supplement: Supplementary file 8 — Additional file 8: Table S8. List of genes related to fatty acid metabolism in Xcc regulated by temperature. [file 12864_2019_6193_MOESM8_ESM.docx]

**Table S8. List of genes related to fatty acid metabolism in *Xcc* regulated by temperature**

1. List of genes related to fatty acid metabolic and biosynthetic process in *Xcc* regulated by temperature

| Gene ID | Gene name | log2 fold change (15°C/ 28°C) | Gene Description |
| --- | --- | --- | --- |
| XAC_RS05765 | XAC1128 | 3.66436 | acyl carrier protein |
| FadL | XAC0019 | 2.1504 | long-chain fatty acid transport protein |
| XAC_RS05810 | XAC1137 | 1.26353 | methylisocitrate lyase |
| XAC_RS00735 | XAC0141 | 1.33486 | enoyl-[acyl-carrier-protein |
| XAC_RS13095 | XAC2571 | -1.39495 | ketoacyl-ACP synthase III |
| XAC_RS05805 | XAC1136 | -1.41014 | propionate catabolism operon regulatory protein PrpR |
| XAC_RS20610 | XAC4090 | -1.1502 | 3-oxoacyl-ACP reductase FabG |

1. List of genes related to phospholipid biosynthetic process in *Xcc* regulated by temperature

| Gene ID | Gene name | log2 fold change (15 ^o^C/ 28 ^o^C) | Gene Description |
| --- | --- | --- | --- |
| XAC_RS04855 | XAC0948 | -1.25513 | 4-diphosphocytidyl-2C-methyl-D-erythritol kinase |
| XAC_RS07235 | XAC1415 | -1.20328 | 1-deoxy-D-xylulose-5-phosphate reductoisomerase |
| XAC_RS10405 | XAC2046 | -1.12446 | CDP-diacylglycerol--serine O-phosphatidyltransferase |
| XAC_RS02680 | XAC0514 | -1.44726 | CDP-alcohol phosphatidyltransferase family protein |
| XAC_RS08770 | XAC1721 | -1.06765 | 2-C-methyl-D-erythritol 4-phosphate cytidylyltransferase |
| XAC_RS07200 | XAC1408 | -1.14153 | lipid-A-disaccharide synthase |
| XAC_RS10615 | XAC2088 | -1.23827 | tetraacyldisaccharide 4'-kinase |
| XAC_RS02705 | XAC0519 | -1.30964 | CDP-alcohol phosphatidyltransferase family protein |
| XAC_RS17540 | XAC3465 | -1.63784 | lauroyl acyltransferase |
| XAC_RS07240 | XAC1416 | -1.41494 | phosphatidate cytidylyltransferase |
| XAC_RS08775 | XAC1722 | -1.15875 | 2-C-methyl-D-erythritol 2,4-cyclodiphosphate synthase |
| XAC_RS20655 | XAC4099 | -1.2821 | 1-acyl-sn-glycerol-3-phosphate acyltransferase |
| XAC_RS04305 | XAC0837 | -1.58142 | diacylglycerol kinase |
| XAC_RS19725 | XAC3910 | -1.59088 | membrane protein |

1. List of genes related to lipid metabolic and biosynthetic in *Xcc* regulated by temperature

| Gene ID | Gene name | log2 fold change (15°C/ 28°C) | Gene Description |
| --- | --- | --- | --- |
| XAC_RS00735 | XAC0141 | 1.33486 | enoyl-[acyl-carrier-protein |
| XAC_RS05810 | XAC1137 | 1.26353 | methylisocitrate lyase |
| XAC_RS07480 | XAC1463 | 1.53766 | phospholipase |
| XAC_RS05765 | XAC1128 | 3.66436 | acyl carrier protein |
| XAC_RS05240 | XAC1024 | -1.68199 | phospholipase C, phosphocholine-specific |
| XAC_RS22020 | XAC4367 | -1.75723 | glycerophosphoryl diester phosphodiesterase |
| XAC_RS10620 | XAC2089 | -1.28025 | 3-deoxy-manno-octulosonate cytidylyltransferase |
| XAC_RS08775 | XAC1722 | -1.15875 | 2-C-methyl-D-erythritol 2,4-cyclodiphosphate synthase |
| XAC_RS20655 | XAC4099 | -1.2821 | 1-acyl-sn-glycerol-3-phosphate acyltransferase |
| XAC_RS07035 | XAC1375 | -1.16214 | class I SAM-dependent methyltransferase |
| XAC_RS08770 | XAC1721 | -1.06765 | 2-C-methyl-D-erythritol 4-phosphate cytidylyltransferase |
| XAC_RS10615 | XAC2088 | -1.23827 | tetraacyldisaccharide 4'-kinase |
| XAC_RS07240 | XAC1416 | -1.41494 | phosphatidate cytidylyltransferase |
| XAC_RS00395 | XAC0076 | -1.77141 | avirulence protein |
| XAC_RS15180 | - | -1.72835 | hypothetical protein |
| XAC_RS07235 | XAC1415 | -1.20328 | 1-deoxy-D-xylulose-5-phosphate reductoisomerase |
| XAC_RS19955 | XAC3959 | -1.13401 | acyl-CoA desaturase |
| XAC_RS00920 | XAC0177 | -1.50495 | patatin |
| XAC_RS21525 | XAC4270 | -1.04206 | glycerol-3-phosphate 1-O-acyltransferase PlsB |
| XAC_RS14025 | XAC2762 | -1.13358 | polyprenyl synthetase family protein |
| XAC_RS17540 | XAC3465 | -1.63784 | lauroyl acyltransferase |
| XAC_RS13160 | XAC2584 | -1.02679 | GumC protein |
| XAC_RS05805 | XAC1136 | -1.41014 | propionate catabolism operon regulatory protein PrpR |
| XAC_RS20610 | XAC4090 | -1.1502 | 3-oxoacyl-ACP reductase FabG |
| XAC_RS10405 | XAC2046 | -1.12446 | CDP-diacylglycerol--serine O-phosphatidyltransferase |
| XAC_RS24020 | XAC3159 | -1.68531 | phospholipase C |
| XAC_RS02605 | XAC0501 | -1.78364 | hypothetical protein |
| XAC_RS06000 | XAC1176 | -1.29881 | glycosyl hydrolase |
| XAC_RS04855 | XAC0948 | -1.25513 | 4-diphosphocytidyl-2C-methyl-D-erythritol kinase |
| XAC_RS07025 | XAC1373 | -1.44836 | DUF1295 domain-containing protein |
| XAC_RS02705 | XAC0519 | -1.30964 | CDP-alcohol phosphatidyltransferase family protein |
| XAC_RS04785 | - | -1.92483 | hypothetical protein |
| XAC_RS04305 | XAC0837 | -1.58142 | diacylglycerol kinase |
| XAC_RS19725 | XAC3910 | -1.59088 | membrane protein |
| XAC_RS17050 | XAC3365 | -1.77356 | hypothetical protein |
| XAC_RS13095 | XAC2571 | -1.39495 | ketoacyl-ACP synthase III |
| XAC_RS02680 | XAC0514 | -1.44726 | CDP-alcohol phosphatidyltransferase family protein |
| XAC_RS16015 | XAC3160 | -1.63123 | phospholipase C, phosphocholine-specific |
| XAC_RS07200 | XAC1408 | -1.14153 | lipid-A-disaccharide synthase |
| XAC_RS05855 | XAC1147 | -1.4514 | glycerophosphodiester phosphodiesterase |
| XAC_RS07050 | XAC1378 | -1.47014 | acyl-CoA desaturase |
| XAC_RS21035 | XAC4175 | -1.73026 | glycerophosphodiester phosphodiesterase |
| XAC_RS08640 | XAC1695 | -1.85255 | hypothetical protein |
