# Supplementary material for: RNA-seq analysis provides insights into cold stress responses of Xanthomonas citri pv. citri
Source: BMC Genomics. 2019 Nov 6;20:807. doi: 10.1186/s12864-019-6193-0 (PMC6833247; doi:10.1186/s12864-019-6193-0)
Supplement: Supplementary file 9 — Additional file 9: Table S9. List of genes related to Xcc pathogenesis regulated by temperature. [file 12864_2019_6193_MOESM9_ESM.docx]

**Table S9. List of genes related to pathogenesis in *Xcc* regulated by temperature**

| Gene ID | Gene name | log2 fold change (15°C/ 28°C) | Gene Description |
| --- | --- | --- | --- |
| XAC_RS02515 | XAC0483 | 2.01751 | CRP-like protein Clp |
| XAC_RS17935 | XAC3548 | -1.59656 | adhesin |
| XAC_RS17925 | XAC3546 | -1.68247 | membrane protein |
| XAC_RS22150 | XACa0022 | -1.79446 | avirulence protein |
| XAC_RS02145 | XAC0409 | -1.73278 | EscJ/YscJ/HrcJ family type III secretion inner membrane ring protein |
| XAC_RS22535 | XACb0065 | -1.90554 | avirulence protein |
