# Supplementary material for: RNA-seq analysis provides insights into cold stress responses of Xanthomonas citri pv. citri
Source: BMC Genomics. 2019 Nov 6;20:807. doi: 10.1186/s12864-019-6193-0 (PMC6833247; doi:10.1186/s12864-019-6193-0)
Supplement: Supplementary file 10 — Additional file 10: Table S10. List of genes related to Xcc secretion regulated by temperature. [file 12864_2019_6193_MOESM10_ESM.docx]

**Table S10. List of genes related to secretion in *Xcc* regulated by temperature**

| Gene ID | Gene name | log2 fold change (15°C/ 28°C) | Gene Description |
| --- | --- | --- | --- |
| XAC_RS16450 | XAC3242 | 1.20786 | type II secretion system F family protein |
| XAC_RS13290 | XAC2612 | 1.2437 | VirB6 protein |
| XAC_RS13550 | XAC2669 | 2.19278 | prepilin-type N-terminal cleavage/methylation domain-containing protein |
| XAC_RS17130 | XAC3381 | 2.55871 | type IV pilus secretin PilQ |
| XAC_RS13730 | XAC2706 | 2.29523 | preprotein translocase subunit SecG |
| XAC_RS03640 | XAC0702 | -1.79365 | type II secretion system protein K |
| XAC_RS02125 | XAC0405 | -1.74432 | hypersensitivity response secretion protein hrcV |
| XAC_RS17910 | XAC3543 | -1.54189 | type II secretion system F family protein |
| XAC_RS09930 | XAC1951 | -1.27966 | FliI/YscN family ATPase |
| XAC_RS09890 | XAC1942 | -0.74375 | flagellar biosynthesis |
| XAC_RS02145 | XAC0409 | -1.73278 | EscJ/YscJ/HrcJ family type III secretion inner membrane ring protein |
| XAC_RS23765 | - | -1.61488 | hypothetical protein |
| XAC_RS02115 | XAC0403 | -1.71806 | YscQ/HrcQ family type III secretion apparatus protein |
| XAC_RS02110 | XAC0402 | -1.84558 | EscR/YscR/HrcR family type III secretion system export apparatus protein |
| XAC_RS11185 | XAC2201 | -1.86914 | HlyD family type I secretion periplasmic adaptor subunit |
| XAC_RS02175 | XAC0415 | -1.69825 | EscC/YscC/HrcC family type III secretion system outer membrane ring protein |
| XAC_RS17885 | XAC3538 | -1.74152 | general secretion pathway protein GspK |
| XAC_RS02160 | XAC0412 | -1.83484 | EscN/YscN/HrcN family type III secretion system ATPase |
| XAC_RS21275 | XAC4218 | 0.382804 | twin-arginine translocase subunit TatA |
| XAC_RS04910 | XAC0959 | -1.77376 | preprotein translocase subunit SecE |
| XAC_RS02130 | XAC0406 | -1.97242 | EscU/YscU/HrcU family type III secretion system export apparatus switch protein |
| XAC_RS02105 | XAC0401 | -2.23579 | EscS/YscS/HrcS family type III secretion system export apparatus protein |
| XAC_RS11190 | XAC2202 | -1.67981 | type I secretion system permease/ATPase |
