# Supplementary material for: RNA-seq analysis provides insights into cold stress responses of Xanthomonas citri pv. citri
Source: BMC Genomics. 2019 Nov 6;20:807. doi: 10.1186/s12864-019-6193-0 (PMC6833247; doi:10.1186/s12864-019-6193-0)
Supplement: Supplementary file 11 — Additional file 11: Figure S1. Low temperatures effected Xcc swarming motility. [file 12864_2019_6193_MOESM11_ESM.docx]

**Figure S1**

**
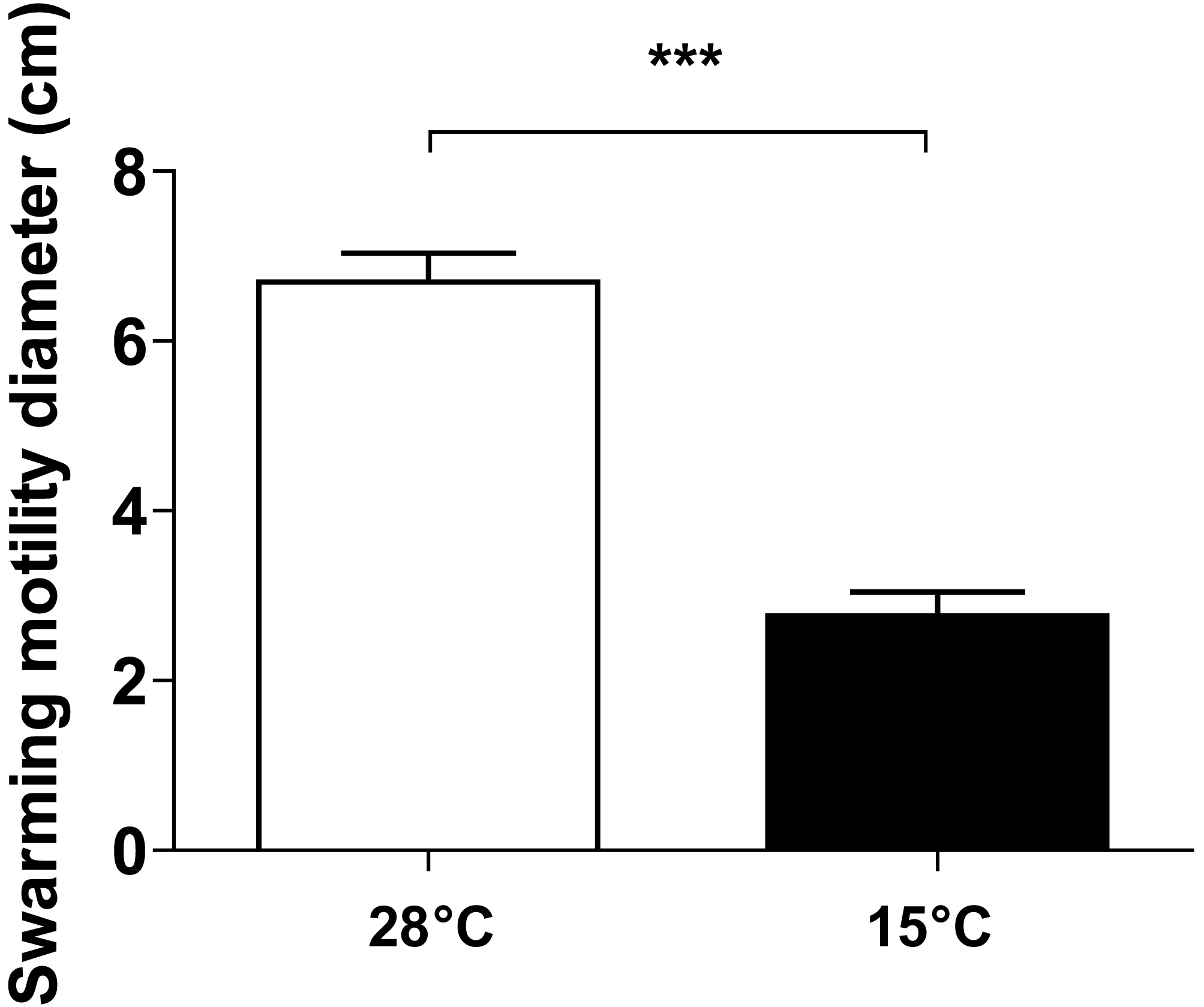
**

**Figure S1. Low temperatures effected *Xcc* swarming** **motility.** Diameters of the motility zones in experiments performed as described in Figure 3**.** Error bars, means ± standard deviations. (“***” stands for p-value < 0.001)
